# Supplementary material for: Beyond the Warburg Effect: Oxidative and Glycolytic Phenotypes Coexist within the Metabolic Heterogeneity of Glioblastoma
Source: Cells. 2021 Jan 20;10(2):202. doi: 10.3390/cells10020202 (PMC7922554; doi:10.3390/cells10020202)
Supplement: Supplementary file 1 [file cells-10-00202-s001.pdf]

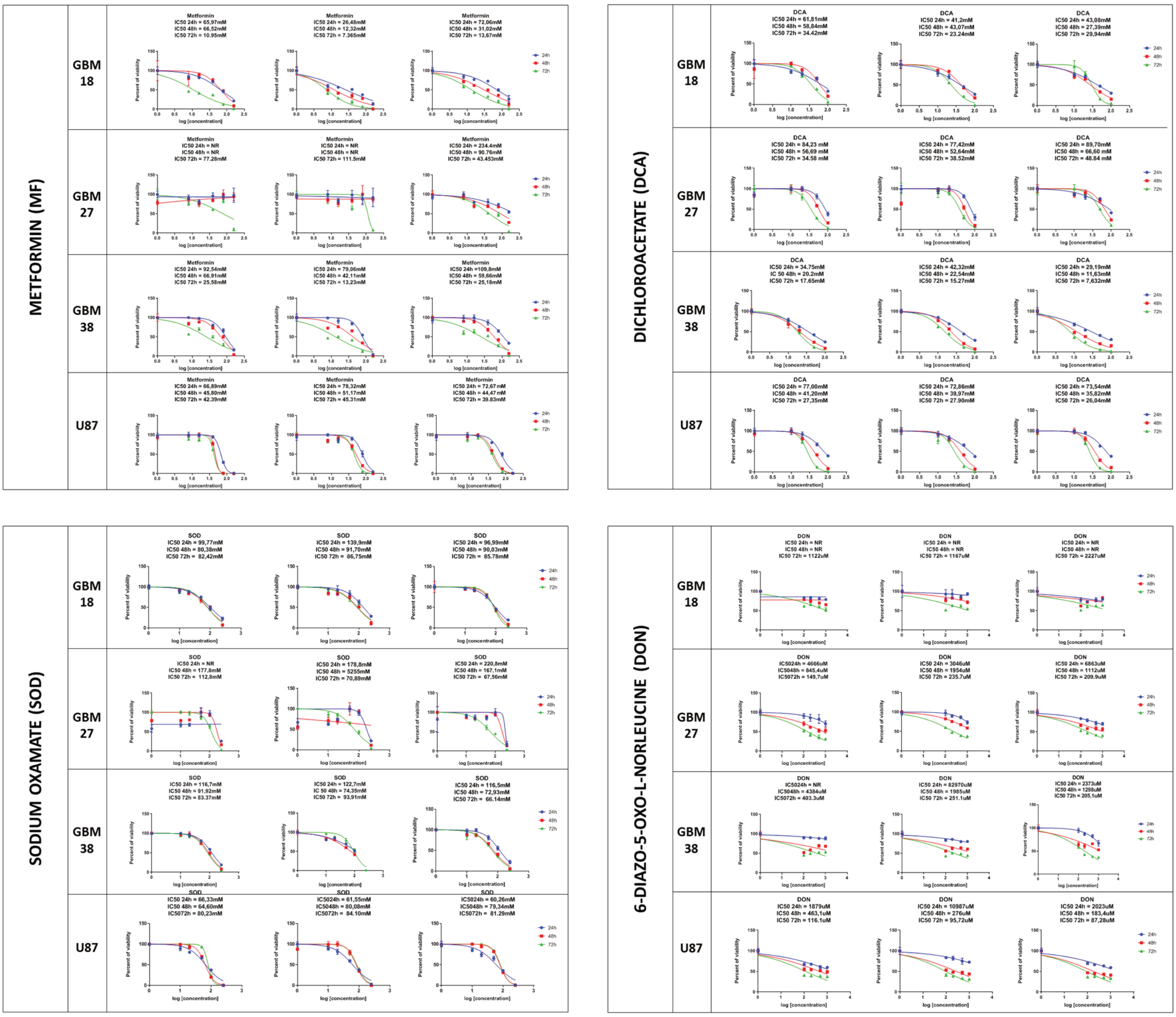

Figure S1: Inhibitory curves for MF, DCA, SOD and DON at 24h, 48h, 72h. Calculated IC50s for each cell line and time-point are included. Abbreviations: NR (IC50 not reached, or unable to extrapolate from data using the logarithmic variable slope equation).

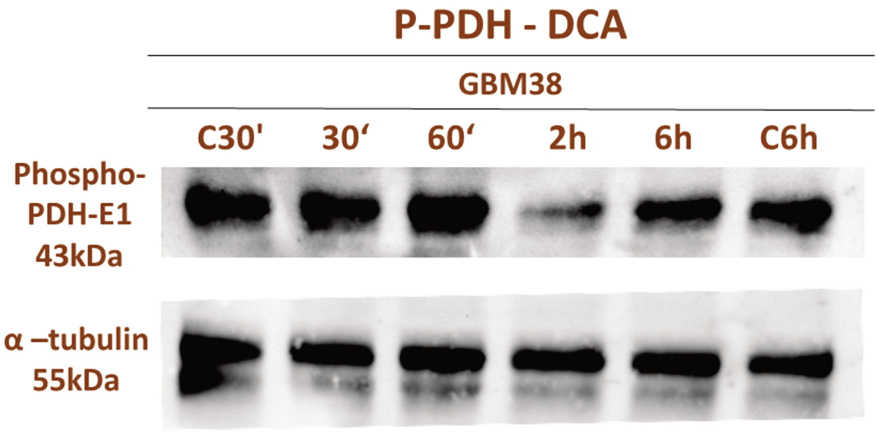

Figure S2: Western Blot time-course analysis of phospho-Ser293 PDH-E1 in GBM38. Protein was collected as follows: 30 min after addition of fresh culture media (C30'); 30 min, 60 min, 2 h and 6 h after addition of DCA 72h-IC50 treatment, and 6h after addition of fresh culture media (C6h).

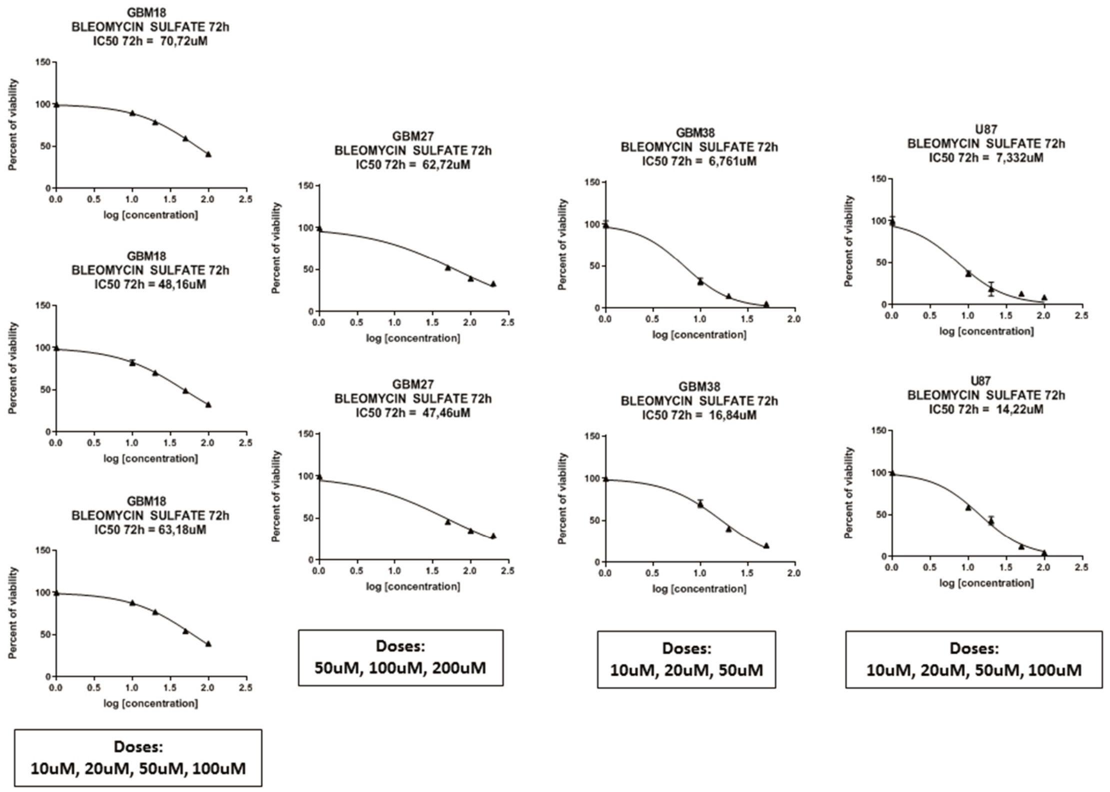

Figure S3: **Inhibitory curves for bleomycin at 72h.** Calculated IC50s for each cell line using the logarithmic variable slope equation are included. A minimum of 2 biological replicates were carried out for each cell line.

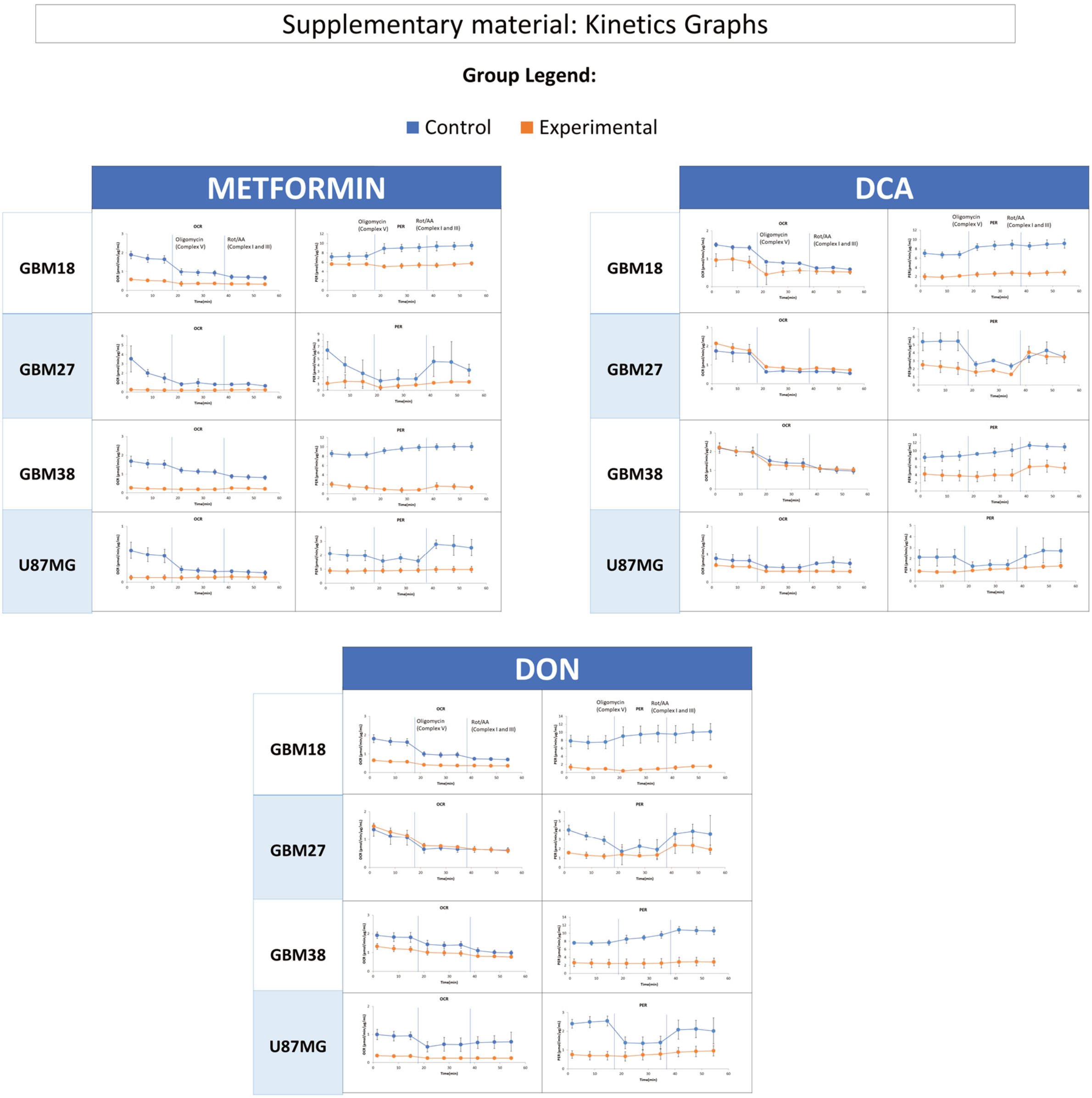

Figure S4: Seahorse XF real-time kinetic graphs. As MF significantly reduced basal OXPHOS in GBM18, glycolysis levels (PER) did not increase after injection of oligomycin; however, PER levels were maintained in treated cells. In GBM27, GBM38 and U87MG, there was a significant reduction of OCR and PER, and, similarly, levels did not change after injection of inhibitors. In terms of OCR changes, DCA treated and non-treated cells responded equally. GBM18, GBM38 and U87MG were not able to compensate total respiration inhibition (rotenone/antimycin A), but, interestingly, GBM27 was able to rescue inhibition by glycolysis, raising to equal levels as control cells, even though basal PER levels were initially lower. DON reduced OCR in GBM18 and U87MG, and, in these cell lines, PER was low and did not change after any injection of mitochondrial inhibitors. In GBM27 and GBM38, OCR fluctuations after inhibitors were similar in control and treated cells; surprisingly, DON did not reduce OCR in GBM27 at all (inhibition of glutaminolysis had no effect on mitochondrial ATP production rates). However, PER increased in GBM27 in both control and treated cells after rotenone/antimycin A, whereas, in GBM38, PER was globally decreased and remained unaltered even after injection of inhibitors. Kinetic profiles of OCR and ECAR also allow for the identification of resistance to ETC inhibition and residual non-mitochondrial OCR (i.e., after injection of rotenone/antimycin A, OCR does not reach zero). In our study, cells in basal conditions often maintained residual OCR, whereas MF treated cells demonstrated nearly completely abolished OCR. Abbreviations: Proton Efflux Rate (PER), Oxygen Consumption Rate (OCR).

File S1: Comprehensive analysis of Chou-Talalay combinatory experiments, as exported from CompuSyn software. Fraction of affected cells from each drug individually and in combination, with median-effect dose (Dm), sigmodicity/shape (m) and linear correlation coefficient of the median-effect plot (r). Additionally, we provide complete descriptions of Dose-Effect Curve, Median-Effect Plot, Combination Index Plot, Logarithmic Combination Index Plot, DRI Plot, Log(DRI) Plot, Isobologram and Polygonogram at Fa = 0.9. Reports are provided in PDF format.

…Data for Drug: MF [uM]

Dose	Effect
7870.0	0.53624
7870.0	0.57607
7870.0	0.41415
15750.0	0.58159
15750.0	0.71024
15750.0	0.56295
39390.0	0.62561
39390.0	0.81219
39390.0	0.66741
78780.0	0.87241
78780.0	0.92948
78780.0	0.79514
157520.	0.98401
157520.	0.99
157520.	0.98892
10660.0	0.52521
10660.0	0.45177

17 data points entered.

X-int: 4.04414

Y-int: -5.3314 +/- 0.74215

m: 1.31830 +/- 0.16423

Dm: 11069.8

r: 0.90065

Data for Drug: BL [uM]

Dose	Effect
60.5	0.5028
10.0	0.17796
10.0	0.11899
20.0	0.29743
20.0	0.23117
50.0	0.50862
50.0	0.45673
100.0	0.67143
100.0	0.60417
60.5	0.55855

10 data points entered.

X-int: 1.74071

Y-int: -1.7832 +/- 0.11170

m: 1.02439 +/- 0.06994

Dm: 55.0440

r: 0.98186

Data for Drug Combo: MF-BL (MF+BL [176.198:1])

Dose A	Effect
1332.50+	0.18353
5330.00+	0.50214
10660.0+	0.67842
10660.0+	0.69075
5330.00+	0.52958
1332.50+	0.18896

6 data points entered.

X-int: 3.71365

Y-int: -4.0325 +/- 0.08700

m: 1.08586 +/- 0.02385

Dm: 5171.86

r: 0.99904

Dose-Effect Curve

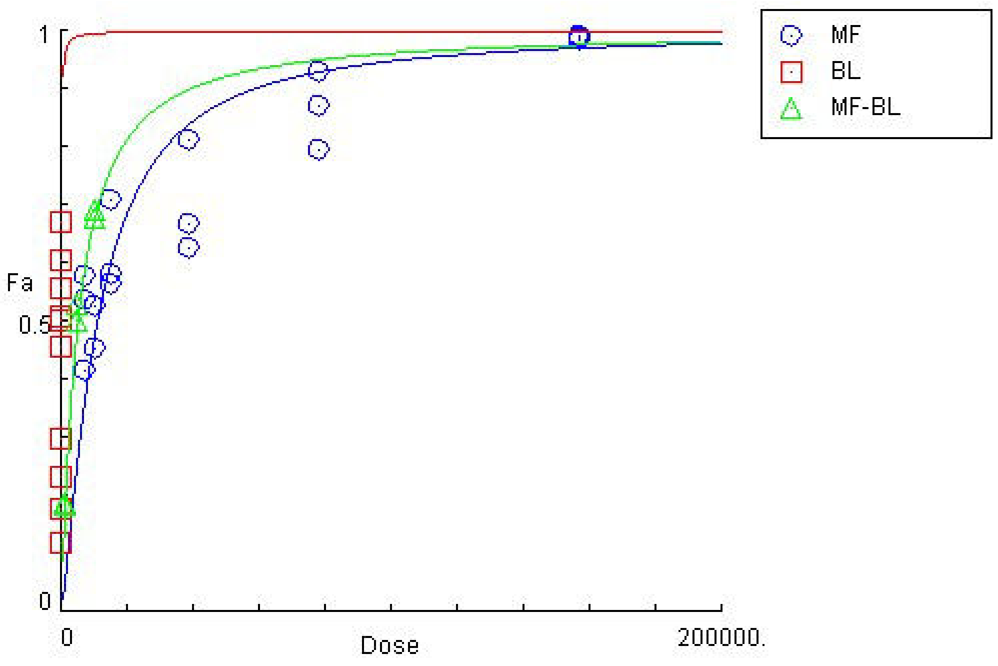

Median-Effect Plot

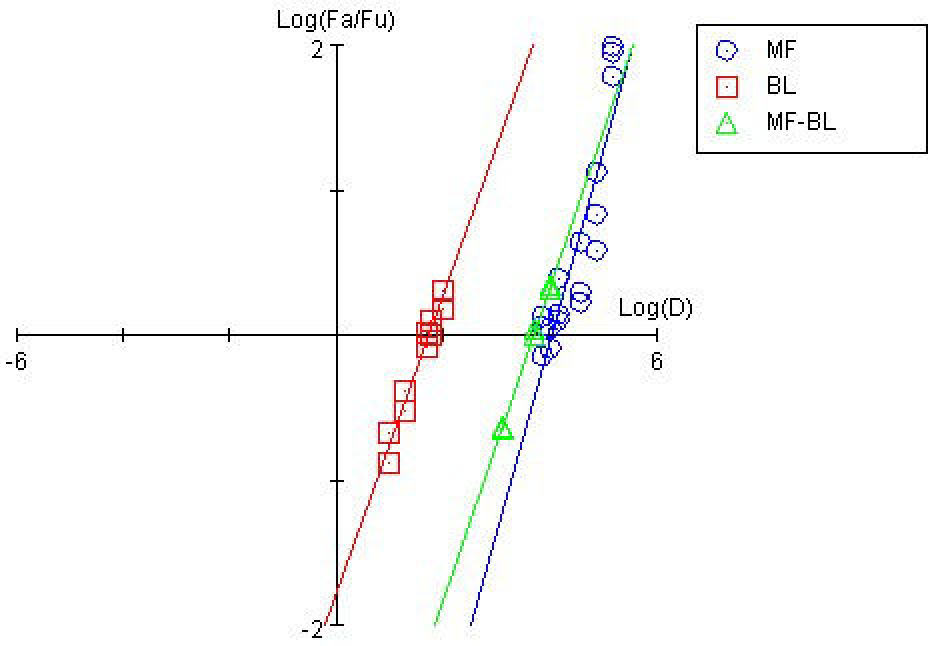

CI Data for Drug Combo: MF-BL (MF+BL [176.198:1])

Fa	CI Value	S.D.A. Analysis
0.05	0.91195	0.91477 +/- 0.07991
0.1	0.92386	0.92581 +/- 0.06149
0.15	0.93412	0.93560 +/- 0.05104
0.2	0.94339	0.94455 +/- 0.04371
0.25	0.95210	0.95301 +/- 0.03809
0.3	0.96052	0.96123 +/- 0.03361
0.35	0.96884	0.96938 +/- 0.03003
0.4	0.97723	0.97762 +/- 0.02726
0.45	0.98583	0.98609 +/- 0.02532
0.5	0.99482	0.99495 +/- 0.02428
0.55	1.00436	1.00438 +/- 0.02424
0.6	1.01468	1.01460 +/- 0.02527
0.65	1.02611	1.02592 +/- 0.02743
0.7	1.03908	1.03880 +/- 0.03078
0.75	1.05431	1.05393 +/- 0.03545
0.8	1.07300	1.07253 +/- 0.04181
0.85	1.09749	1.09695 +/- 0.05065
0.9	1.13336	1.13277 +/- 0.06405
0.95	1.19999	1.19946 +/- 0.08955
0.97	1.25451	1.25415 +/- 0.11081

CI values for actual experimental points:

Total Dose	Fa	CI Value
1340.06	0.18353	0.96333
5360.25	0.50214	1.02336
10720.5	0.67842	1.07696
10720.5	0.69075	1.02503

Total Dose	Fa	CI Value
5360.25	0.52958	0.92968
1340.06	0.18896	0.93302

Combination Index Plot

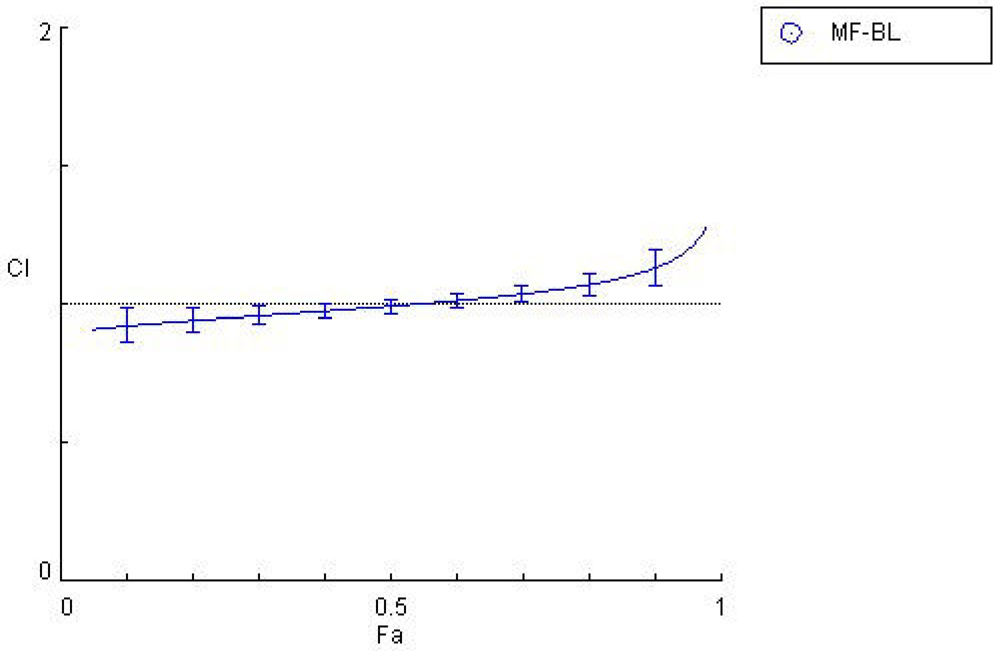

Logarithmic Combination Index Plot

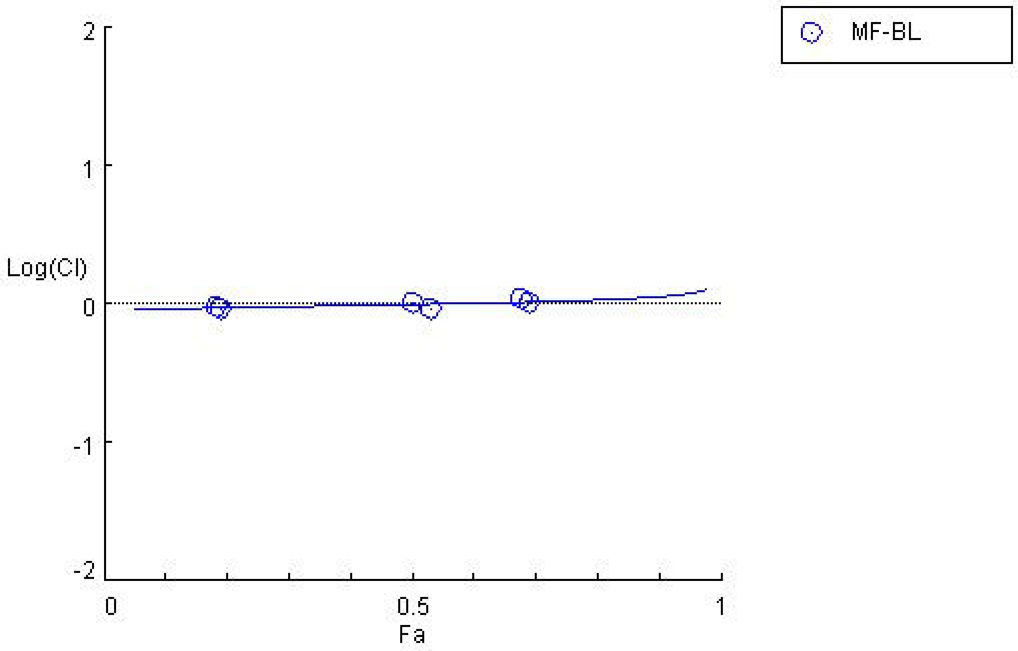

DRI Data for Drug Combo: MF-BL (MF+BL [176.198:1])

Fa	Dose MF	Dose BL	DRI MF	DRI BL
0.05	1186.15	3.10740	3.47214	1.60271
0.1	2090.73	6.44441	3.07540	1.67028
0.15	2969.63	10.1231	2.85284	1.71353
0.2	3867.64	14.2227	2.69596	1.74683

Fa	Dose MF	Dose BL	DRI MF	DRI BL
0.25	4810.82	18.8342	2.57292	1.77482
0.3	5821.17	24.0709	2.47004	1.79964
0.35	6921.60	30.0791	2.38016	1.82249
0.4	8138.89	37.0519	2.29903	1.84413
0.45	9506.74	45.2516	2.22383	1.86512
0.5	11069.8	55.0440	2.15254	1.88591
0.55	12889.8	66.9553	2.08352	1.90694
0.6	15056.1	81.7728	2.01538	1.92865
0.65	17704.0	100.729	1.94668	1.95155
0.7	21050.8	125.871	1.87585	1.97632
0.75	25471.8	160.869	1.80084	2.00396
0.8	31683.4	213.028	1.71865	2.03607
0.85	41264.5	299.298	1.62414	2.07564
0.9	58611.1	470.149	1.50660	2.12939
0.95	103309.	975.039	1.33445	2.21916
0.97	154627.	1638.41	1.22408	2.28532

DRI values calculated at experimental points

Fa	Dose MF	Dose BL	DRI MF	DRI BL
0.18353	3567.97	12.8206	2.67766	1.69528
0.50214	11141.9	55.5059	2.09041	1.83490
0.67842	19501.5	114.078	1.82941	1.88558
0.69075	20364.9	120.618	1.91040	1.99369
0.52958	12110.4	61.7906	2.27211	2.04266
0.18896	3666.35	13.2773	2.75149	1.75567

DRI Plot for Combo: MF-BL (MF+BL [176.198:1])

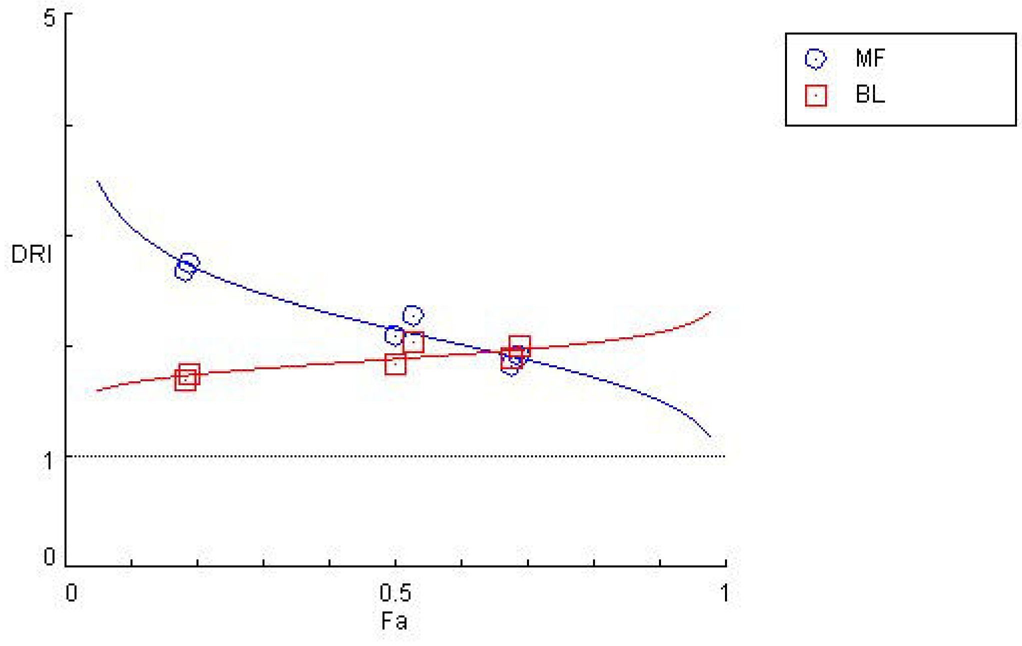

Log(DRI) Plot for Combo: MF-BL (MF+BL [176.198:1])

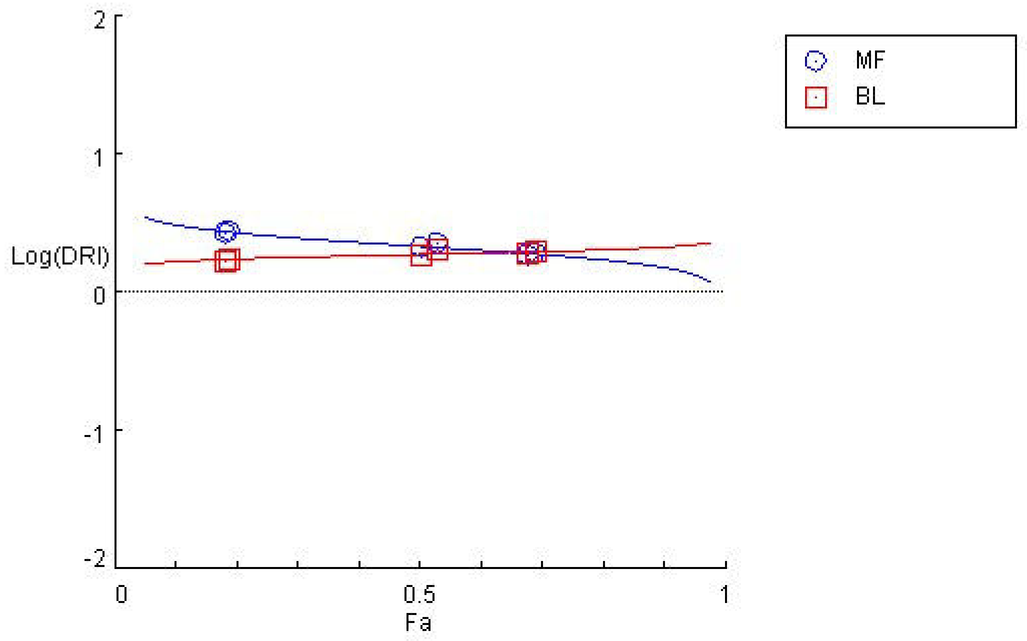

Isobologram for Combo: MF-BL (MF+BL [176.198:1])

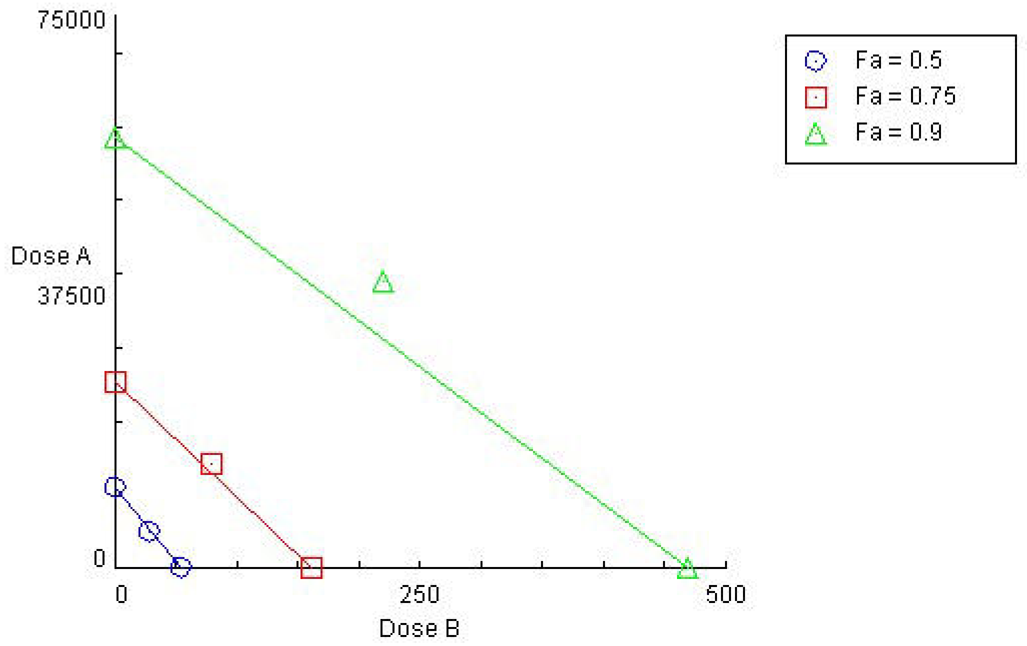

Polygonogram at Fa = 0.9

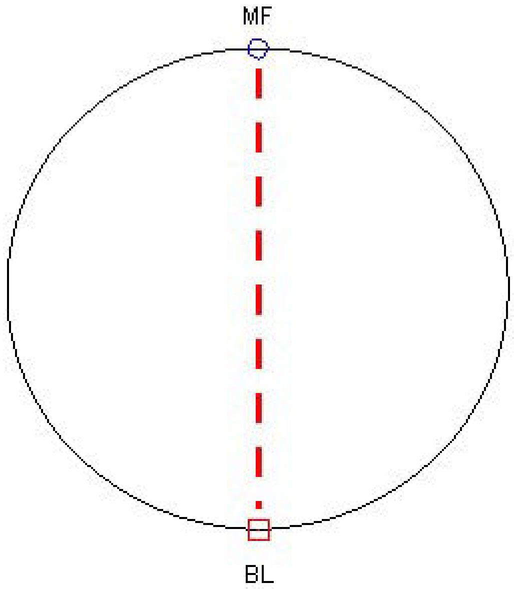
MF
BL

…Data for Drug: MF [uM]

Dose	Effect
7870.0	0.23281
7870.0	0.14046
7870.0	0.10309
15750.0	0.25291
15750.0	0.17587
15750.0	0.30018
39390.0	0.39243
39390.0	0.35269
39390.0	0.48068
78780.0	0.16058
78780.0	0.07686
78780.0	0.51773
157520.	0.90852
157520.	0.91911
157520.	0.95778
77411.0	0.43052
77411.0	0.39409

17 data points entered.

X-int: 4.76061
Y-int: -4.9332 +/- 1.30053
m: 1.03625 +/- 0.28164
Dm: 57624.7
r: 0.68875

Data for Drug: BL [uM]

Dose	Effect
50.0	0.47634
50.0	0.54304
100.0	0.60608
100.0	0.64751
200.0	0.66695
200.0	0.70512
55.09	0.61750
55.09	0.60433

8 data points entered.

X-int: 1.47042
Y-int: -0.6157 +/- 0.23160
m: 0.41874 +/- 0.11876
Dm: 29.5408
r: 0.82127

Data for Drug Combo: MF-BL (MF+BL [1404.97:1])

Dose A	Effect

Dose A	Effect
77411.0+	0.73208
38705.5+	0.69087
9676.37+	0.24292
77411.0+	0.79116
38705.5+	0.63240
9676.37+	0.22696

6 data points entered.

X-int: 4.40525

Y-int: -5.1086 +/- 0.47342

m: 1.15966 +/- 0.10512

Dm: 25424.3

r: 0.98396

Dose-Effect Curve

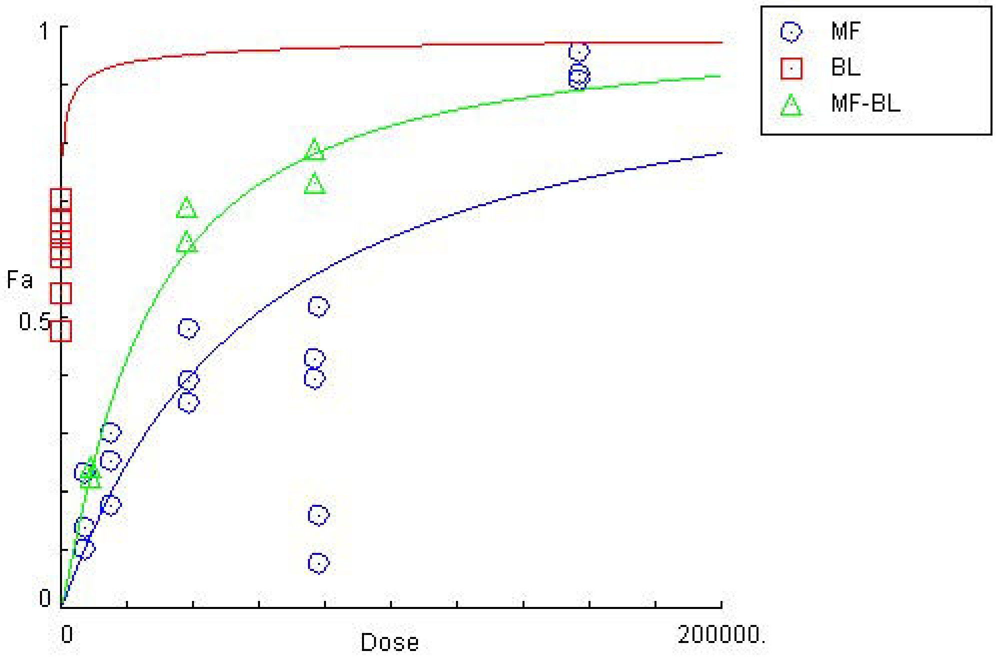

Median-Effect Plot

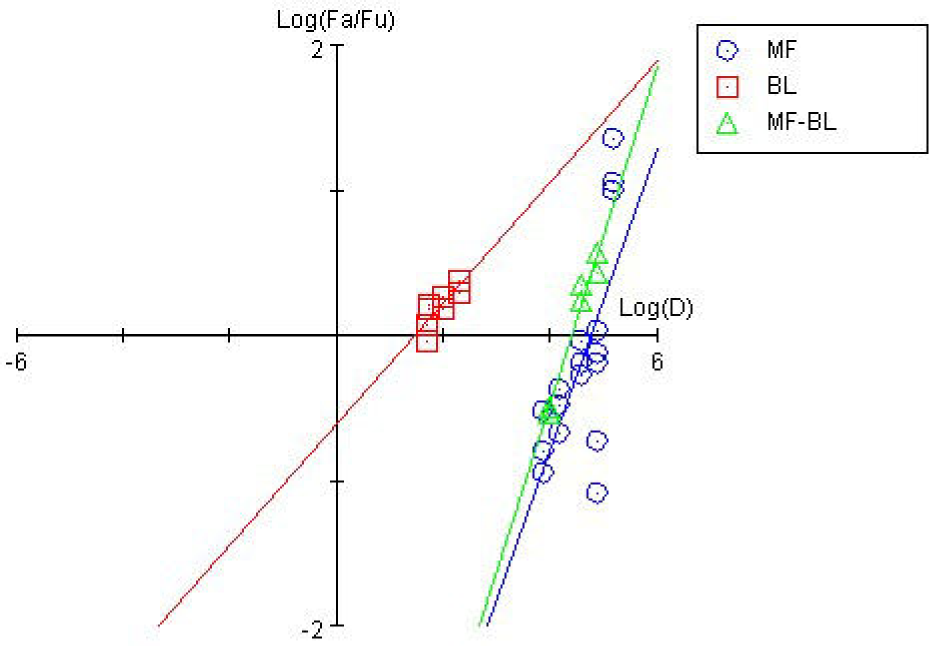

CI Data for Drug Combo: MF-BL (MF+BL [1404.97:1])

Fa	CI Value	S.D.A. Analysis
0.05	55.2970	72.4175 +/- 199.367
0.1	18.0448	20.9963 +/- 36.4717
0.15	9.16239	10.1143 +/- 12.4923
0.2	5.58389	5.97620 +/- 5.48397
0.25	3.76582	3.94837 +/- 2.73350
0.3	2.71103	2.80136 +/- 1.46224
0.35	2.04403	2.08976 +/- 0.81237
0.4	1.59604	1.61894 +/- 0.45859
0.45	1.28150	1.29237 +/- 0.25991
0.5	1.05303	1.05755 +/- 0.15023
0.55	0.88259	0.88386 +/- 0.09773
0.6	0.75265	0.75242 +/- 0.08254
0.65	0.65177	0.65102 +/- 0.08448
0.7	0.57218	0.57147 +/- 0.09066
0.75	0.50836	0.50802 +/- 0.09716
0.8	0.45621	0.45642 +/- 0.10361
0.85	0.41234	0.41325 +/- 0.11059
0.9	0.37325	0.37508 +/- 0.11905
0.95	0.33269	0.33600 +/- 0.13097
0.97	0.31157	0.31599 +/- 0.13813

CI values for actual experimental points:

Total Dose	Fa	CI Value
77466.1	0.73208	0.67833
38733.0	0.69087	0.44577
9683.26	0.24292	4.02328
77466.1	0.79116	0.44900

Total Dose	Fa	CI Value
38733.0	0.63240	0.65317
9683.26	0.22696	4.90037

Combination Index Plot

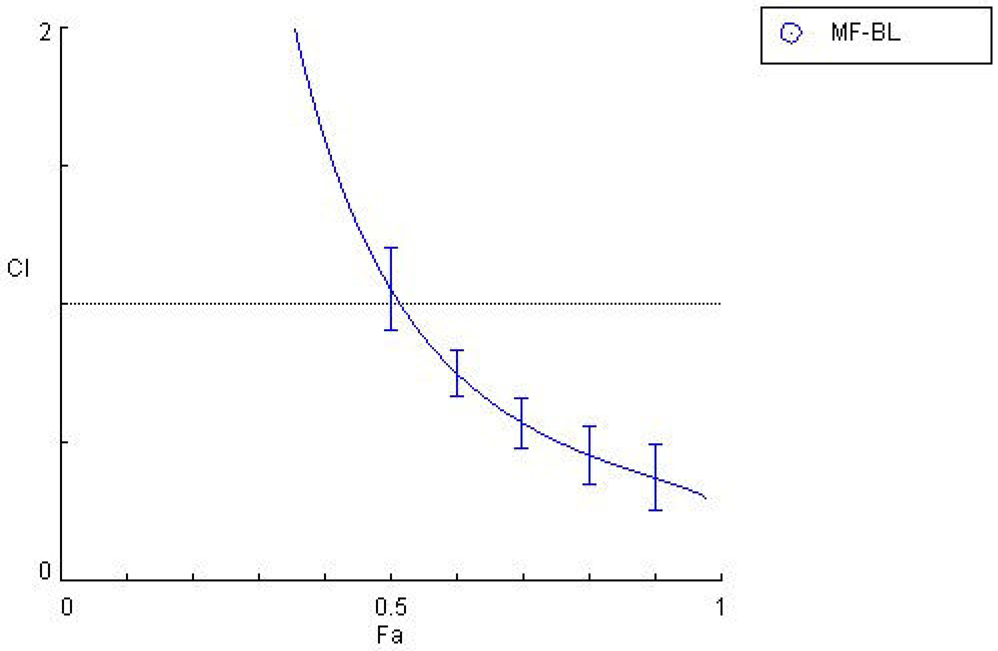

Logarithmic Combination Index Plot

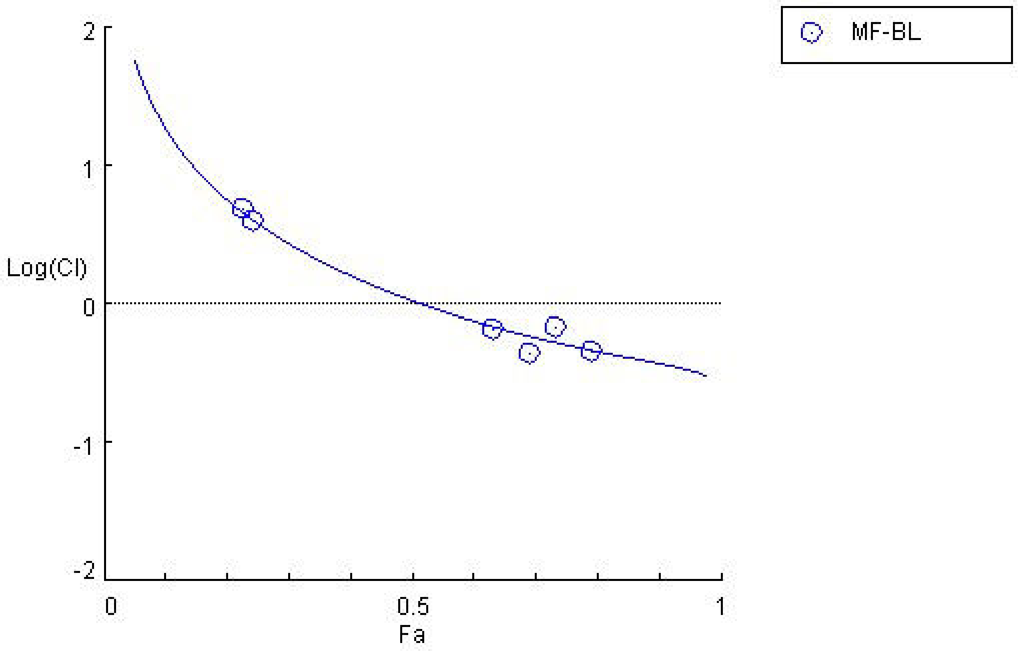

DRI Data for Drug Combo: MF-BL (MF+BL [1404.97:1])

Fa	Dose MF	Dose BL	DRI MF	DRI BL
0.05	3361.91	0.02610	1.67626	0.01828
0.1	6914.26	0.15544	1.80996	0.05717
0.15	10805.2	0.46922	1.89803	0.11580
0.2	15122.0	1.07801	1.96715	0.19702

Fa	Dose MF	Dose BL	DRI MF	DRI BL
0.25	19960.8	2.14286	2.02613	0.30560
0.3	25439.2	3.90521	2.07911	0.44842
0.35	31707.9	6.73572	2.12842	0.63525
0.4	38965.2	11.2174	2.17562	0.87997
0.45	47479.6	18.2935	2.22187	1.20275
0.5	57624.7	29.5408	2.26813	1.63361
0.55	69937.5	47.7034	2.31536	2.21883
0.6	85219.8	77.7951	2.36457	3.03272
0.65	104725.	129.557	2.41701	4.20104
0.7	130531.	223.461	2.47434	5.95132
0.75	166357.	407.242	2.53903	8.73268
0.8	219588.	809.512	2.61517	13.5451
0.85	307316.	1859.82	2.71040	23.0456
0.9	480255.	5614.13	2.84229	46.6816
0.95	987714.	33439.4	3.06899	145.979
0.97	1649876	119034.	3.24121	328.545

DRI values calculated at experimental points

Fa	Dose MF	Dose BL	DRI MF	DRI BL
0.73208	152017.	325.816	1.96376	5.91339
0.69087	125212.	201.600	3.23500	7.31789
0.24292	19239.9	1.95641	1.98834	0.28406
0.79116	208369.	710.984	2.69172	12.9040
0.63240	97271.8	107.923	2.51313	3.91752
0.22696	17659.1	1.58240	1.82497	0.22976

DRI Plot for Combo: MF-BL (MF+BL [1404.97:1])

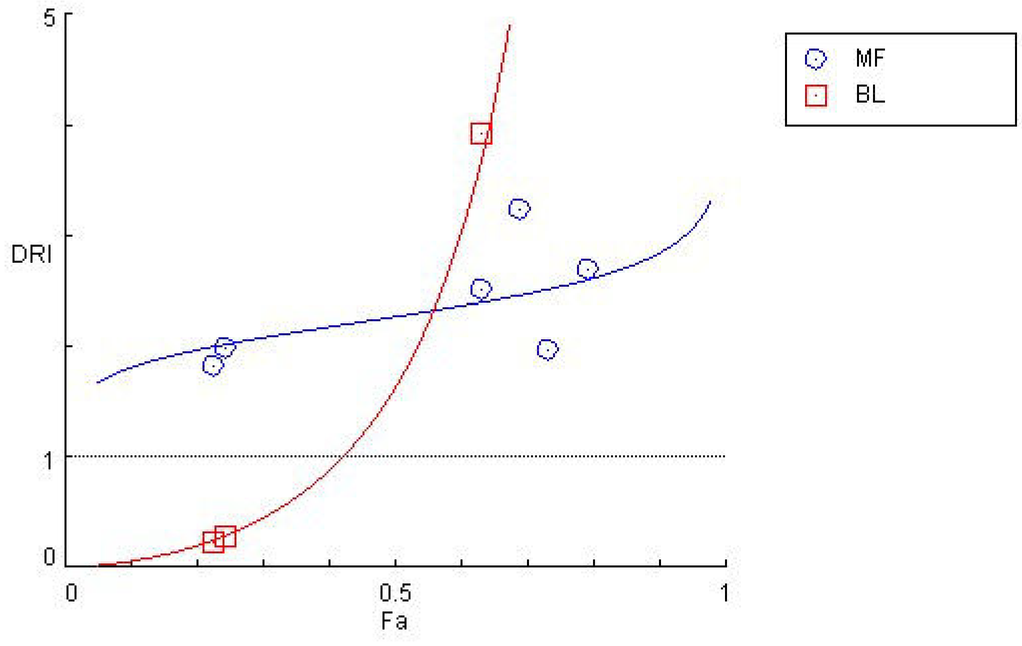

Log(DRI) Plot for Combo: MF-BL (MF+BL [1404.97:1])

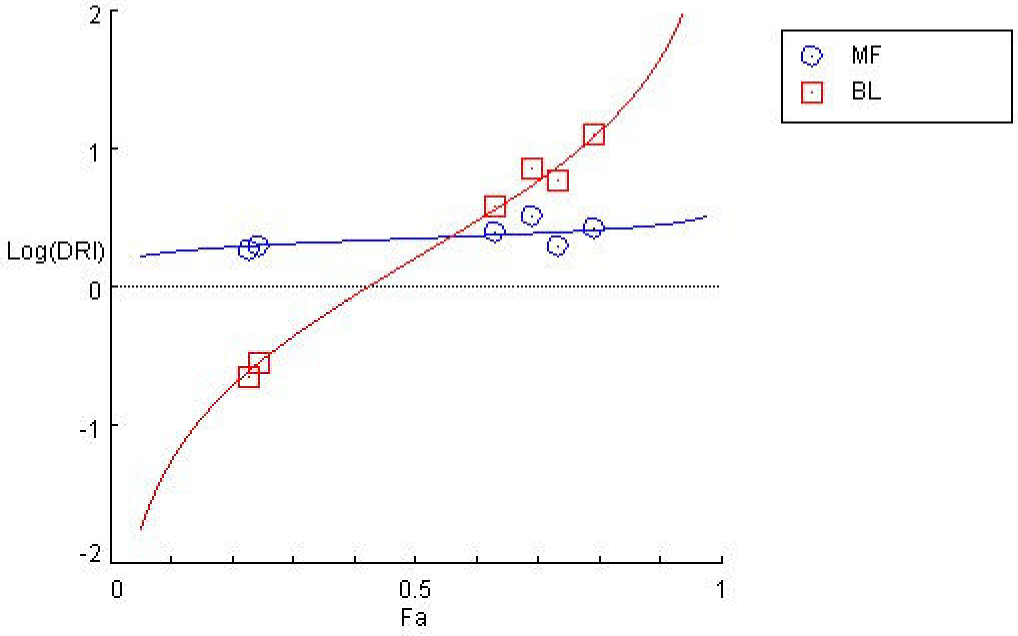

Isobologram for Combo: MF-BL (MF+BL [1404.97:1])

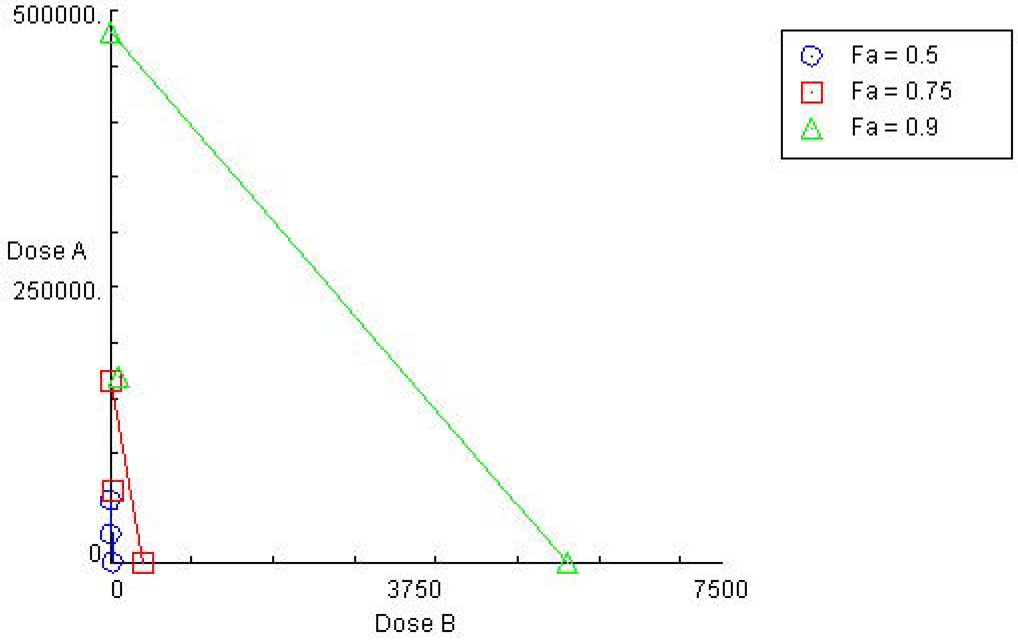

Polygonogram at Fa = 0.9

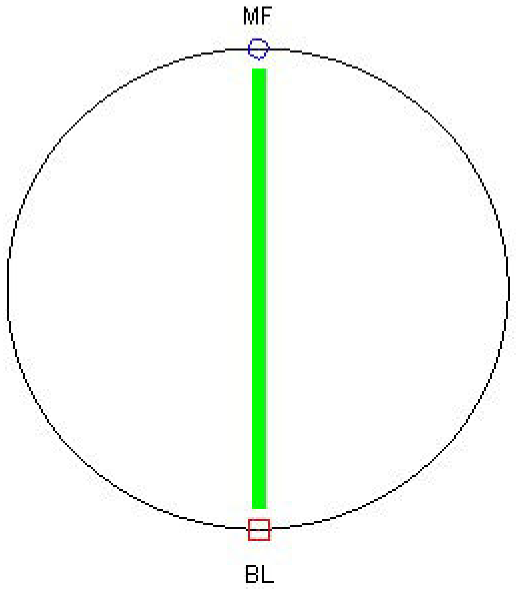
MF
BL

…Data for Drug: MF [uM]

Dose	Effect
7870.0	0.42573
7870.0	0.52827
7870.0	0.38937
15750.0	0.29622
15750.0	0.49188
15750.0	0.35812
39390.0	0.49697
39390.0	0.57784
39390.0	0.46921
78780.0	0.70248
78780.0	0.87161
78780.0	0.72910
157520.	0.999
157520.	0.99754
157520.	0.98194
21330.0	0.44783
21330.0	0.63190

17 data points entered.

X-int: 4.24993
Y-int: -7.3975 +/- 1.55867
m: 1.74061 +/- 0.34245
Dm: 17779.8
r: 0.79541

Data for Drug: BL [uM]

Dose	Effect
50.0	0.68241
50.0	0.29706
100.0	0.85466
100.0	0.59909
200.0	0.94684
200.0	0.79812
11.8	0.69459
11.8	0.76364

8 data points entered.

X-int: 0.52962
Y-int: -0.1933 +/- 0.66703
m: 0.36498 +/- 0.36539
Dm: 3.38545
r: 0.37760

Data for Drug Combo: MF-BL (MF+BL [1807.62:1])

Dose A	Effect

**Dose A**	**Effect**
21330.0+	0.61629
10665.0+	0.55014
2666.25+	0.48829
21330.0+	0.81932
10665.0+	0.68451
2666.25+	0.55647

6 data points entered.

X-int: 3.37748

Y-int: -1.3953 +/- 0.83013

m: 0.41313 +/- 0.21039

Dm: 2384.95

r: 0.70060

Dose-Effect Curve

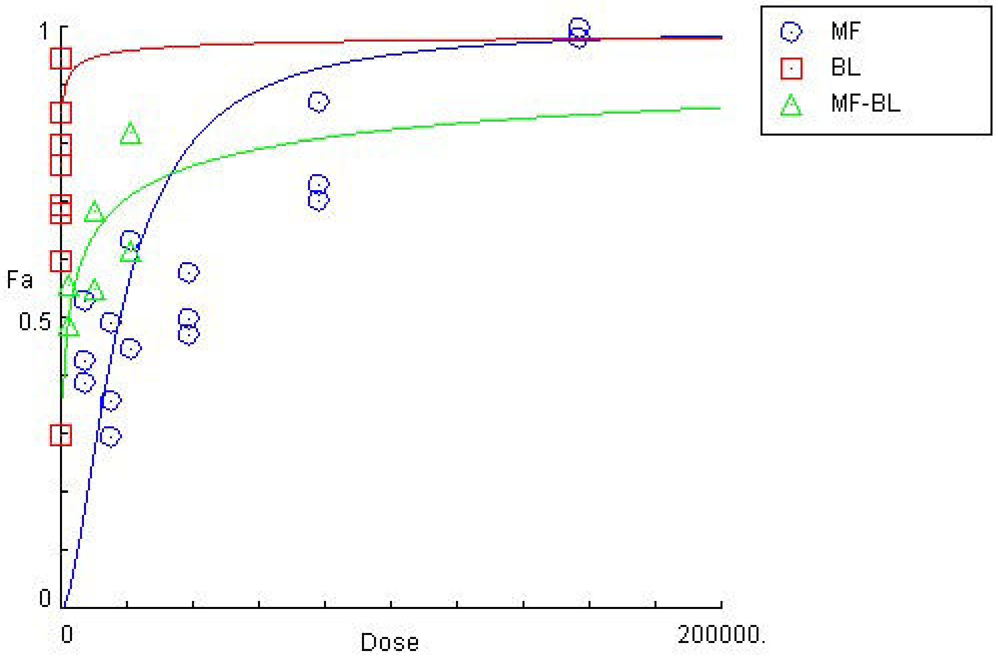

Median-Effect Plot

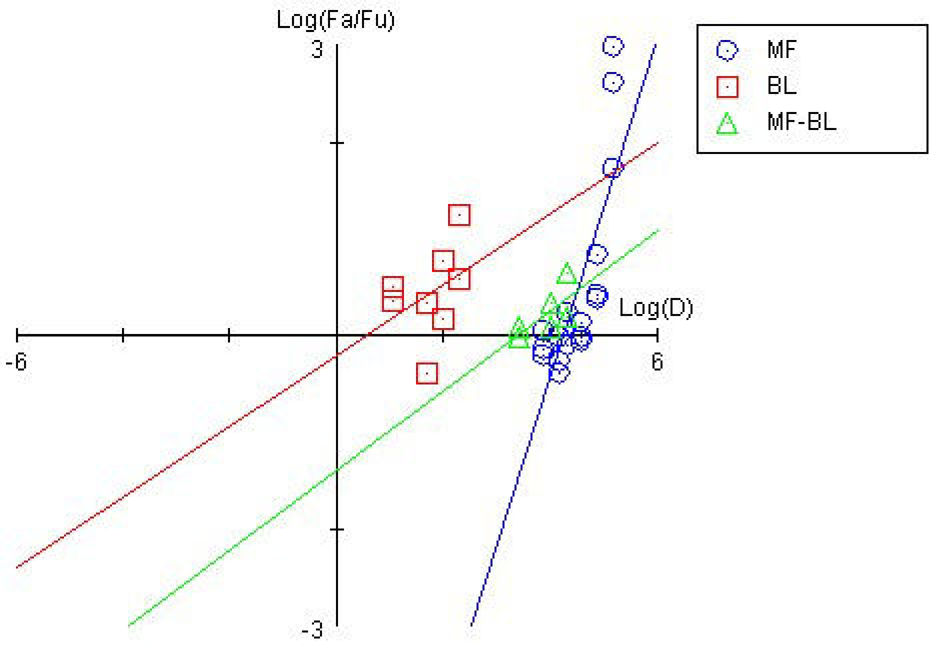

CI Data for Drug Combo: MF-BL (MF+BL [1807.62:1])

Fa	CI Value	S.D.A. Analysis
0.05	0.99797	1.35E12 +/- 1.54E13
0.1	0.78798	1.726E9 +/- 1.97E10
0.15	0.68322	2.789E7 +/- 3.187E8
0.2	0.61679	1248703 +/- 1.427E7
0.25	0.57083	96011.3 +/- 1097274
0.3	0.53859	10210.6 +/- 116687.
0.35	0.51740	1334.12 +/- 15241
0.4	0.50677	199.068 +/- 2269.11
0.45	0.50785	32.4770 +/- 365.246
0.5	0.52357	5.86109 +/- 60.9043
0.55	0.55951	1.45038 +/- 10.0737
0.6	0.62559	0.77514 +/- 1.54372
0.65	0.73999	0.78587 +/- 0.44716
0.7	0.93781	1.01305 +/- 1.00953
0.75	1.29307	1.50231 +/- 2.66111
0.8	1.98294	2.65178 +/- 8.11193
0.85	3.51983	6.21459 +/- 31.7206
0.9	7.93559	25.0526 +/- 197.844
0.95	30.9088	370.205 +/- 3885.51
0.97	82.1983	2923.55 +/- 32488.4

CI values for actual experimental points:

Total Dose	Fa	CI Value
21341.8	0.61629	1.86538
10670.9	0.55014	1.53851
2667.73	0.48829	0.64941
21341.8	0.81932	0.55875

Total Dose	Fa	CI Value
10670.9	0.68451	0.59312
2667.73	0.55647	0.36566

Combination Index Plot

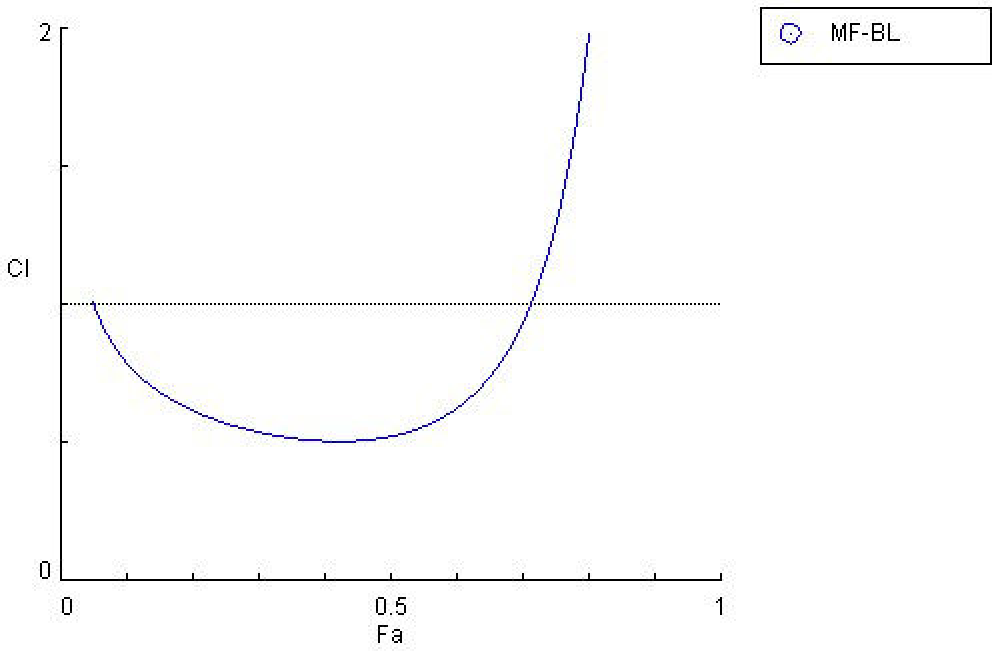

Logarithmic Combination Index Plot

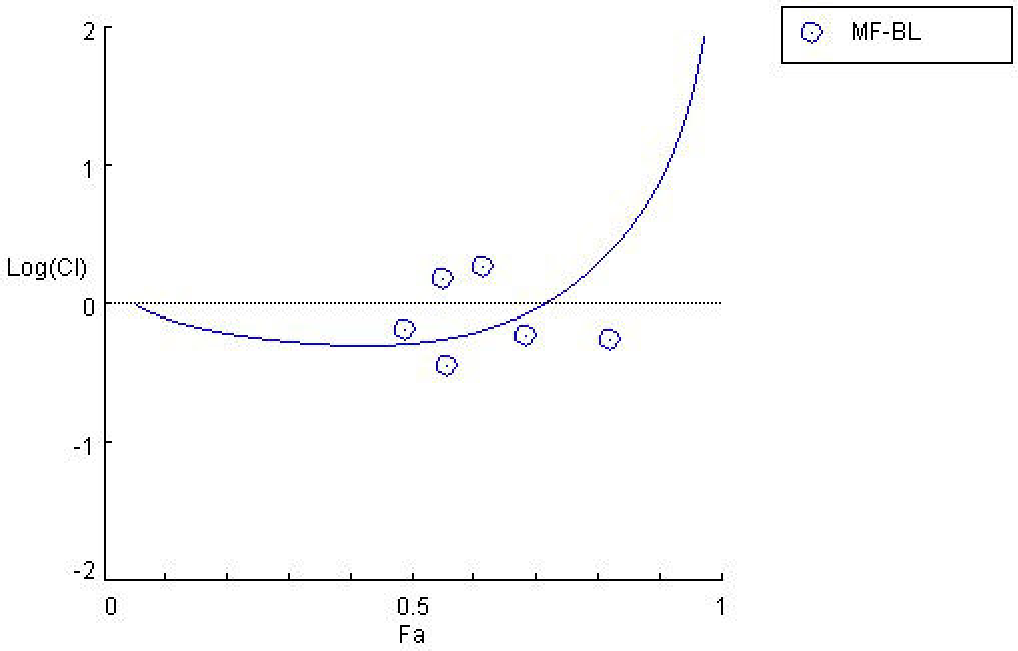

DRI Data for Drug Combo: MF-BL (MF+BL [1807.62:1])

Fa	Dose MF	Dose BL	DRI MF	DRI BL
0.05	3275.42	0.00106	1711.25	1.00262
0.1	5031.56	0.00822	430.778	1.27281
0.15	6563.43	0.02921	183.382	1.47544
0.2	8017.44	0.07587	96.4077	1.64903

Fa	Dose MF	Dose BL	DRI MF	DRI BL
0.25	9458.33	0.16686	56.6853	1.80769
0.3	10927.5	0.33220	35.6439	1.95874
0.35	12458.7	0.62089	23.3876	2.10685
0.4	14085.1	1.11467	15.7673	2.25555
0.45	15843.7	1.95360	10.8036	2.40799
0.5	17779.8	3.38545	7.45910	2.56735
0.55	19952.4	5.86675	5.14996	2.73725
0.6	22443.6	10.2822	3.52871	2.92224
0.65	25373.5	18.4595	2.37897	3.12849
0.7	28929.0	34.5008	1.56095	3.36505
0.75	33422.4	68.6866	0.98153	3.64623
0.8	39429.1	151.074	0.57711	3.99706
0.85	48163.9	392.324	0.30340	4.46731
0.9	62827.5	1393.56	0.12916	5.17850
0.95	96512.8	10795.6	0.03251	6.57402
0.97	130990.	46331.7	0.01218	7.79047

DRI values calculated at experimental points

Fa	Dose MF	Dose BL	DRI MF	DRI BL
0.61629	23342.6	12.4002	1.09436	1.05086
0.55014	19958.7	5.87559	1.87142	0.99586
0.48829	17307.7	2.97766	6.49137	2.01874
0.81932	42375.5	213.035	1.98666	18.0538
0.68451	27745.0	28.2670	2.60150	4.79100
0.55647	20254.6	6.30281	7.59665	4.27306

DRI Plot for Combo: MF-BL (MF+BL [1807.62:1])

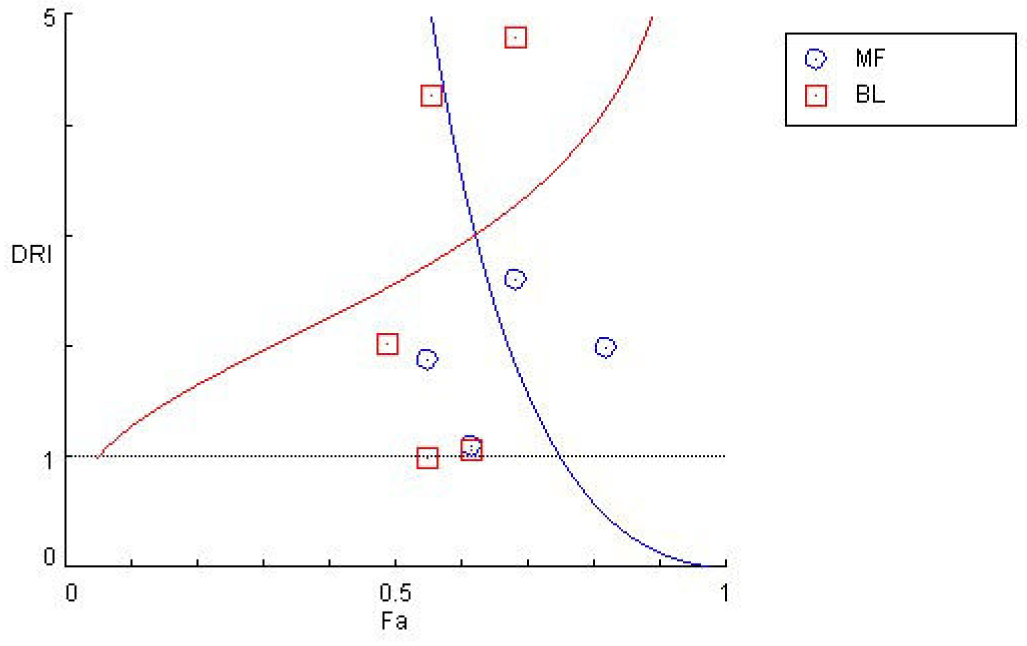

Log(DRI) Plot for Combo: MF-BL (MF+BL [1807.62:1])

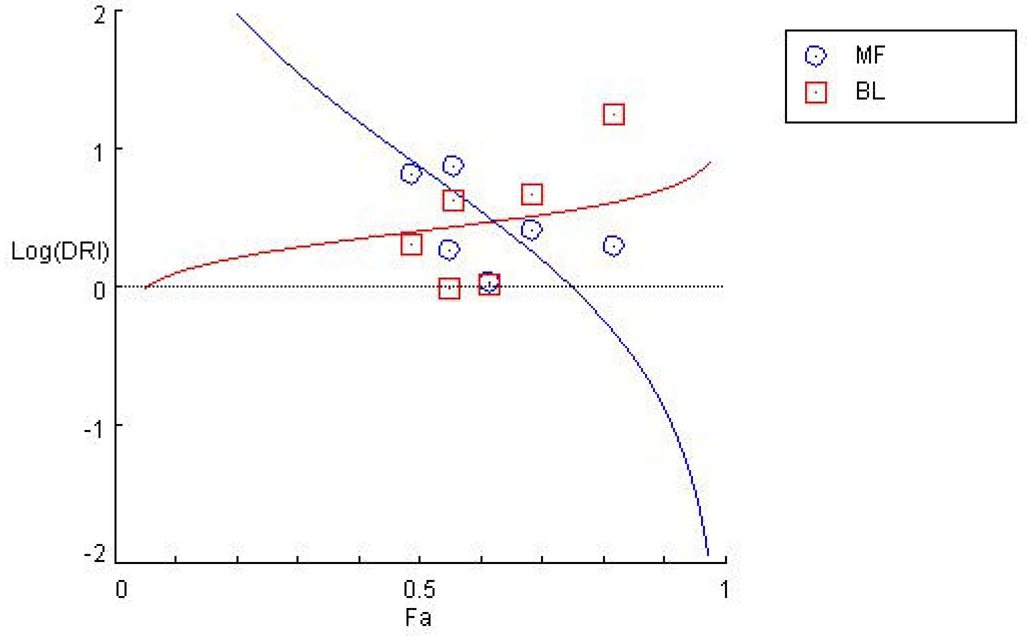

Isobologram for Combo: MF-BL (MF+BL [1807.62:1])

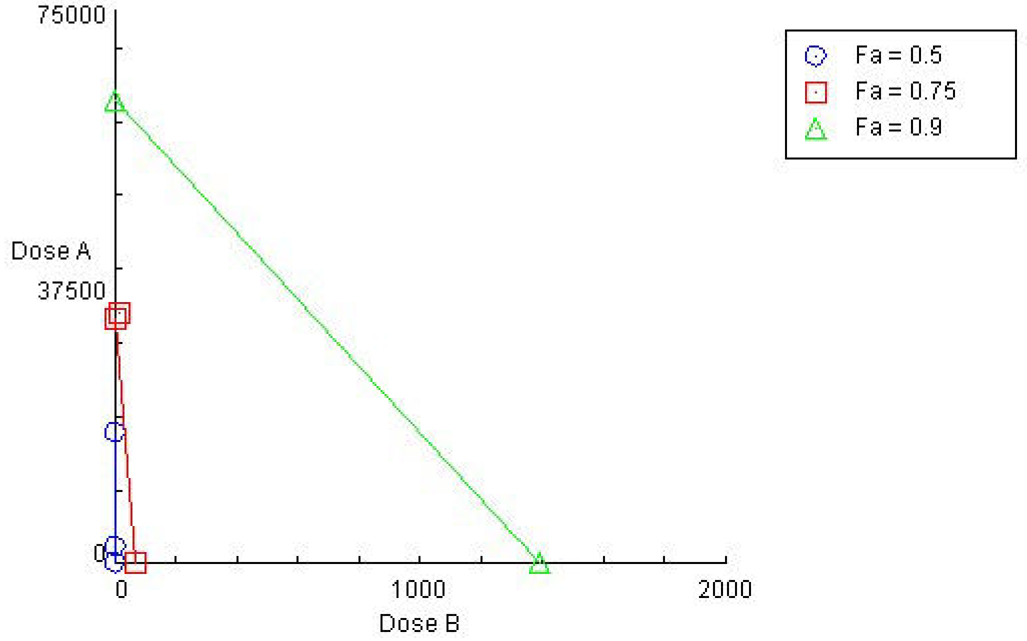

Polygonogram at Fa = 0.9

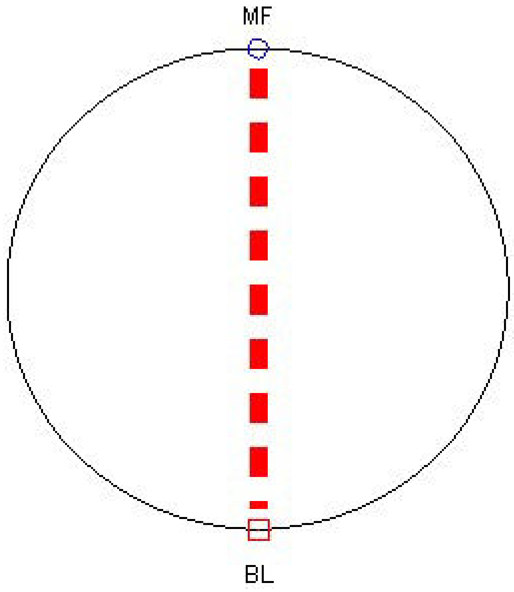
MF
BL

…Data for Drug: MF [uM]

Dose	Effect
7870.0	0.11052
7870.0	0.17368
7870.0	0.11707
15750.0	0.12162
15750.0	0.16401
15750.0	0.08268
39390.0	0.37882
39390.0	0.33214
39390.0	0.66741
78780.0	0.99824
78780.0	0.94378
78780.0	0.95238
157520.	0.99816
157520.	0.99783
157520.	0.98453
42510.0	0.48319
42510.0	0.49056

17 data points entered.

X-int: 4.40963
Y-int: -11.934 +/- 1.61396
m: 2.70630 +/- 0.35193
Dm: 25682.3
r: 0.89312

Data for Drug: BL [uM]

Dose	Effect
10.0	0.63176
10.0	0.41297
20.0	0.81331
20.0	0.56917
50.0	0.86886
50.0	0.88044
100.0	0.91241
100.0	0.95258
10.241	0.60008
10.241	0.71813

10 data points entered.

X-int: 0.86194
Y-int: -0.8670 +/- 0.24348
m: 1.00590 +/- 0.16722
Dm: 7.27686
r: 0.90495

Data for Drug Combo: MF-BL (MF+BL [4150.96:1])

Dose A	Effect
42510.0+	0.86516
21255.0+	0.64859
5313.75+	0.33117
42499.8+	0.80689
21255.0+	0.63267
5313.75+	0.31822

6 data points entered.

X-int: 4.03369

Y-int: -4.4970 +/- 0.43966

m: 1.11485 +/- 0.10360

Dm: 10806.7

r: 0.98316

Dose-Effect Curve

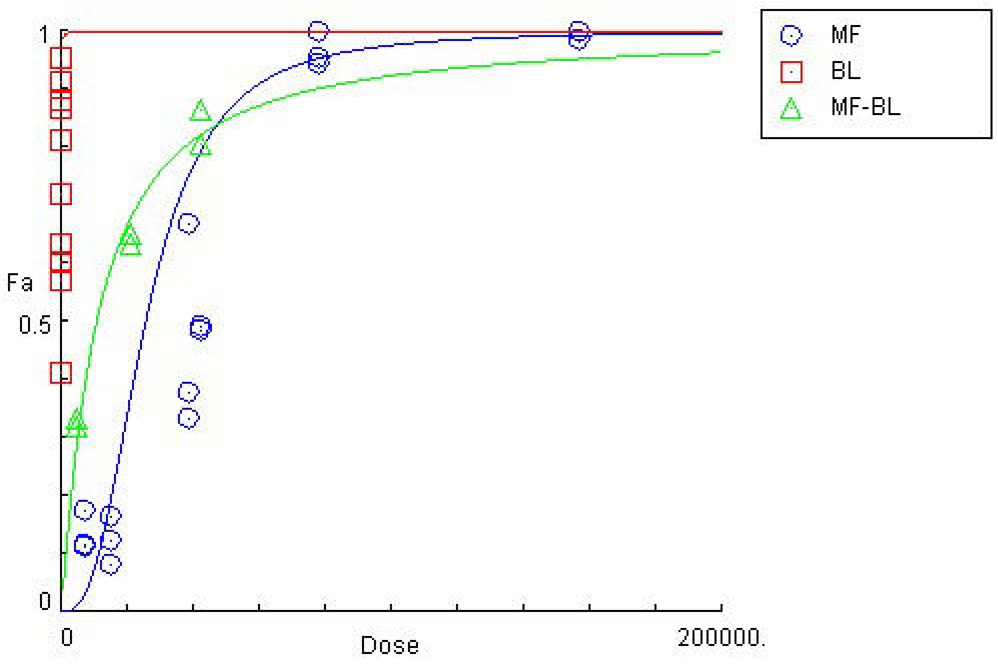

Median-Effect Plot

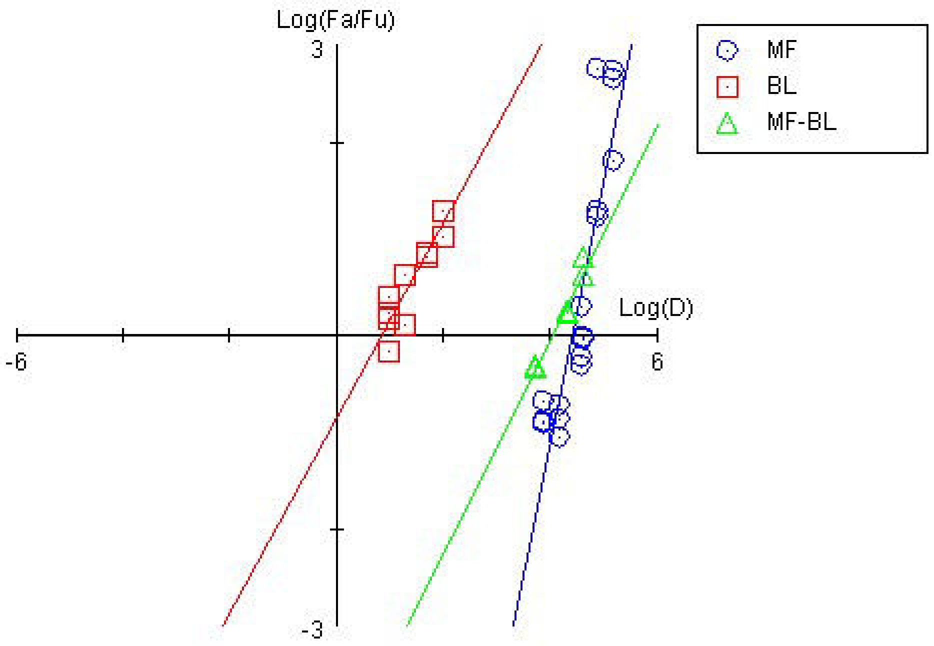

CI Data for Drug Combo: MF-BL (MF+BL [4150.96:1])

Fa	CI Value	S.D.A. Analysis
0.05	0.56515	0.57267 +/- 0.15731
0.1	0.57481	0.57998 +/- 0.12021
0.15	0.59183	0.59585 +/- 0.10018
0.2	0.61173	0.61502 +/- 0.08647
0.25	0.63363	0.63641 +/- 0.07605
0.3	0.65743	0.65983 +/- 0.06770
0.35	0.68334	0.68543 +/- 0.06084
0.4	0.71173	0.71358 +/- 0.05525
0.45	0.74315	0.74480 +/- 0.05090
0.5	0.77836	0.77986 +/- 0.04797
0.55	0.81843	0.81981 +/- 0.04679
0.6	0.86486	0.86617 +/- 0.04786
0.65	0.91993	0.92119 +/- 0.05176
0.7	0.98715	0.98844 +/- 0.05920
0.75	1.07244	1.07382 +/- 0.07123
0.8	1.18663	1.18825 +/- 0.08994
0.85	1.35251	1.35460 +/- 0.12015
0.9	1.62950	1.63266 +/- 0.17568
0.95	2.25690	2.26338 +/- 0.31811
0.97	2.88696	2.89771 +/- 0.47942

CI values for actual experimental points:

Total Dose	Fa	CI Value
42520.2	0.86516	1.05456
21260.1	0.64859	1.04253
5315.03	0.33117	0.62209
42510.0	0.80689	1.31518

Total Dose	Fa	CI Value
21260.1	0.63267	1.08683
5315.03	0.31822	0.64939

Combination Index Plot

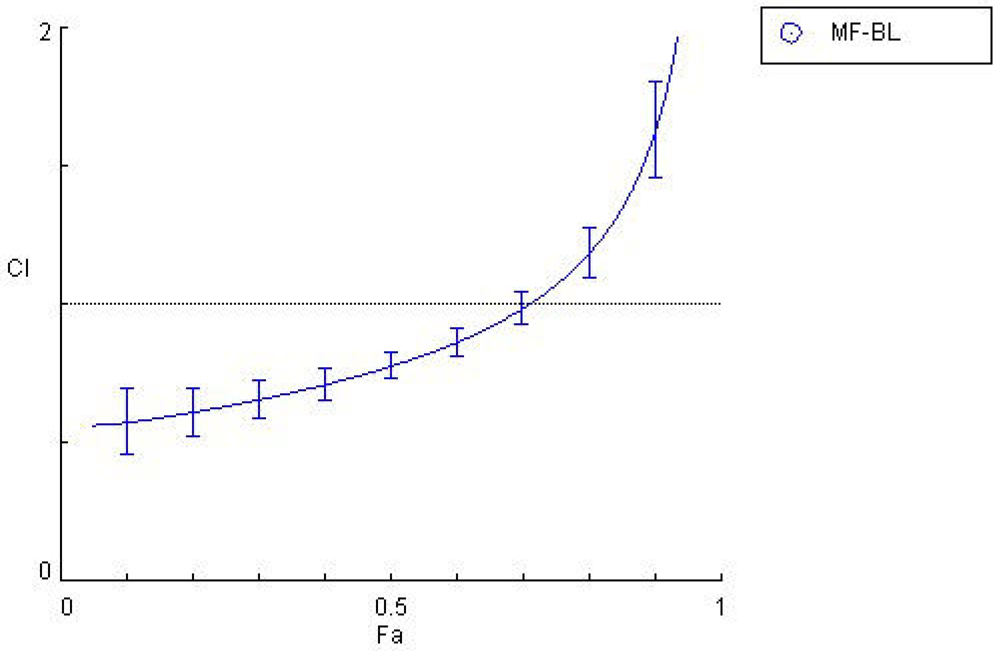

Logarithmic Combination Index Plot

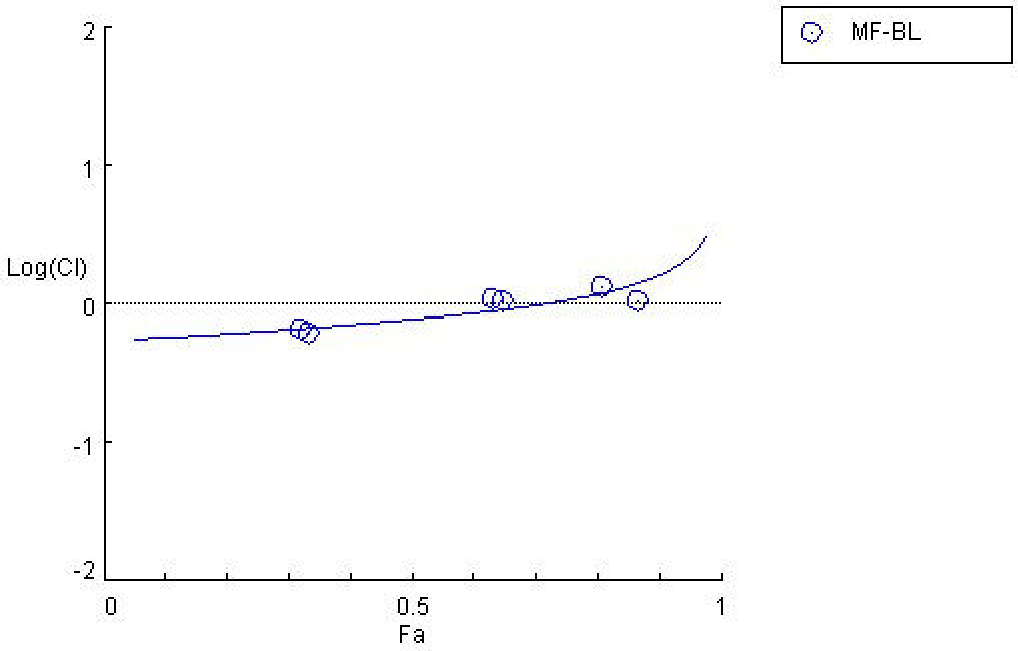

DRI Data for Drug Combo: MF-BL (MF+BL [4150.96:1])

Fa	Dose MF	Dose BL	DRI MF	DRI BL
0.05	8652.15	0.38966	11.2345	2.10024
0.1	11403.4	0.81903	7.57500	2.25838
0.15	13529.2	1.29728	5.93479	2.36219
0.2	15387.5	1.83407	4.93874	2.44350

Fa	Dose MF	Dose BL	DRI MF	DRI BL
0.25	17113.3	2.44130	4.24340	2.51276
0.3	18778.6	3.13419	3.71658	2.57486
0.35	20431.2	3.93256	3.29499	2.63260
0.4	22108.9	4.86279	2.94394	2.68780
0.45	23846.9	5.96081	2.64250	2.74181
0.5	25682.3	7.27686	2.37709	2.79579
0.55	27659.1	8.88348	2.13834	2.85083
0.6	29833.3	10.8894	1.91939	2.90812
0.65	32283.1	13.4652	1.71490	2.96909
0.7	35124.1	16.8952	1.52037	3.03567
0.75	38542.1	21.6903	1.33161	3.11070
0.8	42864.8	28.8717	1.14413	3.19887
0.85	48752.4	40.8181	0.95211	3.30897
0.9	57841.0	64.6531	0.74595	3.46109
0.95	76233.4	135.893	0.50297	3.72169
0.97	92782.0	230.536	0.37997	3.91897

DRI values calculated at experimental points

Fa	Dose MF	Dose BL	DRI MF	DRI BL
0.86516	51043.1	46.1851	1.20073	4.50983
0.64859	32209.3	13.3826	1.51538	2.61353
0.33117	19807.8	3.61801	3.72765	2.82629
0.80689	43561.9	30.1523	1.02499	2.94498
0.63267	31396.8	12.4935	1.47715	2.43990
0.31822	19380.3	3.41175	3.64721	2.66517

DRI Plot for Combo: MF-BL (MF+BL [4150.96:1])

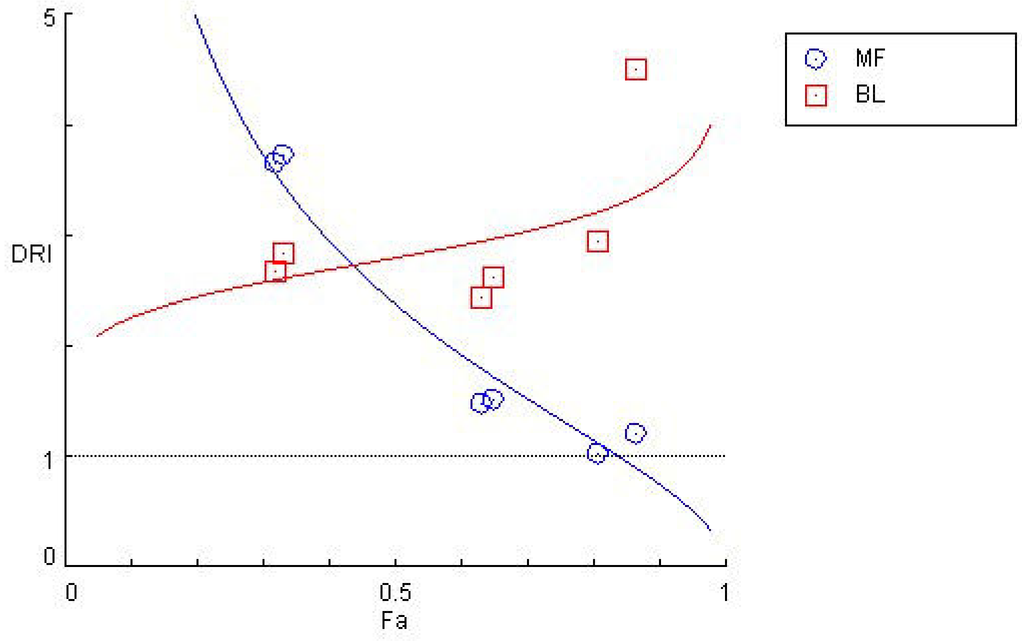

Log(DRI) Plot for Combo: MF-BL (MF+BL [4150.96:1])

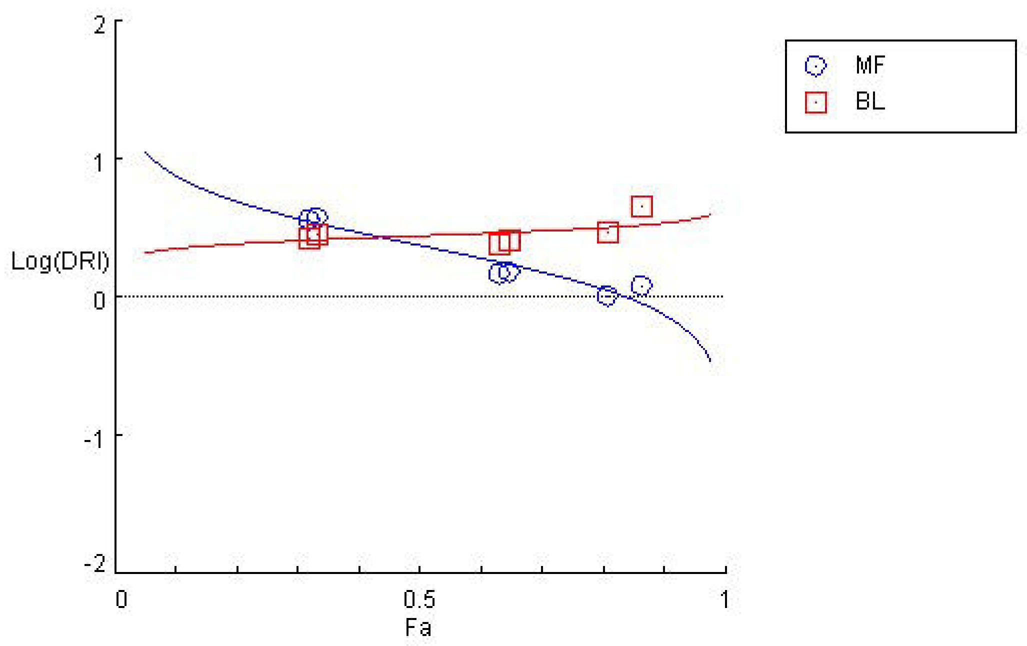

Isobologram for Combo: MF-BL (MF+BL [4150.96:1])

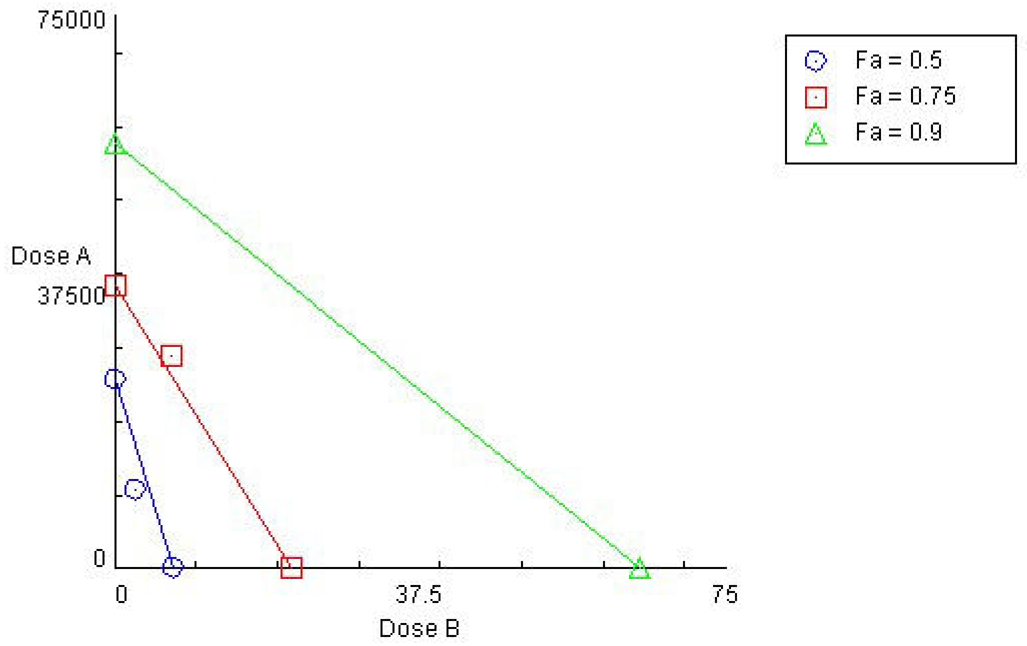

Polygonogram at Fa = 0.9

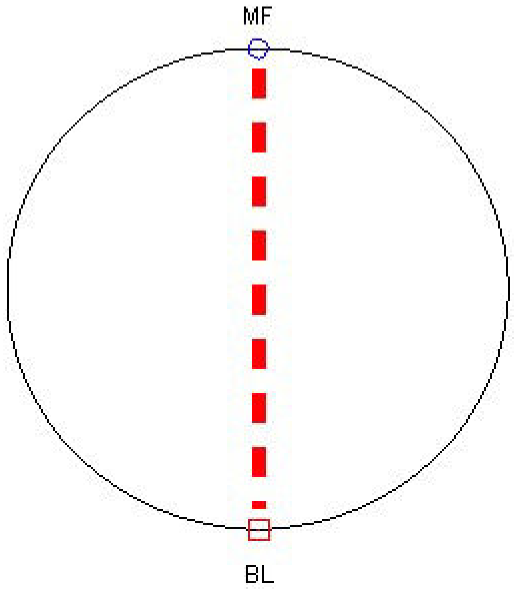
MF
BL

…Data for Drug: DCA [uM]

Dose	Effect
10000.0	0.04902
10000.0	0.16305
10000.0	0.05046
20000.0	0.30530
20000.0	0.44119
20000.0	0.21838
50000.0	0.62232
50000.0	0.77349
50000.0	0.83703
100000.	0.93638
100000.	0.98682
100000.	0.94815
29200.0	0.53133
29200.0	0.50894

14 data points entered.

X-int: 4.45014
Y-int: -10.965 +/- 0.88837
m: 2.46385 +/- 0.19703
Dm: 28193.2
r: 0.96371

Data for Drug: BL [uM]

Dose	Effect
60.5	0.52692
10.0	0.17796
10.0	0.11899
20.0	0.29743
20.0	0.23117
50.0	0.50862
50.0	0.45673
100.0	0.67143
100.0	0.60417
60.5	0.57056

10 data points entered.

X-int: 1.73264
Y-int: -1.7941 +/- 0.11290
m: 1.03546 +/- 0.07069
Dm: 54.0306
r: 0.98186

Data for Drug Combo: DCA-BL (DCA+BL [482.645:1])

Dose A	Effect
29200.0+	0.73375

Dose A	Effect
14600.0+	0.38864
3650.00+	0.15152
29200.0+	0.72727
14600.0+	0.35907
3650.00+	0.06238

6 data points entered.

X-int: 4.23240

Y-int: -6.3481 +/- 0.83332

m: 1.49988 +/- 0.20413

Dm: 17076.5

r: 0.96489

Dose-Effect Curve

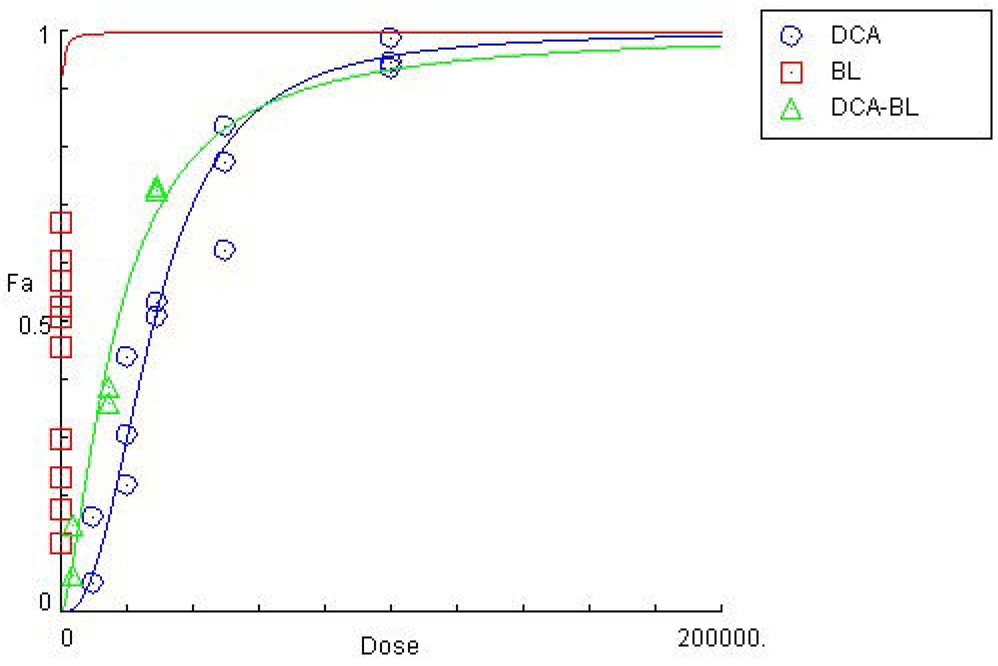

Median-Effect Plot

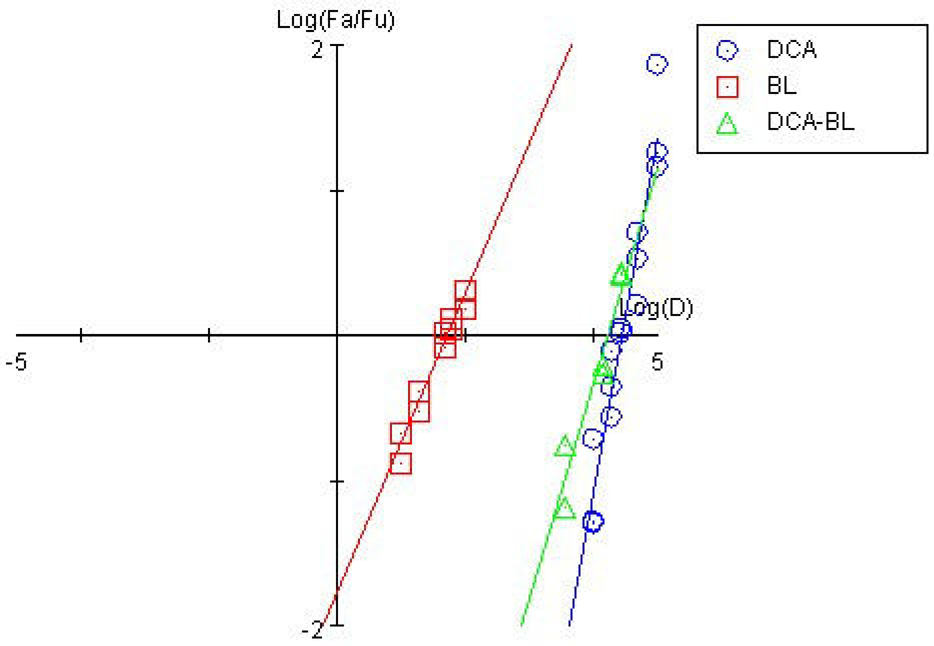

CI Data for Drug Combo: DCA-BL (DCA+BL [482.645:1])

Fa	CI Value	S.D.A. Analysis
0.05	1.85665	1.86309 +/- 0.37057
0.1	1.60137	1.60444 +/- 0.23903
0.15	1.48221	1.48409 +/- 0.17809
0.2	1.41019	1.41151 +/- 0.14033
0.25	1.36147	1.36249 +/- 0.11373
0.3	1.32650	1.32738 +/- 0.09366
0.35	1.30068	1.30151 +/- 0.07808
0.4	1.28150	1.28234 +/- 0.06607
0.45	1.26751	1.26843 +/- 0.05739
0.5	1.25793	1.25896 +/- 0.05224
0.55	1.25235	1.25355 +/- 0.05097
0.6	1.25074	1.25216 +/- 0.05368
0.65	1.25342	1.25512 +/- 0.06013
0.7	1.26117	1.26324 +/- 0.07005
0.75	1.27553	1.27808 +/- 0.08359
0.8	1.29949	1.30269 +/- 0.10166
0.85	1.33932	1.34350 +/- 0.12681
0.9	1.41095	1.41677 +/- 0.16602
0.95	1.57385	1.58343 +/- 0.24648
0.97	1.72786	1.74120 +/- 0.32040

CI values for actual experimental points:

Total Dose	Fa	CI Value
29260.5	0.73375	1.10702
14630.3	0.38864	1.48955
3657.56	0.15152	0.99938
29260.5	0.72727	1.12983

**Total Dose**	**Fa**	**CI Value**
14630.3	0.35907	1.63486
3657.56	0.06238	2.30621

Combination Index Plot

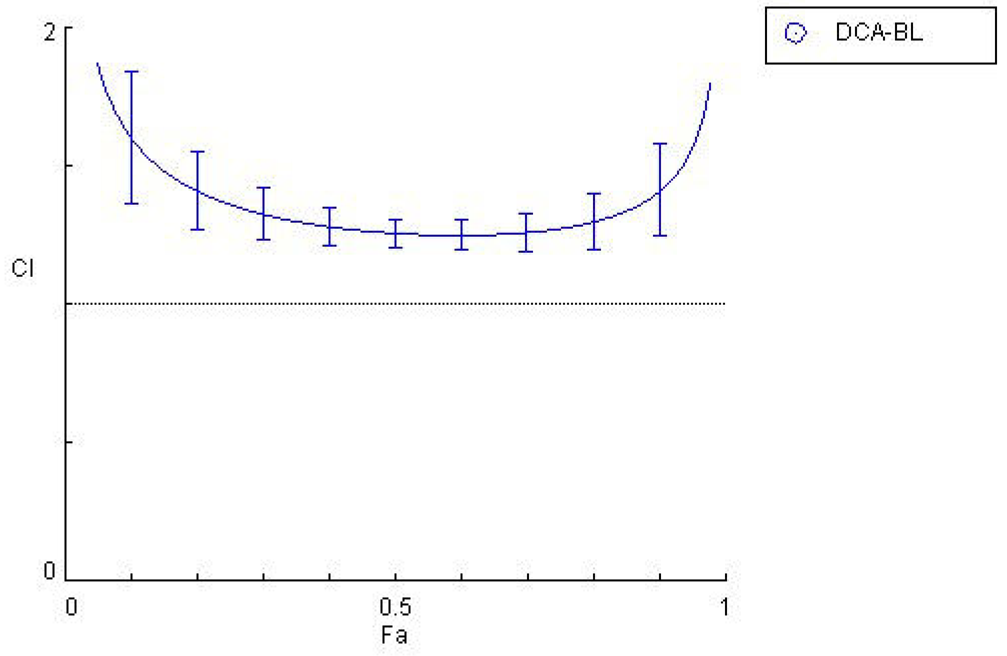

Logarithmic Combination Index Plot

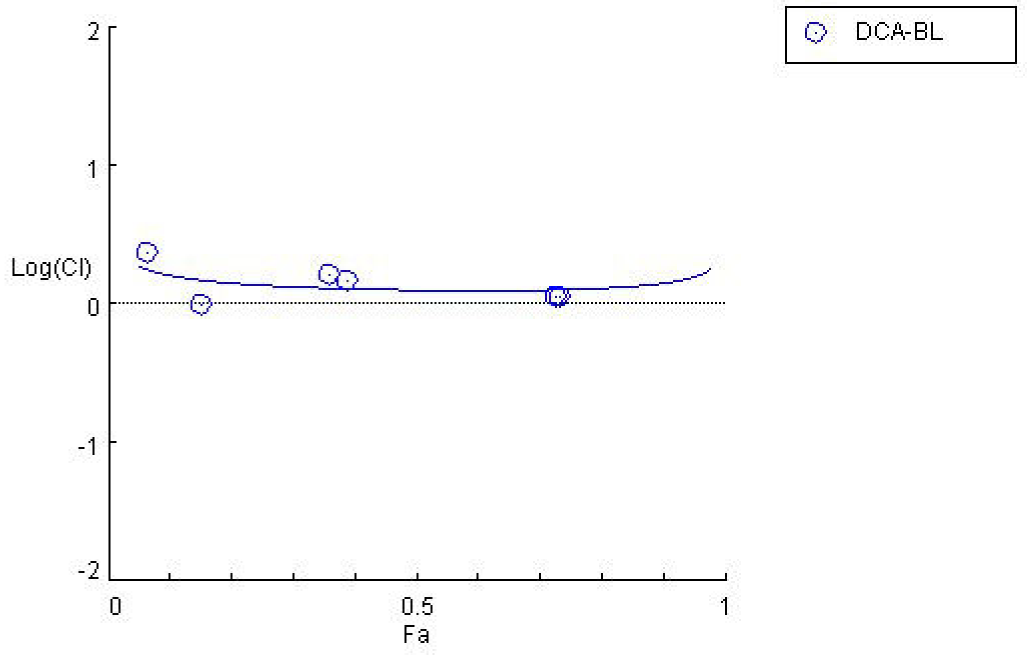

DRI Data for Drug Combo: DCA-BL (DCA+BL [482.645:1])

**Fa**	**Dose DCA**	**Dose BL**	**DRI DCA**	**DRI BL**
0.05	8533.72	3.14541	3.56623	0.63442
0.1	11557.1	6.47256	2.93467	0.79326
0.15	13944.2	10.1184	2.60106	0.91095
0.2	16061.5	14.1644	2.37515	1.01095

Fa	Dose DCA	Dose BL	DRI DCA	DRI BL
0.25	18050.8	18.7007	2.20344	1.10177
0.3	19989.1	23.8377	2.06363	1.18776
0.35	21929.5	29.7167	1.94434	1.27167
0.4	23915.2	36.5240	1.83898	1.35553
0.45	25988.0	44.5117	1.74332	1.44114
0.5	28193.2	54.0306	1.65441	1.53027
0.55	30585.5	65.5851	1.57004	1.62490
0.6	33236.4	79.9283	1.48837	1.72752
0.65	36246.0	98.2377	1.40771	1.84145
0.7	39764.4	122.466	1.32634	1.97153
0.75	44034.4	156.107	1.24218	2.12541
0.8	49488.1	206.102	1.15238	2.31634
0.85	57002.7	288.515	1.05229	2.57062
0.9	68776.6	451.028	0.93267	2.95201
0.95	93142.8	928.114	0.76750	3.69112
0.97	115575.	1550.93	0.66811	4.32717

DRI values calculated at experimental points

Fa	Dose DCA	Dose BL	DRI DCA	DRI BL
0.73375	42543.3	143.821	1.45696	2.37720
0.38864	23457.9	34.8841	1.60670	1.15319
0.15152	14011.5	10.2350	3.83877	1.35340
0.72727	41978.9	139.322	1.43763	2.30285
0.35907	22285.2	30.8766	1.52638	1.02071
0.06238	9385.30	3.94433	2.57132	0.52156

DRI Plot for Combo: DCA-BL (DCA+BL [482.645:1])

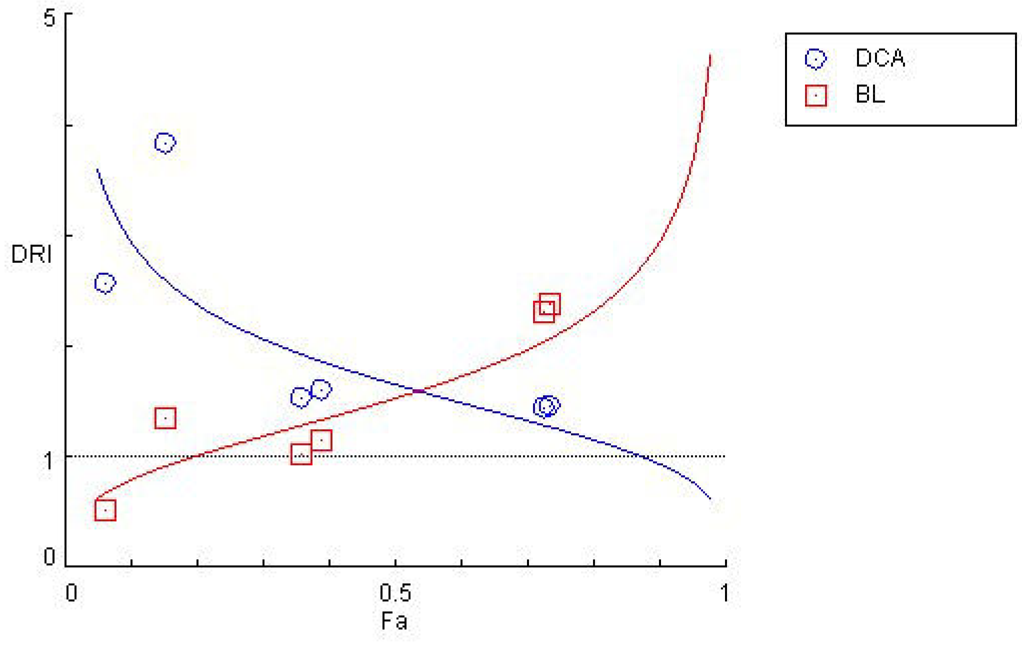

Log(DRI) Plot for Combo: DCA-BL (DCA+BL [482.645:1])

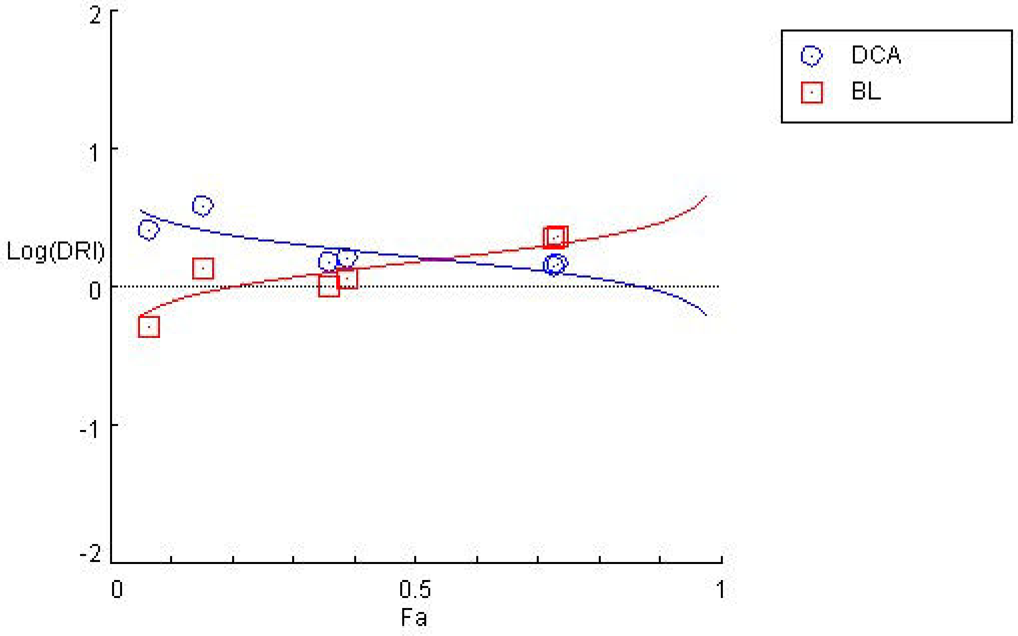

Isobologram for Combo: DCA-BL (DCA+BL [482.645:1])

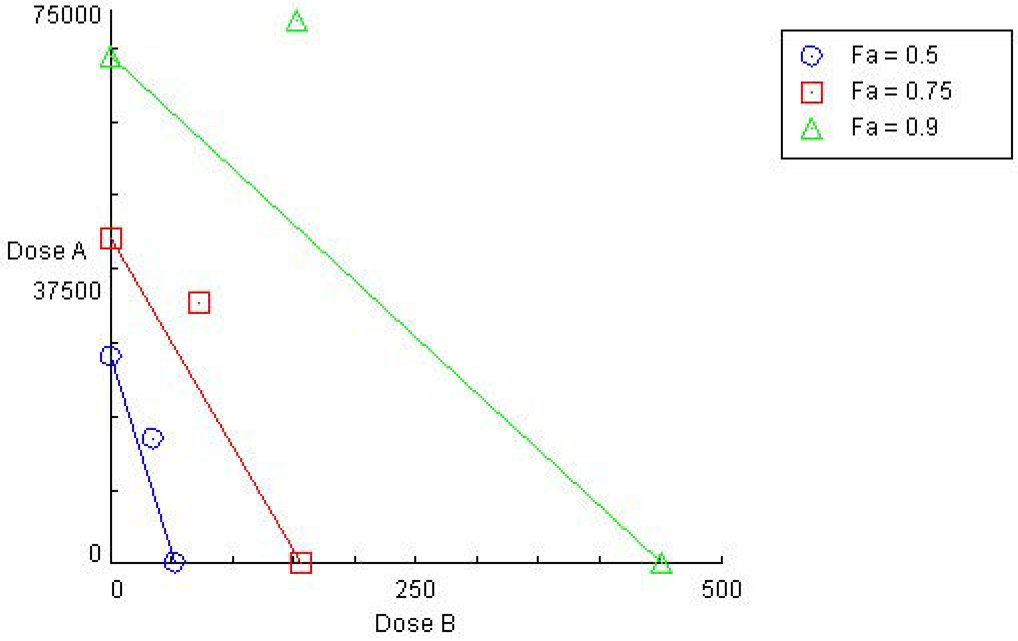

Polygonogram at Fa = 0.9

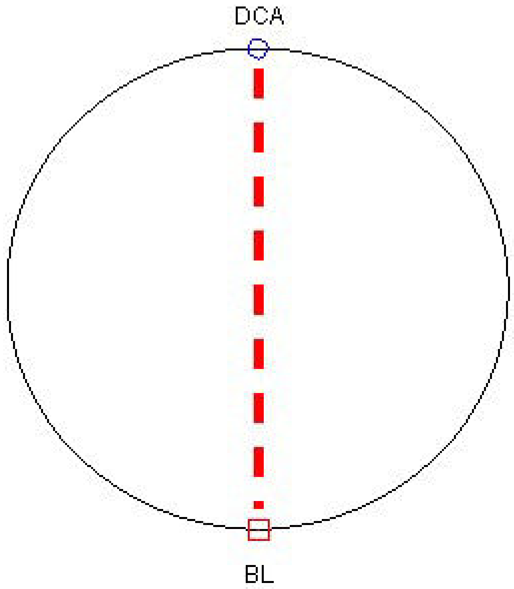

DCA
BL

…Data for Drug: DCA [uM]

Dose	Effect
10000.0	0.01216
10000.0	0.08649
10000.0	0.01
20000.0	0.17153
20000.0	0.08551
20000.0	0.03562
50000.0	0.71466
50000.0	0.69233
50000.0	0.45257
100000.	0.96291
100000.	0.93367
100000.	0.88499
40613.0	0.39533
40613.0	0.54637

14 data points entered.

X-int: 4.61751
Y-int: -13.134 +/- 1.12148
m: 2.84432 +/- 0.24760
Dm: 41448.5
r: 0.95742

Data for Drug: BL [uM]

Dose	Effect
50.0	0.47634
50.0	0.54304
100.0	0.60608
100.0	0.64751
200.0	0.66695
200.0	0.70512
55.09	0.60225
55.09	0.64755

8 data points entered.

X-int: 1.42816
Y-int: -0.5666 +/- 0.26652
m: 0.39676 +/- 0.13666
Dm: 26.8018
r: 0.76430

Data for Drug Combo: DCA-BL (DCA+BL [737.21:1])

Dose A	Effect
40612.9+	0.74799
20306.5+	0.33011
5076.61+	0.11433

Dose A	Effect
40612.9+	0.86172
20306.5+	0.63912
5076.61+	0.19056

6 data points entered.

X-int: 4.24238

Y-int: -6.3396 +/- 1.23943

m: 1.49434 +/- 0.29338

Dm: 17473.4

r: 0.93081

Dose-Effect Curve

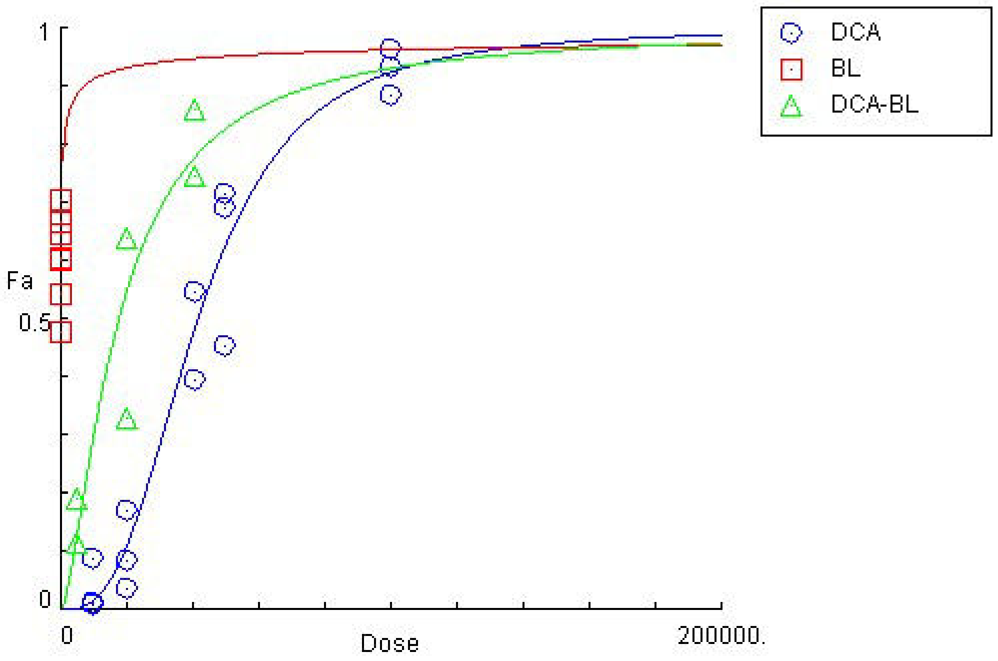

Median-Effect Plot

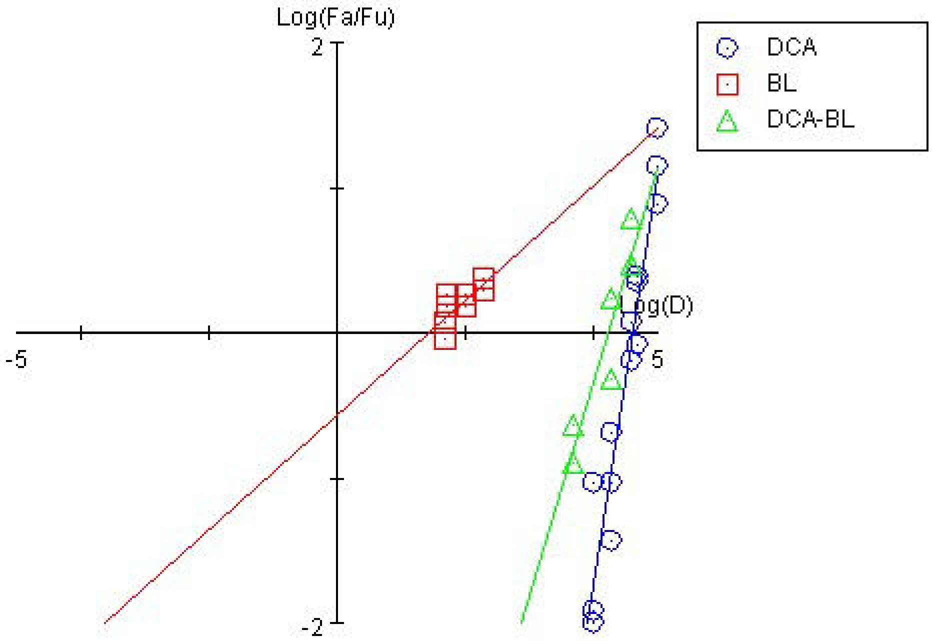

CI Data for Drug Combo: DCA-BL (DCA+BL [737.21:1])

Fa	CI Value	S.D.A. Analysis
0.05	205.886	351.369 +/- 1550.60
0.1	51.7963	69.8908 +/- 198.035
0.15	22.1507	26.9666 +/- 54.5052
0.2	11.7678	13.4981 +/- 20.3489
0.25	7.04671	7.77134 +/- 8.89349
0.3	4.56040	4.89081 +/- 4.25063
0.35	3.12379	3.28150 +/- 2.13598
0.4	2.24087	2.31760 +/- 1.09824
0.45	1.67546	1.71273 +/- 0.56550
0.5	1.30414	1.32195 +/- 0.28737
0.55	1.05782	1.06617 +/- 0.14679
0.6	0.89578	0.89980 +/- 0.08738
0.65	0.79323	0.79555 +/- 0.07355
0.7	0.73501	0.73695 +/- 0.07541
0.75	0.71234	0.71456 +/- 0.08116
0.8	0.72173	0.72457 +/- 0.09084
0.85	0.76598	0.76971 +/- 0.10827
0.9	0.86110	0.86624 +/- 0.14163
0.95	1.07636	1.08482 +/- 0.22136
0.97	1.27130	1.28333 +/- 0.30106

CI values for actual experimental points:

Total Dose	Fa	CI Value
40668.0	0.74799	0.80087
20334.0	0.33011	6.74493
5083.5	0.11433	44.9958
40668.0	0.86172	0.53540
20334.0	0.63912	0.64409
5083.5	0.19056	10.0447

Combination Index Plot

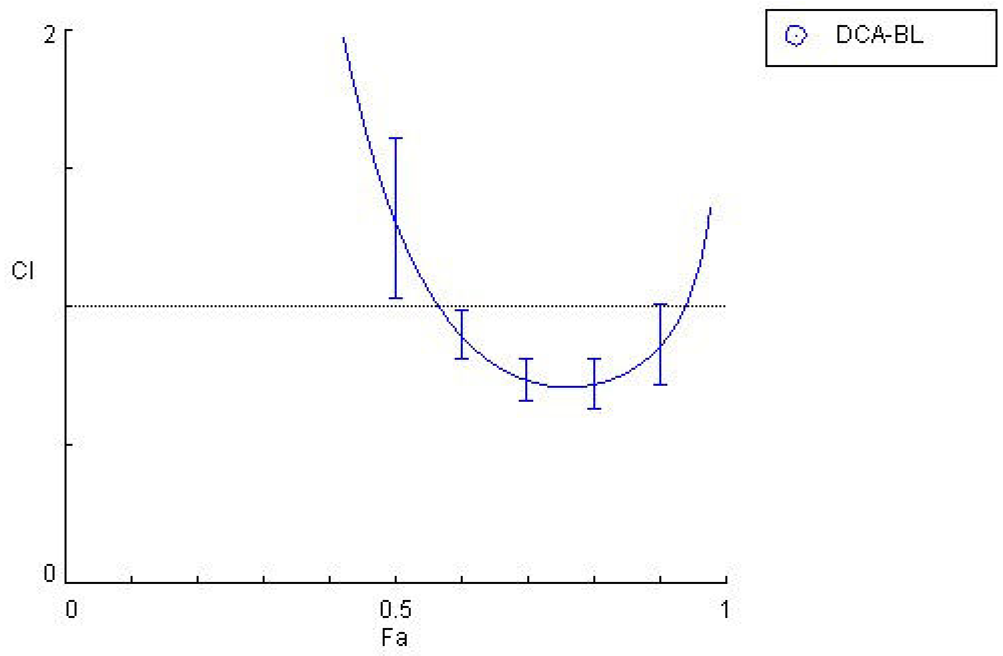

Logarithmic Combination Index Plot

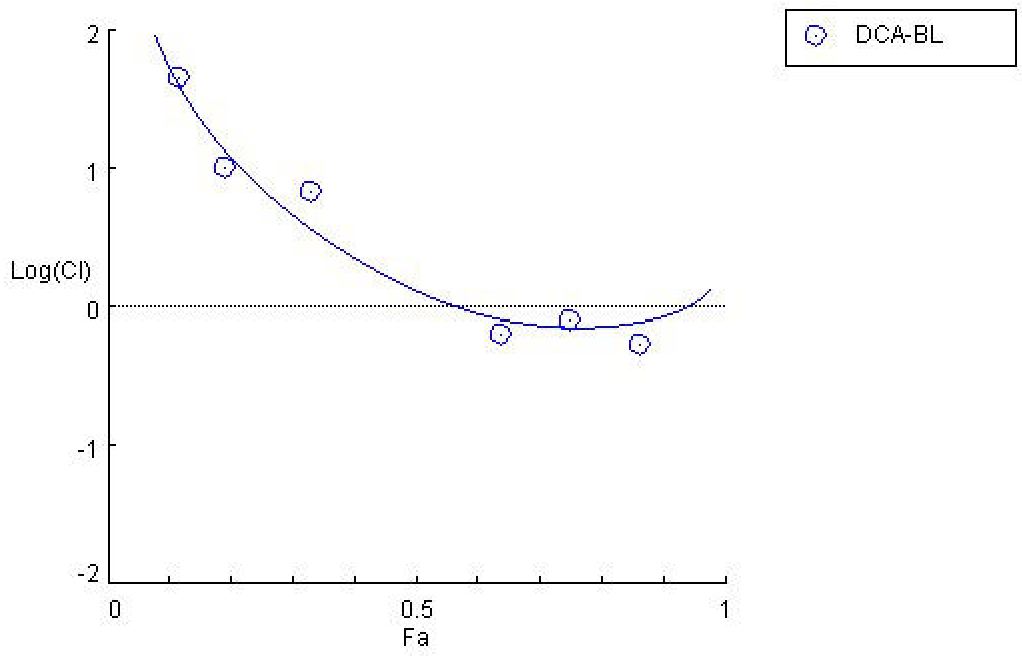

DRI Data for Drug Combo: DCA-BL (DCA+BL [737.21:1])

Fa	Dose DCA	Dose BL	DRI DCA	DRI BL
0.05	14720.6	0.01604	6.05155	0.00486
0.1	19143.3	0.10546	4.77308	0.01938
0.15	22524.5	0.33844	4.12084	0.04565
0.2	25458.8	0.81420	3.68928	0.08698
0.25	28168.5	1.68123	3.36713	0.14815
0.3	30770.6	3.16749	3.10881	0.23592
0.35	33341.8	5.63075	2.89140	0.35998
0.4	35941.7	9.64592	2.70177	0.53455
0.45	38625.0	16.1626	2.53163	0.78097

Fa	Dose DCA	Dose BL	DRI DCA	DRI BL
0.5	41448.5	26.8018	2.37531	1.13232
0.55	44478.3	44.4445	2.22864	1.64173
0.6	47798.9	74.4707	2.08829	2.39855
0.65	51526.3	127.574	1.95133	3.56170
0.7	55831.7	226.785	1.81487	5.43464
0.75	60989.3	427.270	1.67564	8.65406
0.8	67480.7	882.263	1.52932	14.7404
0.85	76271.4	2122.53	1.36916	28.0890
0.9	89742.7	6811.45	1.18207	66.1413
0.95	116705.	44785.5	0.93234	263.762
0.97	140692.	171037.	0.78748	705.751

DRI values calculated at experimental points

Fa	Dose DCA	Dose BL	DRI DCA	DRI BL
0.74799	60760.5	415.910	1.49609	7.54964
0.33011	32318.6	4.50331	1.59154	0.16349
0.11433	20179.8	0.15390	3.97505	0.02235
0.86172	78863.3	2697.08	1.94183	48.9577
0.63912	50673.3	113.186	2.49543	4.10913
0.19056	24926.4	0.69975	4.91005	0.10162

DRI Plot for Combo: DCA-BL (DCA+BL [737.21:1])

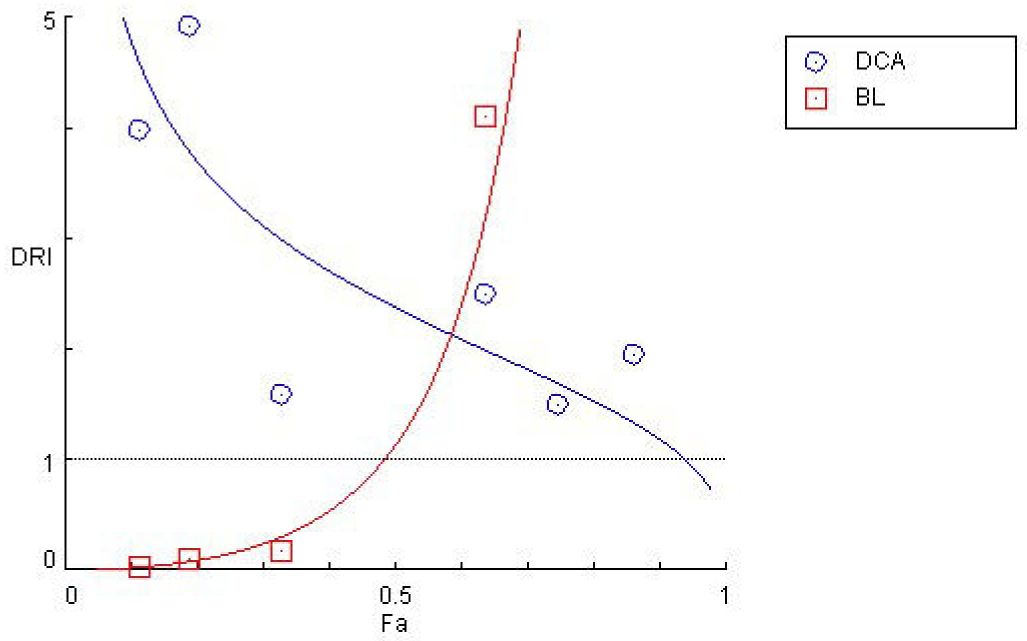

Log(DRI) Plot for Combo: DCA-BL (DCA+BL [737.21:1])

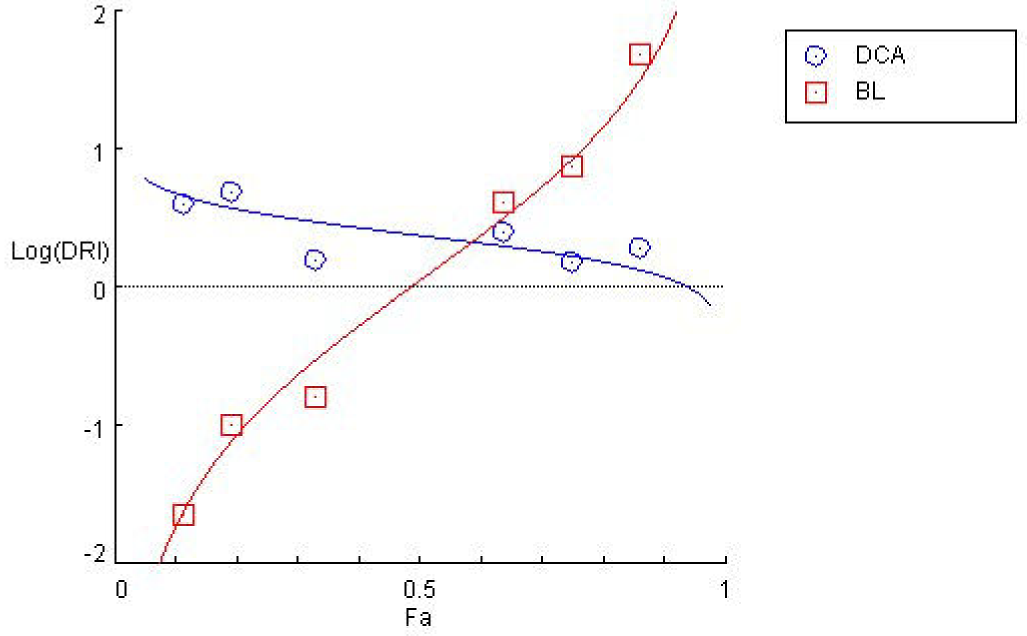

Isobologram for Combo: DCA-BL (DCA+BL [737.21:1])

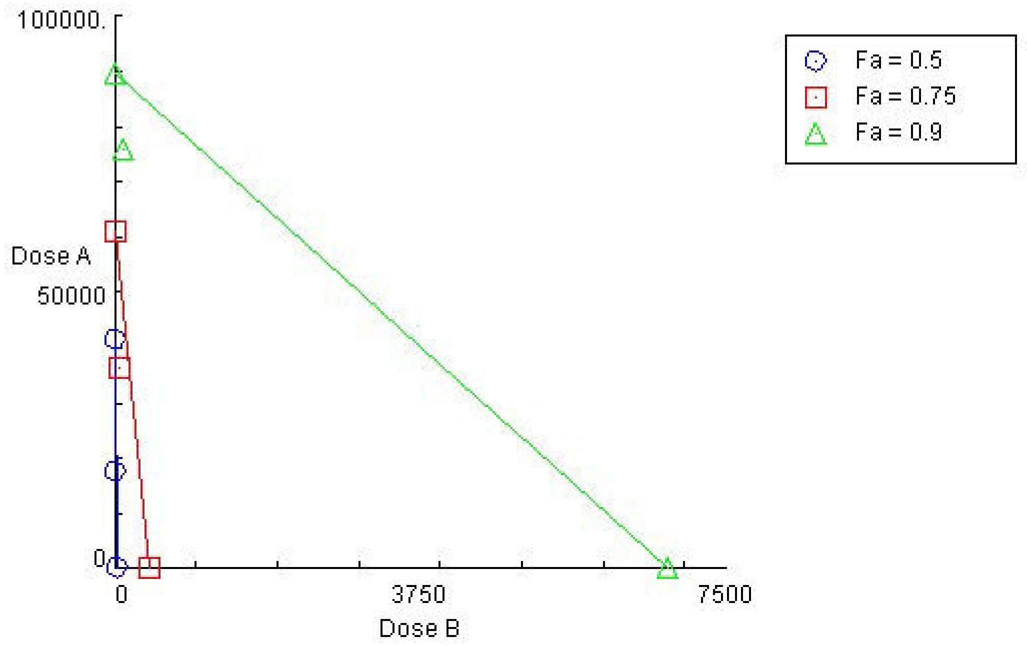

Polygonogram at Fa = 0.9

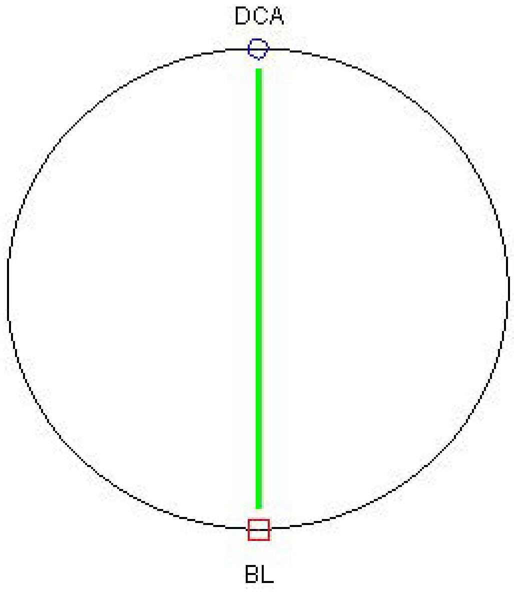
DCA
BL

…Data for Drug: DCA [uM]

Dose	Effect
10000.0	0.29654
10000.0	0.38091
10000.0	0.64051
20000.0	0.51670
20000.0	0.54896
20000.0	0.74960
50000.0	0.87964
50000.0	0.88269
50000.0	0.93805
100000.	0.99
100000.	0.98576
100000.	0.98371
13516.5	0.32942
13516.5	0.2739

14 data points entered.

X-int: 4.17281
Y-int: -8.7537 +/- 0.86611
m: 2.09779 +/- 0.19406
Dm: 14887.0
r: 0.95230

Data for Drug: BL [uM]

Dose	Effect
50.0	0.68241
50.0	0.29706
100.0	0.85466
100.0	0.59909
200.0	0.94684
200.0	0.79812
11.8	0.50912
11.8	0.26492

8 data points entered.

X-int: 1.44869
Y-int: -1.3171 +/- 0.56419
m: 0.90918 +/- 0.30906
Dm: 28.0991
r: 0.76848

Data for Drug Combo: DCA-BL (DCA+BL [1145.46:1])

Dose A	Effect
13516.5+	0.44489
6758.25+	0.28218
1689.58+	0.05124

Dose A	Effect
13516.5+	0.35268
6758.25+	0.20802
1689.58+	0.02304

6 data points entered.

X-int: 4.22430

Y-int: -6.0390 +/- 0.67453

m: 1.42959 +/- 0.17994

Dm: 16761.2

r: 0.96975

Dose-Effect Curve

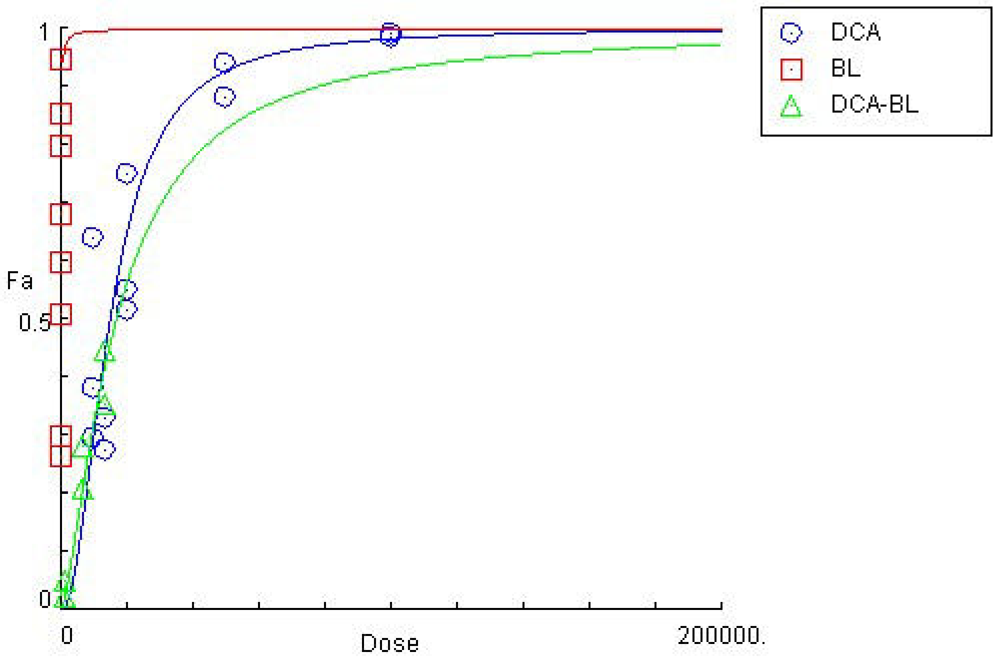

Median-Effect Plot

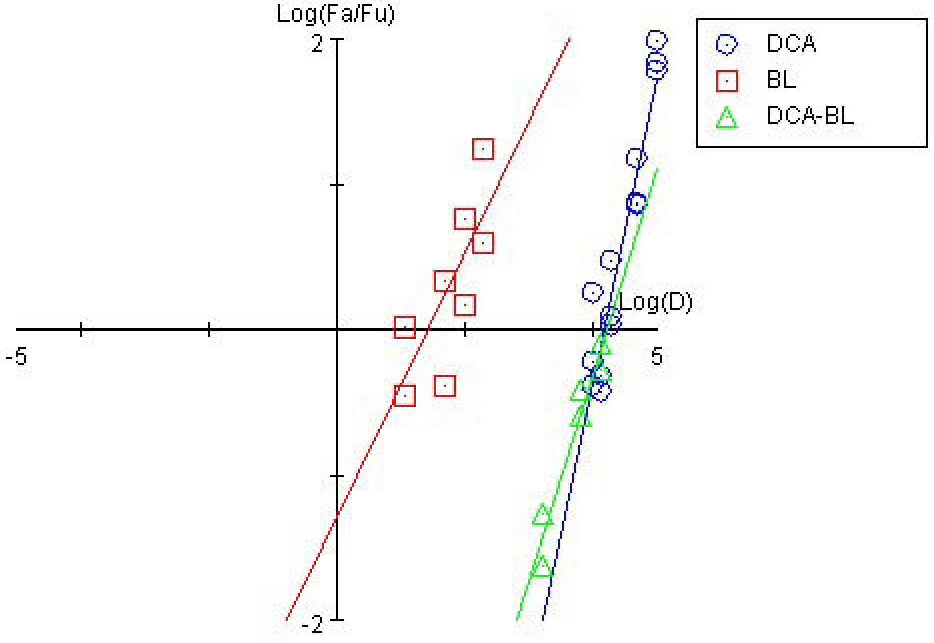

CI Data for Drug Combo: DCA-BL (DCA+BL [1145.46:1])

Fa	CI Value	S.D.A. Analysis
0.05	2.27514	2.36359 +/- 1.16726
0.1	1.94352	1.98382 +/- 0.61072
0.15	1.80634	1.82998 +/- 0.40343
0.2	1.73237	1.74777 +/- 0.29405
0.25	1.68844	1.69909 +/- 0.22767
0.3	1.66183	1.66952 +/- 0.18531
0.35	1.64663	1.65242 +/- 0.15889
0.4	1.63975	1.64432 +/- 0.14436
0.45	1.63955	1.64339 +/- 0.13925
0.5	1.64521	1.64868 +/- 0.14165
0.55	1.65648	1.65989 +/- 0.15004
0.6	1.67361	1.67723 +/- 0.16339
0.65	1.69736	1.70148 +/- 0.18134
0.7	1.72927	1.73418 +/- 0.20425
0.75	1.77204	1.77814 +/- 0.23342
0.8	1.83069	1.83853 +/- 0.27180
0.85	1.91545	1.92591 +/- 0.32578
0.9	2.05129	2.06619 +/- 0.41192
0.95	2.32795	2.35271 +/- 0.59380
0.97	2.56990	2.60422 +/- 0.76372

CI values for actual experimental points:

Total Dose	Fa	CI Value
13528.3	0.44489	1.54467
6764.15	0.28218	1.29482
1691.06	0.05124	1.75734
13528.3	0.35268	2.03173
6764.15	0.20802	1.77228
1691.06	0.02304	3.91434

Combination Index Plot

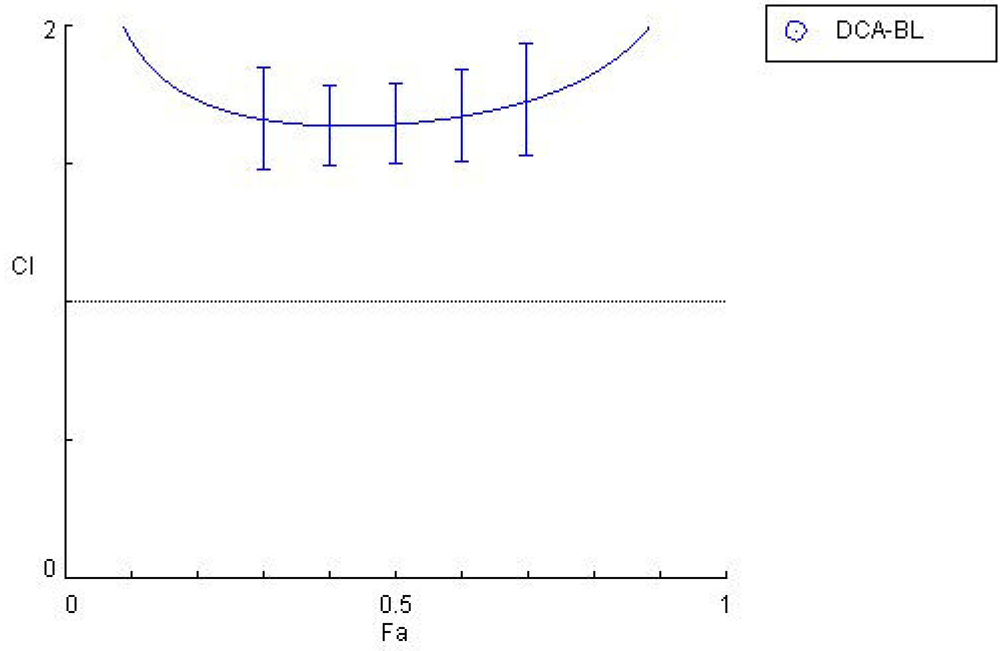

Logarithmic Combination Index Plot

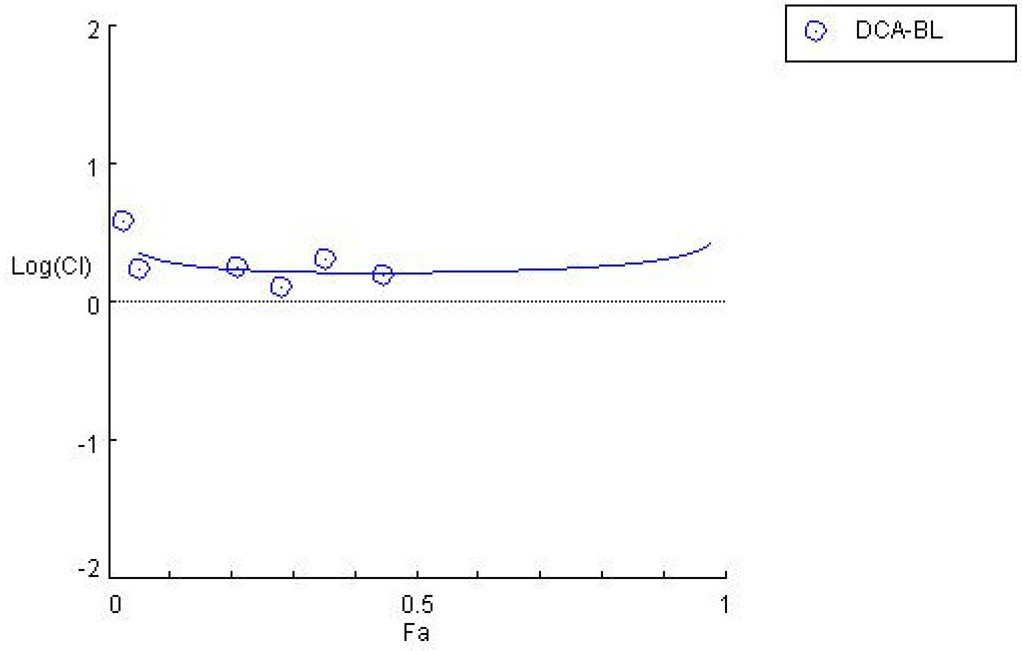

DRI Data for Drug Combo: DCA-BL (DCA+BL [1145.46:1])

Fa	Dose DCA	Dose BL	DRI DCA	DRI BL
0.05	3657.92	1.10205	1.71317	0.59122
0.1	5223.08	2.50684	1.45043	0.79740
0.15	6511.80	4.16977	1.30837	0.95967
0.2	7687.93	6.11633	1.21067	1.10329
0.25	8817.93	8.39286	1.13550	1.23798
0.3	9940.20	11.0651	1.07367	1.36903
0.35	11082.8	14.2230	1.02043	1.50004
0.4	12270.6	17.9892	0.97301	1.63396
0.45	13528.9	22.5339	0.92961	1.77359

Fa	Dose DCA	Dose BL	DRI DCA	DRI BL
0.5	14887.0	28.0991	0.88896	1.92197
0.55	16381.4	35.0388	0.85009	2.08277
0.6	18061.3	43.8909	0.81217	2.26075
0.65	19996.9	55.5129	0.77442	2.46258
0.7	22295.6	71.3556	0.73602	2.69824
0.75	25133.1	94.0753	0.69594	2.98388
0.8	28827.3	129.091	0.65273	3.34816
0.85	34033.9	189.353	0.60399	3.84921
0.9	42431.3	314.962	0.54483	4.63251
0.95	60586.9	716.450	0.46127	6.24809
0.97	78063.0	1285.72	0.40974	7.73029

DRI values calculated at experimental points

Fa	Dose DCA	Dose BL	DRI DCA	DRI BL
0.44489	13396.3	22.0274	0.99110	1.86672
0.28218	9539.22	10.0623	1.41149	1.70547
0.05124	3703.09	1.13370	2.19172	0.76860
0.35268	11145.2	14.4084	0.82456	1.22105
0.20802	7871.01	6.45765	1.16465	1.09451
0.02304	2494.62	0.45567	1.47647	0.30892

DRI Plot for Combo: DCA-BL (DCA+BL [1145.46:1])

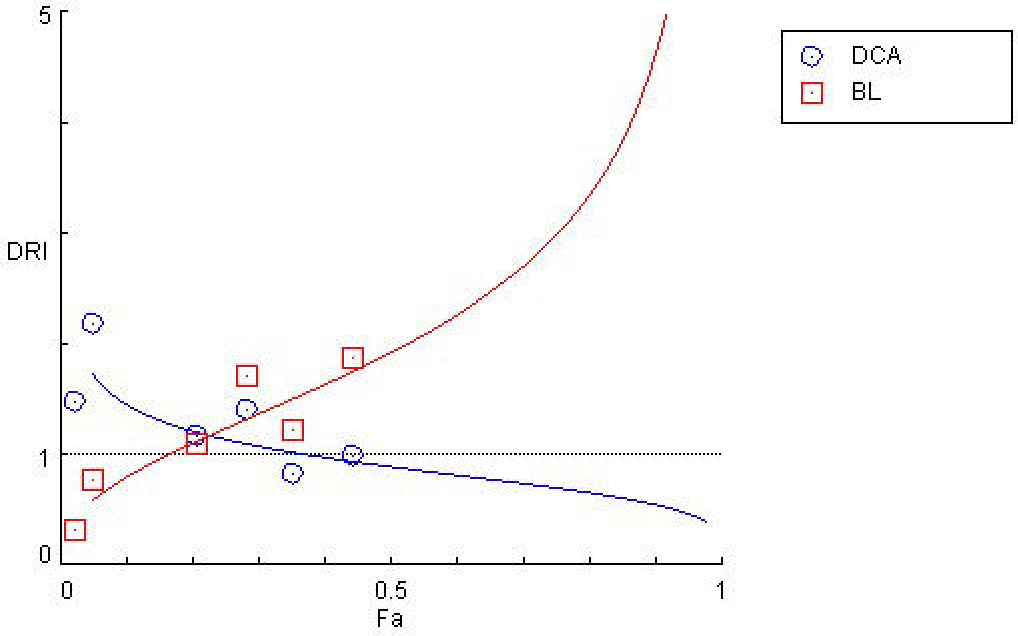

Log(DRI) Plot for Combo: DCA-BL (DCA+BL [1145.46:1])

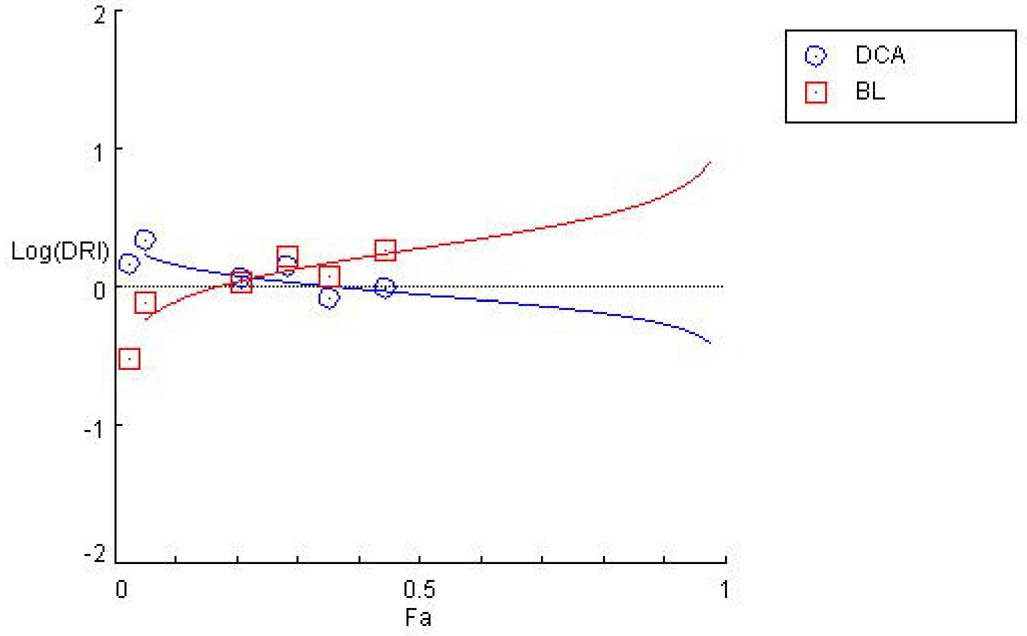

Isobologram for Combo: DCA-BL (DCA+BL [1145.46:1])

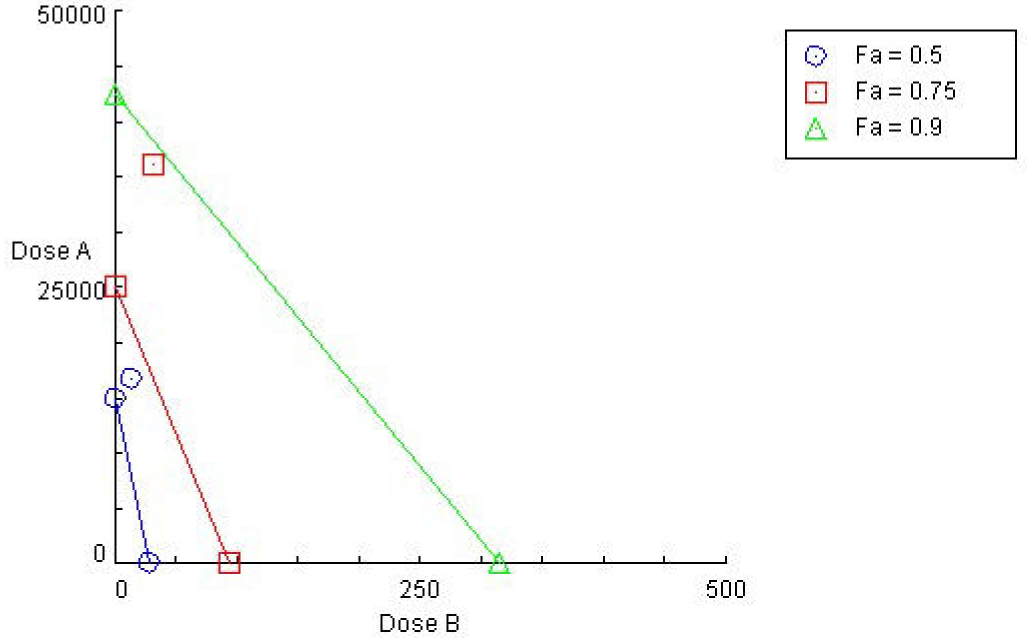

Polygonogram at Fa = 0.9

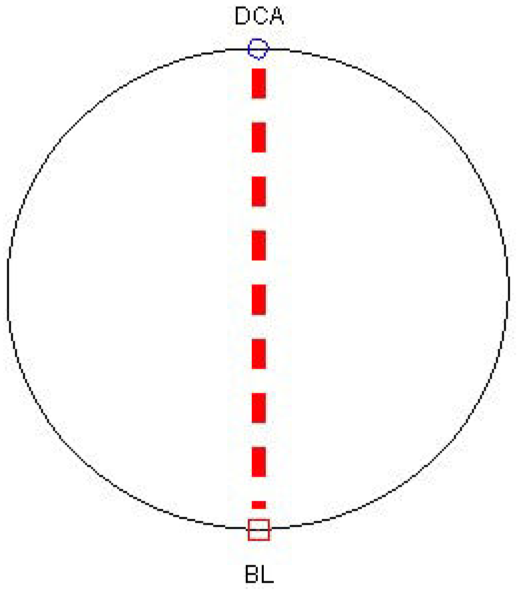
DCA
BL

…Data for Drug: DCA [uM]

Dose	Effect
10000.0	0.22502
10000.0	0.01
10000.0	0.01
20000.0	0.23092
20000.0	0.21073
20000.0	0.25615
50000.0	0.87071
50000.0	0.86300
50000.0	0.89430
100000.	0.98360
100000.	0.98621
100000.	0.97747
27096.5	0.53809
27096.5	0.55887

14 data points entered.

X-int: 4.44890
Y-int: -14.593 +/- 1.21120
m: 3.28009 +/- 0.26890
Dm: 28112.5
r: 0.96196

Data for Drug: BL [uM]

Dose	Effect
10.0	0.63176
10.0	0.41297
20.0	0.81331
20.0	0.56917
50.0	0.86886
50.0	0.88044
100.0	0.91241
100.0	0.95258
10.241	0.61994
10.241	0.65059

10 data points entered.

X-int: 0.88493
Y-int: -0.9126 +/- 0.22509
m: 1.03128 +/- 0.15459
Dm: 7.67233
r: 0.92066

Data for Drug Combo: DCA-BL (DCA+BL [2645.88:1])

Dose A	Effect
27096.5+	0.66694

Dose A	Effect
13548.3+	0.50932
3387.06+	0.27877
27096.5+	0.69409
13548.3+	0.41939
3387.06+	0.27234

6 data points entered.

X-int: 4.09612

Y-int: -3.2573 +/- 0.43915

m: 0.79521 +/- 0.10846

Dm: 12477.3

r: 0.96475

Dose-Effect Curve

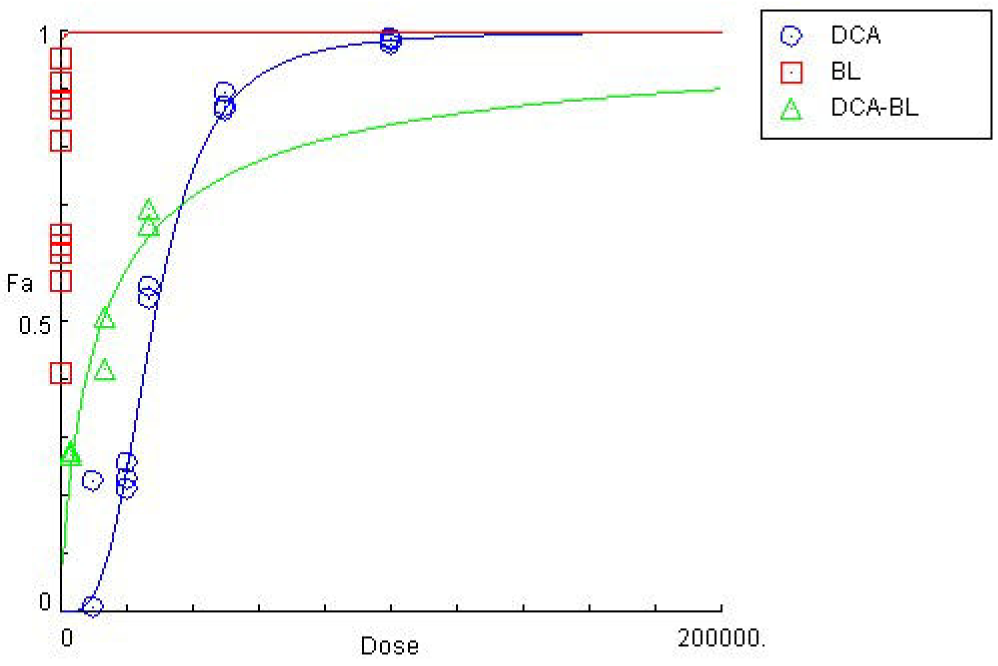

Median-Effect Plot

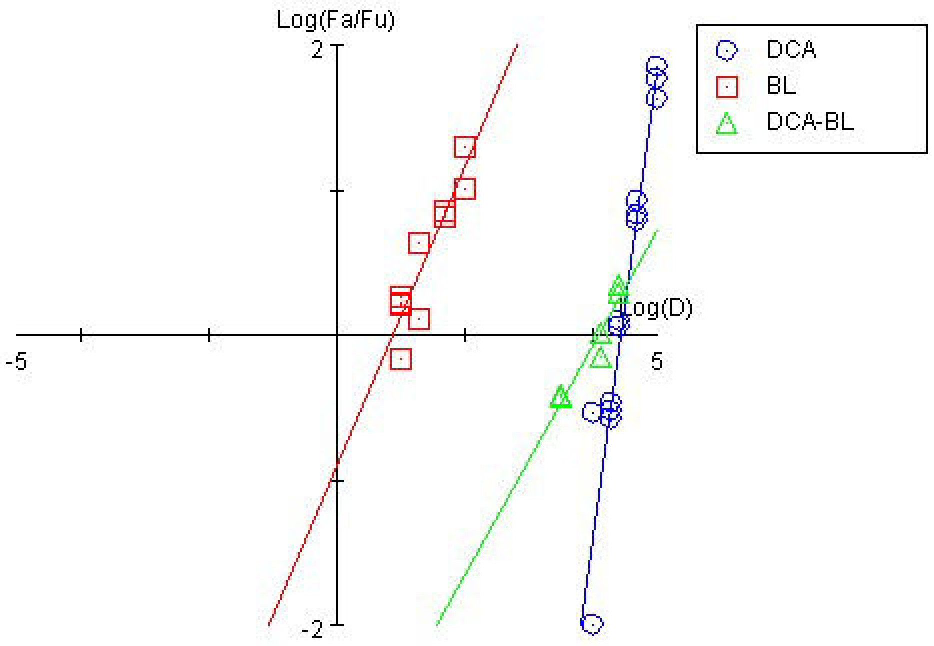

CI Data for Drug Combo: DCA-BL (DCA+BL [2645.88:1])

Fa	CI Value	S.D.A. Analysis
0.05	0.29008	0.29393 +/- 0.08218
0.1	0.38111	0.38465 +/- 0.08268
0.15	0.45790	0.46125 +/- 0.08144
0.2	0.53067	0.53389 +/- 0.07964
0.25	0.60361	0.60674 +/- 0.07770
0.3	0.67935	0.68244 +/- 0.07594
0.35	0.76012	0.76321 +/- 0.07471
0.4	0.84823	0.85137 +/- 0.07451
0.45	0.94639	0.94963 +/- 0.07606
0.5	1.05807	1.06148 +/- 0.08039
0.55	1.18808	1.19175 +/- 0.08882
0.6	1.34332	1.34738 +/- 0.10308
0.65	1.53442	1.53906 +/- 0.12549
0.7	1.77865	1.78416 +/- 0.15966
0.75	2.10650	2.11340 +/- 0.21215
0.8	2.57768	2.58689 +/- 0.29654
0.85	3.32824	3.34180 +/- 0.44595
0.9	4.75507	4.77859 +/- 0.76501
0.95	8.76699	8.82660 +/- 1.81792
0.97	13.8399	13.9565 +/- 3.35713

CI values for actual experimental points:

Total Dose	Fa	CI Value
27106.7	0.66694	1.46073
13553.4	0.50932	1.12019
3388.34	0.27877	0.58039
27106.7	0.69409	1.35391

Total Dose	Fa	CI Value
13553.4	0.41939	1.44708
3388.34	0.27234	0.59528

Combination Index Plot

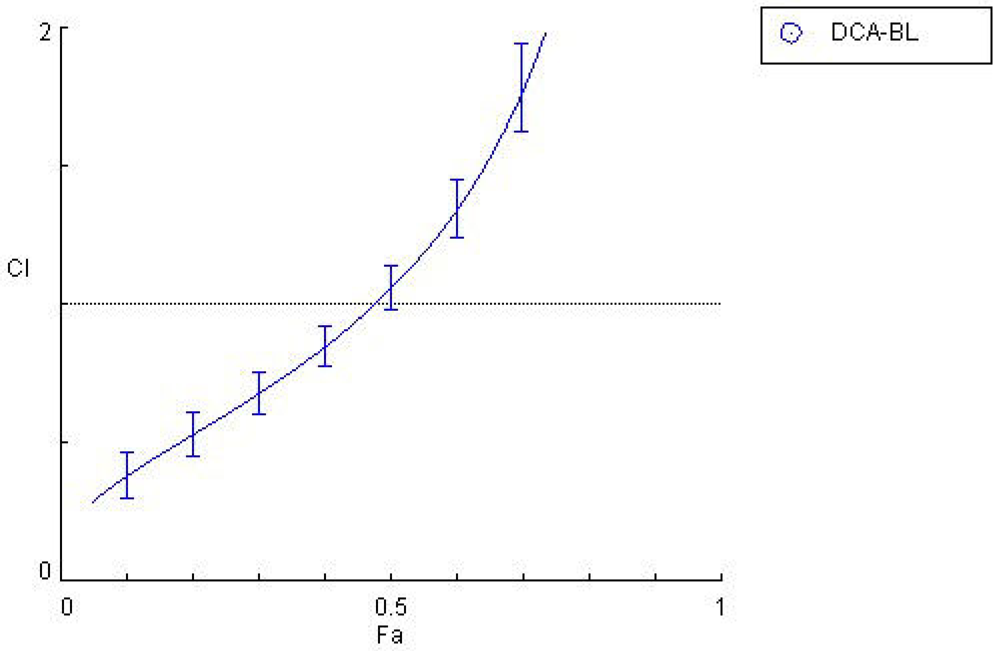

Logarithmic Combination Index Plot

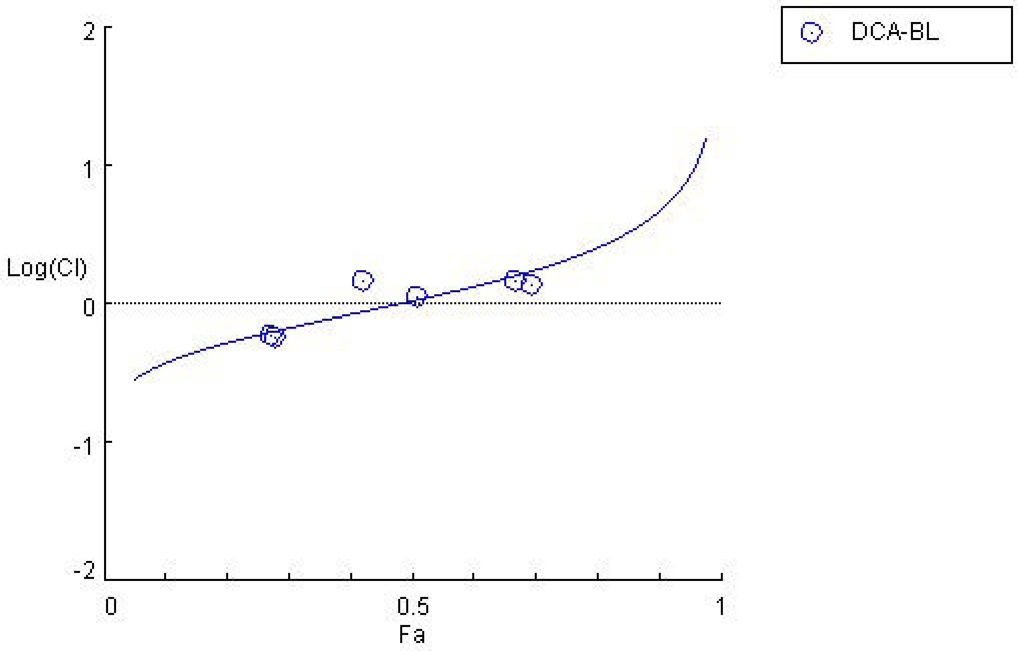

DRI Data for Drug Combo: DCA-BL (DCA+BL [2645.88:1])

Fa	Dose DCA	Dose BL	DRI DCA	DRI BL
0.05	11456.3	0.44153	37.2534	3.79887
0.1	14387.3	0.91124	18.2817	3.06365
0.15	16566.6	1.42709	11.7655	2.68165
0.2	18422.5	2.00046	8.44316	2.42581

Fa	Dose DCA	Dose BL	DRI DCA	DRI BL
0.25	20111.3	2.64411	6.41919	2.23301
0.3	21712.7	3.37375	5.05245	2.07717
0.35	23277.5	4.20957	4.06503	1.94507
0.4	24843.6	5.17819	3.31665	1.82908
0.45	26444.2	6.31569	2.72878	1.72437
0.5	28112.5	7.67233	2.25395	1.62758
0.55	29886.1	9.32038	1.86174	1.53622
0.6	31811.5	11.3678	1.53175	1.44828
0.65	33951.8	13.9835	1.24976	1.36192
0.7	36398.6	17.4478	1.00551	1.27530
0.75	39297.0	22.2626	0.79142	1.18630
0.8	42899.2	29.4255	0.60171	1.09201
0.85	47705.2	41.2480	0.43179	0.98783
0.9	54931.2	64.5985	0.27789	0.86466
0.95	68984.7	133.318	0.13637	0.69732
0.97	81123.4	223.245	0.08218	0.59836

DRI values calculated at experimental points

Fa	Dose DCA	Dose BL	DRI DCA	DRI BL
0.66694	34740.6	15.0433	1.28211	1.46893
0.50932	28433.9	7.95478	2.09871	1.55351
0.27877	21039.8	3.05227	6.21180	2.38435
0.69409	36089.3	16.9807	1.33188	1.65811
0.41939	25458.3	5.59678	1.87908	1.09301
0.27234	20834.2	2.95841	6.15111	2.31103

DRI Plot for Combo: DCA-BL (DCA+BL [2645.88:1])

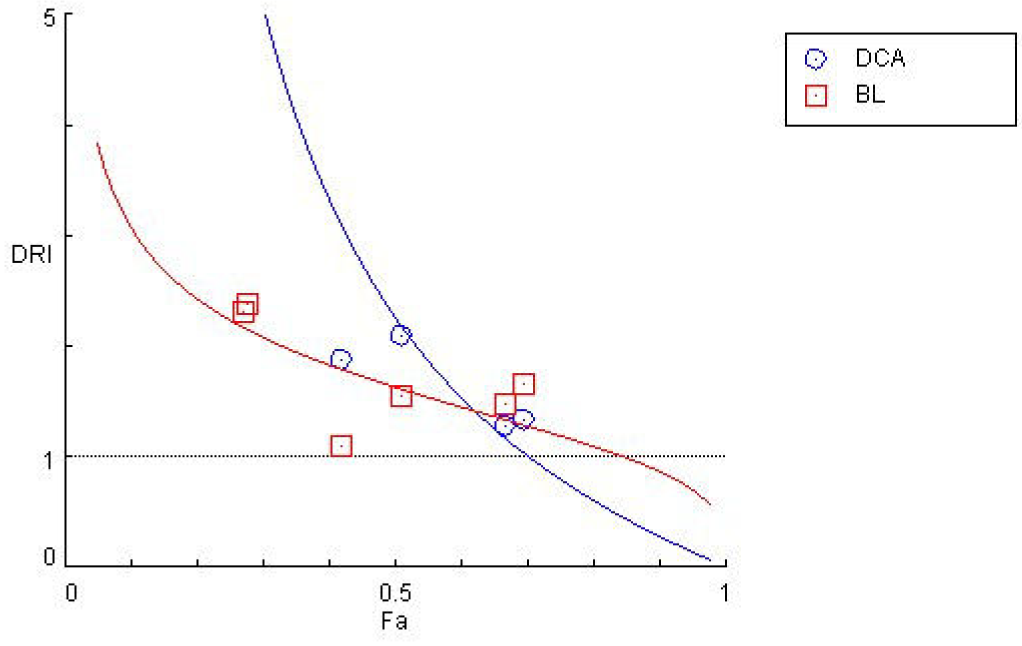

Log(DRI) Plot for Combo: DCA-BL (DCA+BL [2645.88:1])

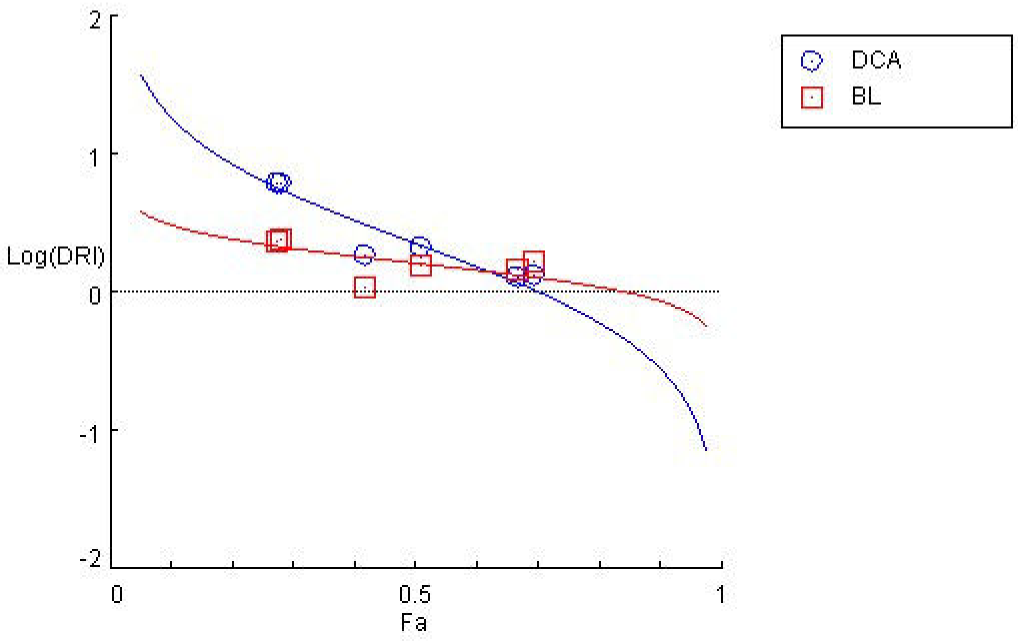

Isobologram for Combo: DCA-BL (DCA+BL [2645.88:1])

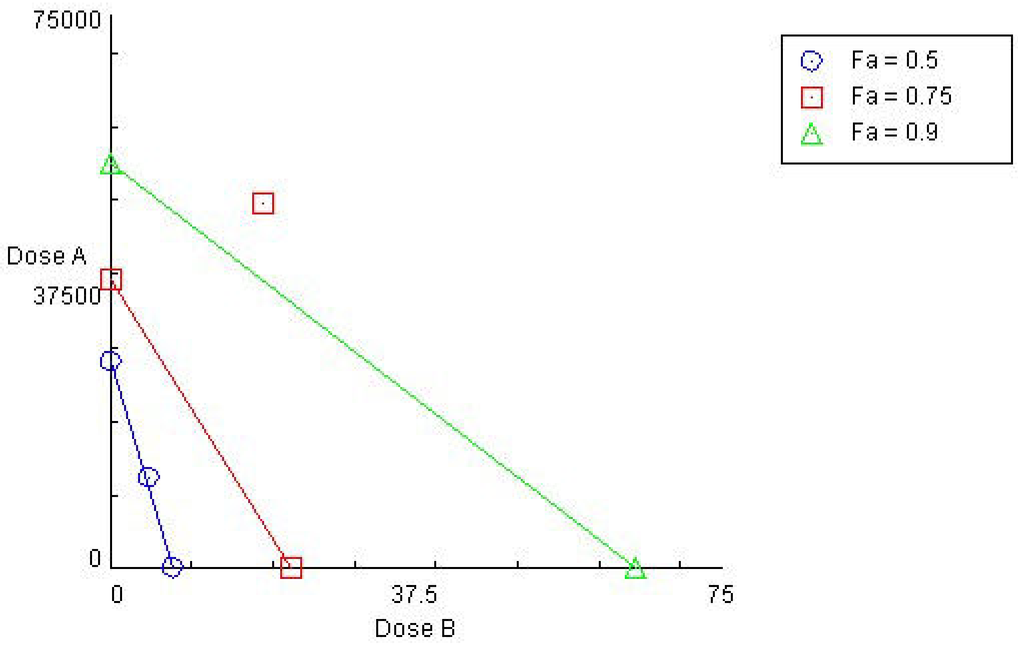

Polygonogram at Fa = 0.9

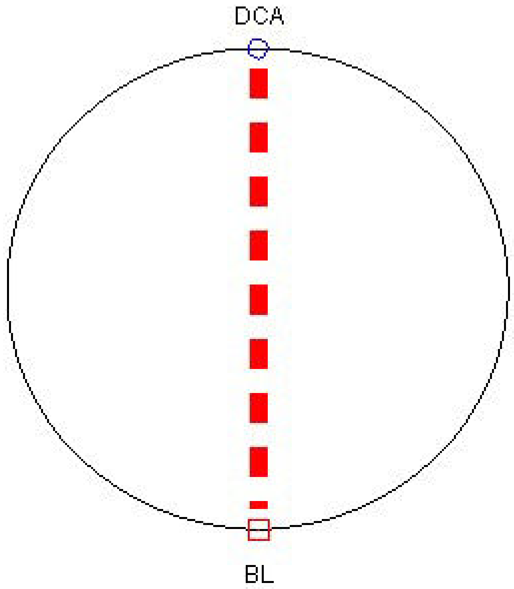
DCA
BL

…Data for Drug: DON [uM]

Dose	Effect
100.0	0.38166
100.0	0.48012
100.0	0.48384
200.0	0.33739
200.0	0.36386
200.0	0.39423
500.0	0.40269
500.0	0.37869
500.0	0.36816
1000.0	0.46954
1000.0	0.44890
1000.0	0.35436
1505.33	0.43321
1505.33	0.46825

14 data points entered.

X-int: 10.4241
Y-int: -0.2083 +/- 0.15697
m: 0.01998 +/- 0.05965
Dm: 2.66E10
r: 0.09625

Data for Drug: BL [uM]

Dose	Effect
60.5	0.50542
10.0	0.17796
10.0	0.11899
20.0	0.29743
20.0	0.23117
50.0	0.50862
50.0	0.45673
100.0	0.67143
100.0	0.60417
60.5	0.60304

10 data points entered.

X-int: 1.73002
Y-int: -1.7977 +/- 0.12684
m: 1.03911 +/- 0.07942
Dm: 53.7054
r: 0.97742

Data for Drug Combo: DON-BL (DON+BL [24.8815:1])

Dose A	Effect
1505.33+	0.60379

Dose A	Effect
752.665+	0.5
188.166+	0.40584
1505.33+	0.63869
752.665+	0.53658
188.166+	0.39807

6 data points entered.

X-int: 2.73379

Y-int: -1.1387 +/- 0.13677

m: 0.41653 +/- 0.04852

Dm: 541.745

r: 0.97391

Dose-Effect Curve

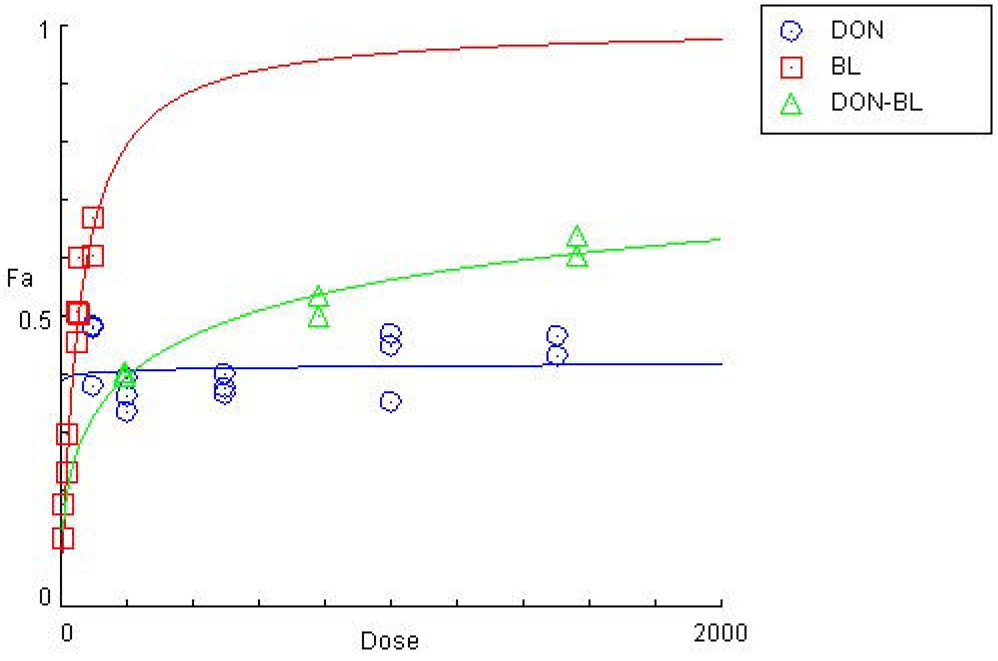

Median-Effect Plot

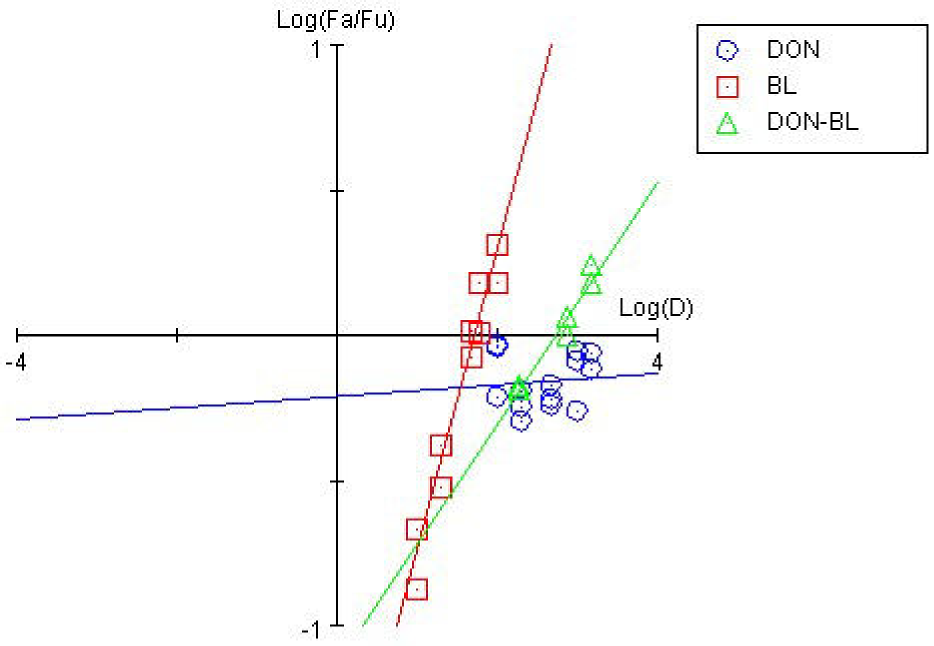

CI Data for Drug Combo: DON-BL (DON+BL [24.8815:1])

Fa	CI Value	S.D.A. Analysis
0.05	1.63E53	Infinit +/- NaN
0.1	5.66E37	Infinit +/- NaN
0.15	1.52E28	4.0E246 +/- Infinit
0.2	9.43E20	6.4E182 +/- Infinit
0.25	1.05E15	1.3E130 +/- 1.5E131
0.3	6.652E9	1.25E84 +/- 1.40E85
0.35	125991.	2.11E42 +/- 2.21E43
0.4	5.02079	5.930E8 +/- 6.677E9
0.45	0.29231	2.54E11 +/- 2.86E12
0.5	0.38975	1.91E26 +/- 2.15E27
0.55	0.52017	1.44E41 +/- 1.62E42
0.6	0.69837	2.19E56 +/- 2.46E57
0.65	0.94952	1.49E72 +/- 1.68E73
0.7	1.31856	1.24E89 +/- 1.40E90
0.75	1.89277	5.3E107 +/- 6.0E108
0.8	2.86295	1.1E129 +/- 1.3E130
0.85	4.72501	7.5E154 +/- Infinit
0.9	9.19192	1.5E189 +/- Infinit
0.95	26.9275	3.7E244 +/- Infinit
0.97	57.8531	9.5E283 +/- Infinit

CI values for actual experimental points:

Total Dose	Fa	CI Value
1565.83	0.60379	0.75104
782.915	0.5	0.56326
195.729	0.40584	1.56652
1565.83	0.63869	0.65108

Total Dose	Fa	CI Value
782.915	0.53658	0.48916
195.729	0.39807	7.08268

Combination Index Plot

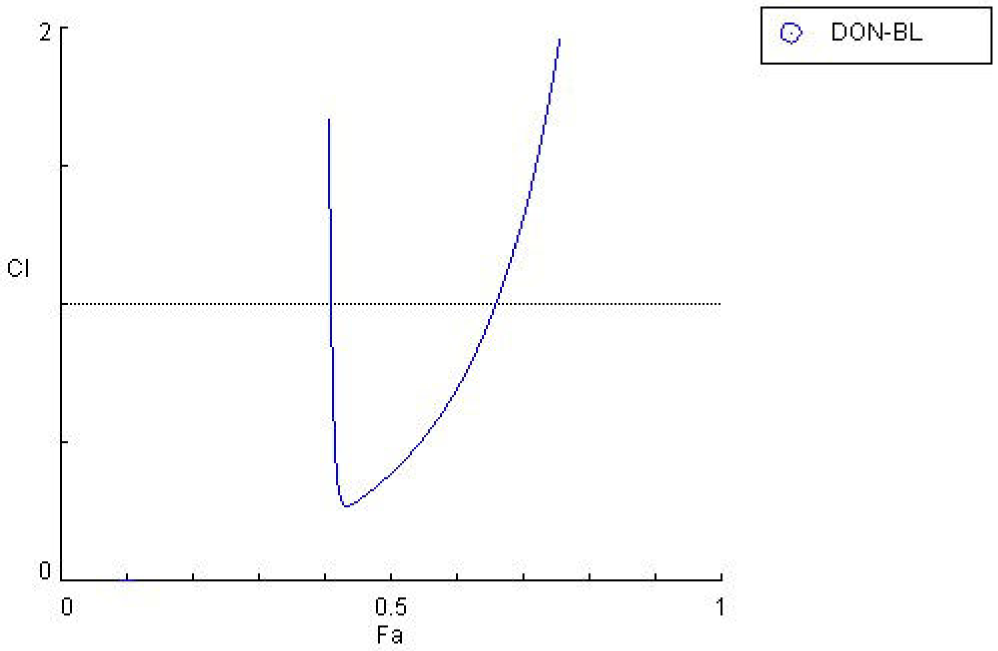

Logarithmic Combination Index Plot

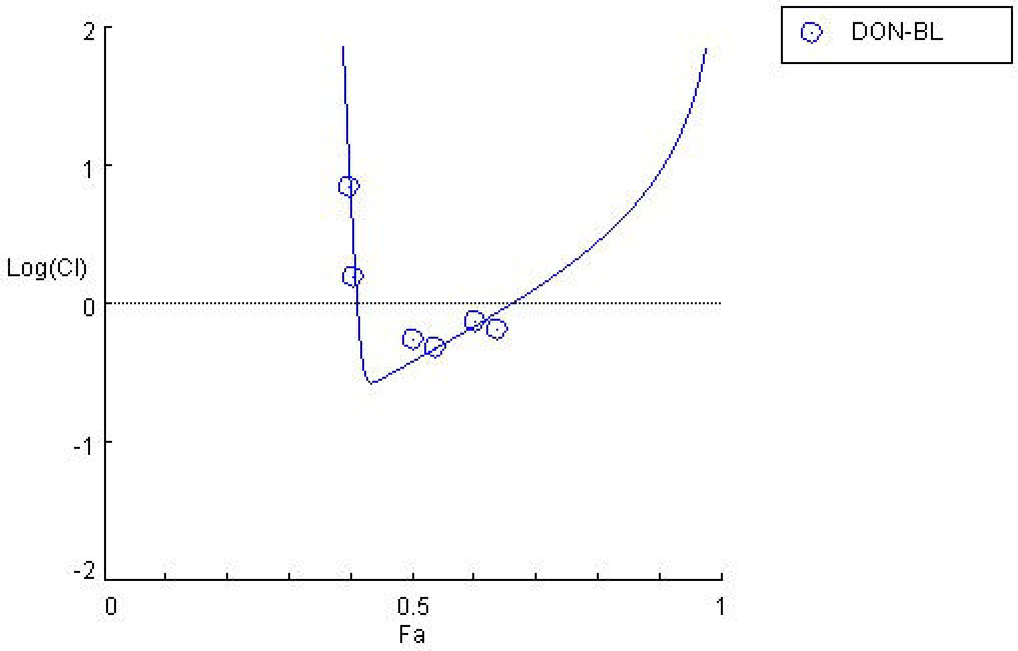

DRI Data for Drug Combo: DON-BL (DON+BL [24.8815:1])

Fa	Dose DON	Dose BL	DRI DON	DRI BL
0.05	2.7E-54	3.15786	6.1E-54	177.264
0.1	4.7E-38	6.48172	1.8E-38	60.5107
0.15	5.3E-28	10.1168	6.6E-29	31.1049
0.2	2.0E-20	14.1455	1.1E-21	18.8469

Fa	Dose DON	Dose BL	DRI DON	DRI BL
0.25	3.5E-14	18.6575	9.5E-16	12.4601
0.3	1.02E-8	23.7624	1.5E-10	8.68010
0.35	9.35E-4	29.6000	7.94E-6	6.25073
0.4	40.9623	36.3542	0.20819	4.59737
0.45	1156294	44.2739	3594.32	3.42431
0.5	2.66E10	53.7054	5.099E7	2.56574
0.55	6.10E14	65.1461	7.23E11	1.92244
0.6	1.72E19	79.3381	1.25E16	1.43191
0.65	7.54E23	97.4417	3.28E20	1.05316
0.7	6.89E28	121.379	1.73E25	0.75840
0.75	1.99E34	154.590	2.74E30	0.52833
0.8	3.56E40	203.900	2.45E36	0.34929
0.85	1.32E48	285.097	3.94E43	0.21164
0.9	1.50E58	444.985	1.47E53	0.10879
0.95	2.59E74	913.362	4.24E68	0.03714
0.97	9.30E85	1523.53	4.24E79	0.01729

DRI values calculated at experimental points

Fa	Dose DON	Dose BL	DRI DON	DRI BL
0.60379	3.80E19	80.5550	2.52E16	1.33149
0.5	2.66E10	53.7054	3.528E7	1.77539
0.40584	138.022	37.2135	0.73351	4.92079
0.63869	6.38E22	92.9227	4.24E19	1.53591
0.53658	4.07E13	61.8409	5.40E10	2.04433
0.39807	27.3775	36.0736	0.14550	4.77005

DRI Plot for Combo: DON-BL (DON+BL [24.8815:1])

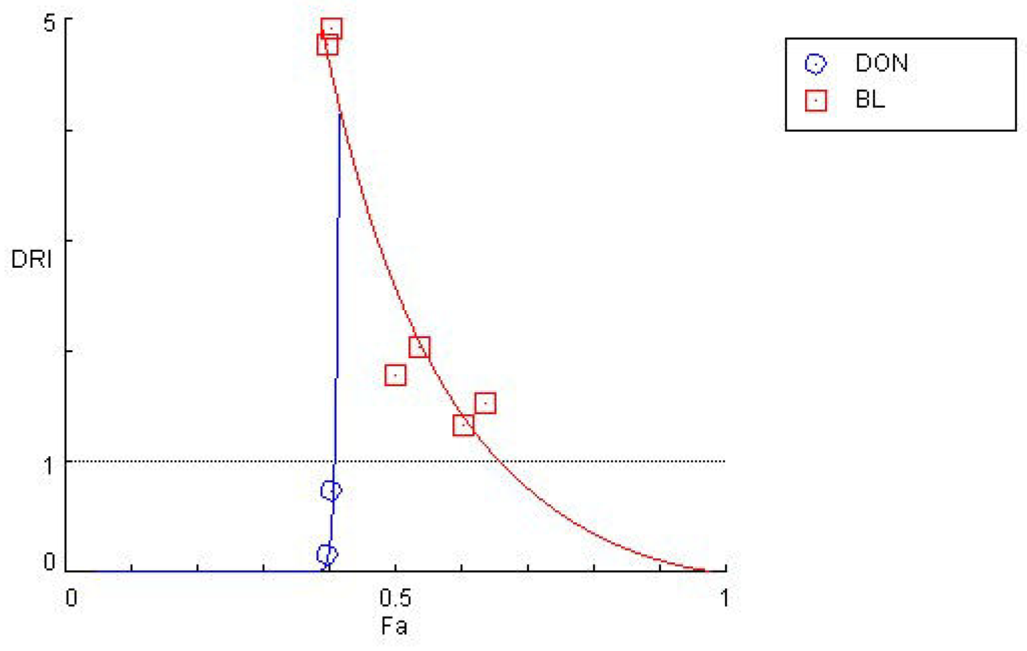

Log(DRI) Plot for Combo: DON-BL (DON+BL [24.8815:1])

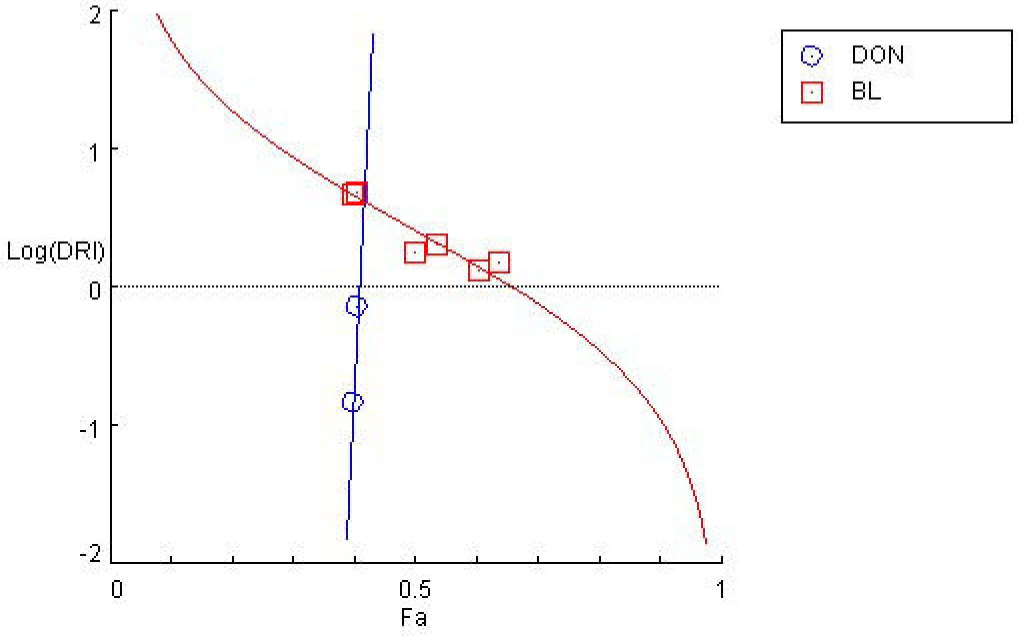

Isobologram for Combo: DON-BL (DON+BL [24.8815:1])

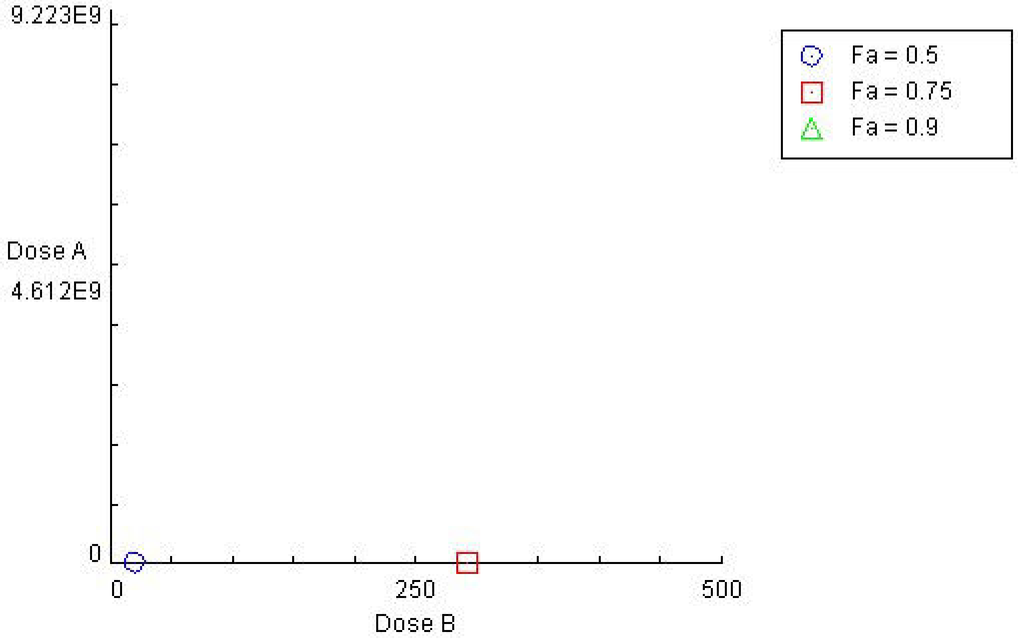

Polygonogram at Fa = 0.9

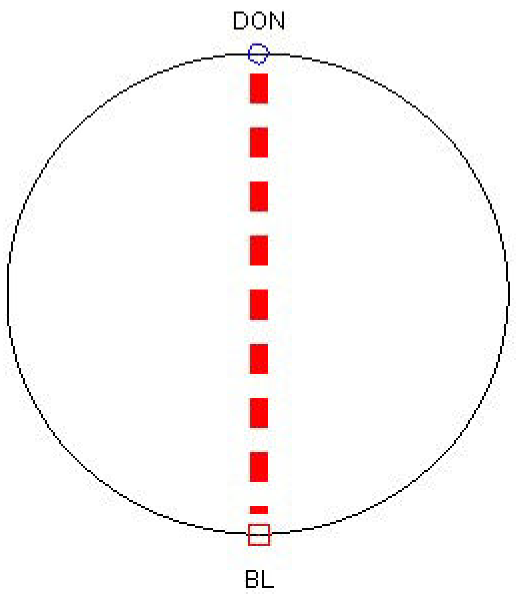
DON
BL

…Data for Drug: DON [uM]

Dose	Effect
100.0	0.47294
100.0	0.39925
100.0	0.47132
200.0	0.57530
200.0	0.52211
500.0	0.60422
500.0	0.63465
500.0	0.58732
200.0	0.53749
1000.0	0.68330
1000.0	0.62196
1000.0	0.59860
198.43	0.39312
198.43	0.27206

14 data points entered.

X-int: 2.35372
Y-int: -0.9248 +/- 0.27953
m: 0.39292 +/- 0.11194
Dm: 225.796
r: 0.71175

Data for Drug: BL [uM]

Dose	Effect
50.0	0.47634
50.0	0.54304
100.0	0.60608
100.0	0.64751
200.0	0.66695
200.0	0.70512
55.09	0.59058
55.09	0.57190

8 data points entered.

X-int: 1.54431
Y-int: -0.7160 +/- 0.18542
m: 0.46364 +/- 0.09508
Dm: 35.0196
r: 0.89360

Data for Drug Combo: DON-BL (DON+BL [3.6019:1])

Dose A	Effect
198.430+	0.56209
99.2149+	0.49428
24.8037+	0.33170

Dose A	Effect
198.430+	0.64855
99.2149+	0.53804
24.8037+	0.25181

6 data points entered.

X-int: 2.09208

Y-int: -1.3503 +/- 0.19487

m: 0.64542 +/- 0.09564

Dm: 123.619

r: 0.95878

Dose-Effect Curve

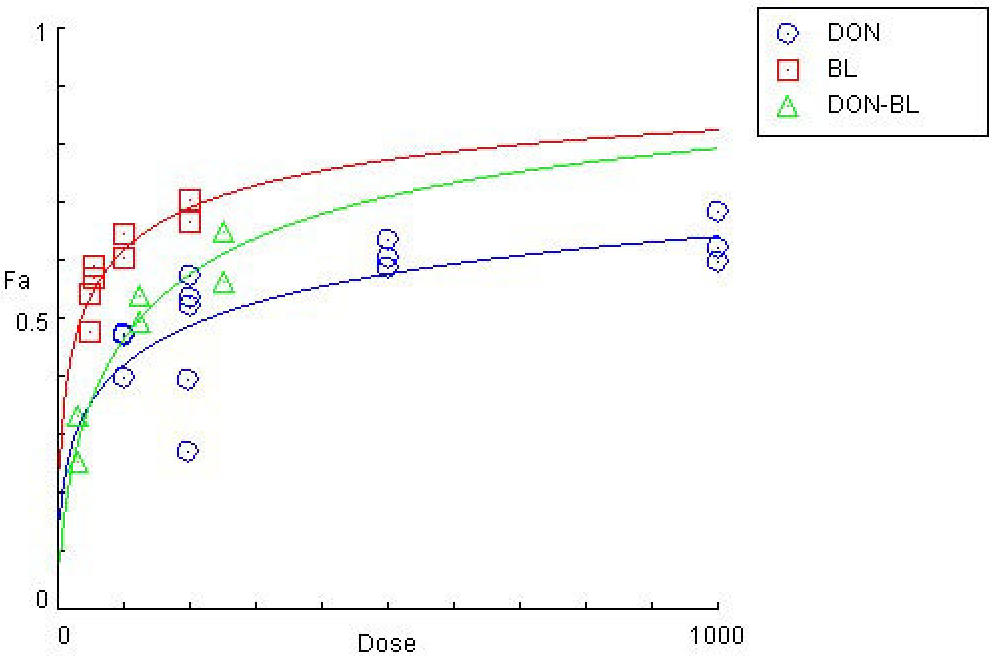

Median-Effect Plot

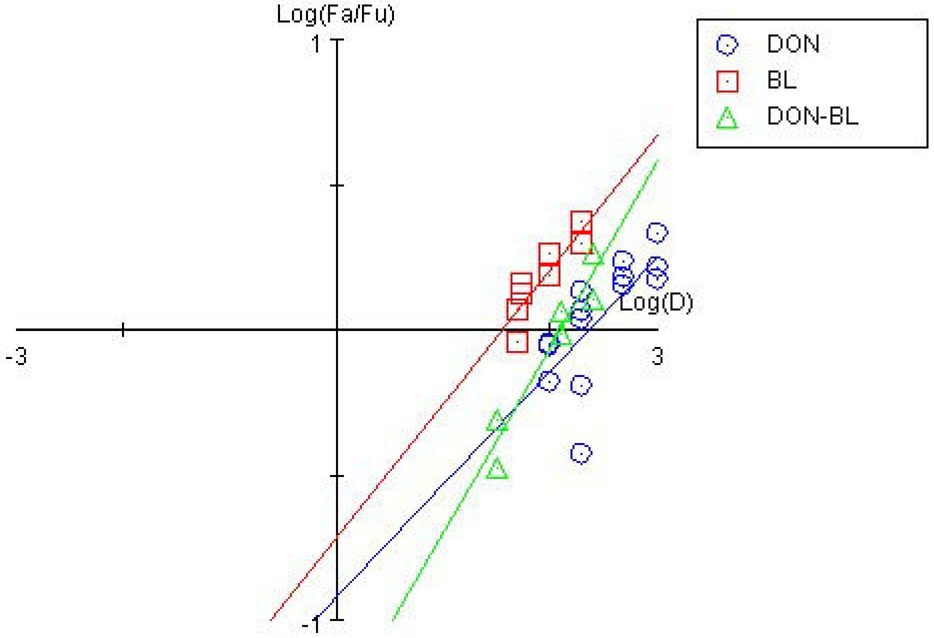

CI Data for Drug Combo: DON-BL (DON+BL [3.6019:1])

Fa	CI Value	S.D.A. Analysis
0.05	12.6254	13.8071 +/- 10.6875
0.1	6.73367	7.10759 +/- 4.09758
0.15	4.60996	4.78300 +/- 2.22014
0.2	3.48422	3.57694 +/- 1.37324
0.25	2.77443	2.82773 +/- 0.90551
0.3	2.27955	2.31125 +/- 0.61605
0.35	1.91089	1.92999 +/- 0.42385
0.4	1.62293	1.63447 +/- 0.29065
0.45	1.38979	1.39678 +/- 0.19673
0.5	1.19558	1.19992 +/- 0.13206
0.55	1.02995	1.03288 +/- 0.09302
0.6	0.88579	0.88817 +/- 0.07866
0.65	0.75802	0.76044 +/- 0.08222
0.7	0.64280	0.64568 +/- 0.09254
0.75	0.53709	0.54074 +/- 0.10279
0.8	0.43824	0.44289 +/- 0.11018
0.85	0.34364	0.34944 +/- 0.11337
0.9	0.25000	0.25701 +/- 0.11081
0.95	0.15110	0.15912 +/- 0.09848
0.97	0.10631	0.11445 +/- 0.08758

CI values for actual experimental points:

Total Dose	Fa	CI Value
253.52	0.56209	1.38368
126.76	0.49428	1.29209
31.69	0.33170	1.54415
253.52	0.64855	0.60444
126.76	0.53804	0.86422
31.69	0.25181	3.81488

Combination Index Plot

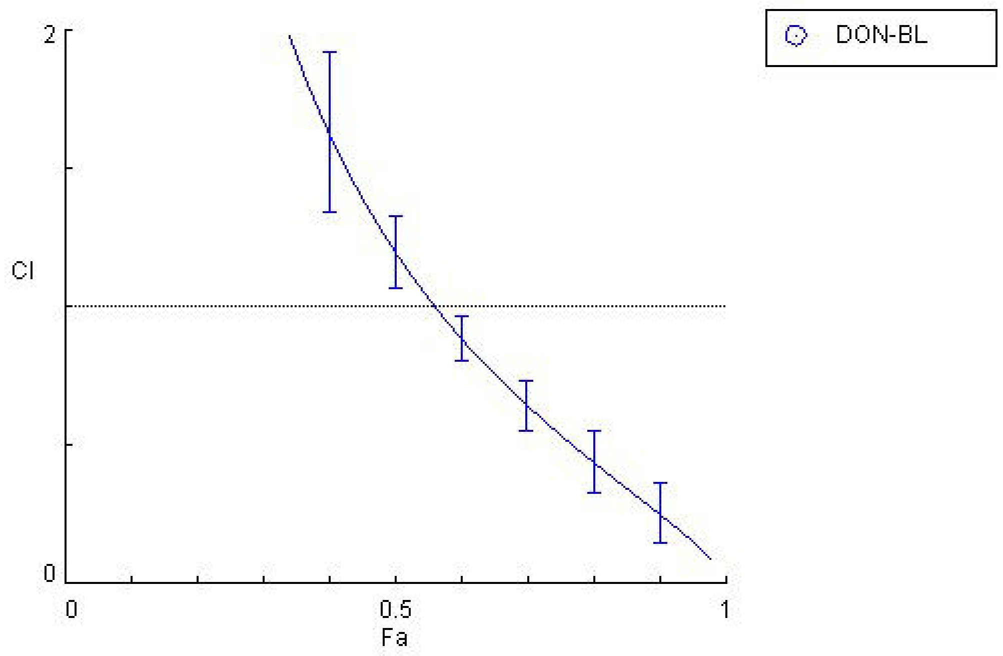

Logarithmic Combination Index Plot

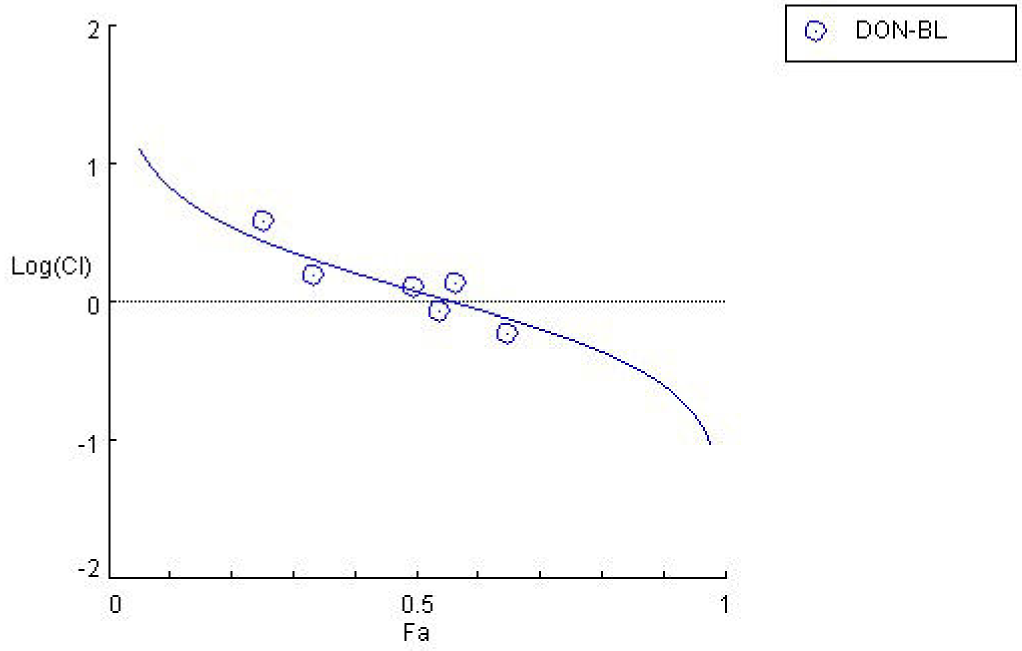

DRI Data for Drug Combo: DON-BL (DON+BL [3.6019:1])

Fa	Dose DON	Dose BL	DRI DON	DRI BL
0.05	0.12568	0.06113	0.12441	0.21797
0.1	0.84169	0.30632	0.26180	0.34318
0.15	2.73202	0.83084	0.41496	0.45453
0.2	6.62923	1.76106	0.58696	0.56163
0.25	13.7859	3.27525	0.78163	0.66887
0.3	26.1342	5.63184	1.00385	0.77919
0.35	46.7197	9.21424	1.25999	0.89507
0.4	80.4564	14.6054	1.55853	1.01906
0.45	135.493	22.7166	1.91103	1.15405

Fa	Dose DON	Dose BL	DRI DON	DRI BL
0.5	225.796	35.0196	2.33366	1.30366
0.55	376.283	53.9857	2.84975	1.47266
0.6	633.681	83.9671	3.49429	1.66774
0.65	1091.27	133.096	4.32223	1.89876
0.7	1950.85	217.757	5.42508	2.18116
0.75	3698.26	374.436	6.96744	2.54089
0.8	7690.75	696.384	9.27825	3.02606
0.85	18661.5	1476.07	13.1242	3.73907
0.9	60573.0	4003.54	20.8022	4.95230
0.95	405663.	20061.2	43.7726	7.79698
0.97	1569646	63148.2	74.3174	10.7692

DRI values calculated at experimental points

Fa	Dose DON	Dose BL	DRI DON	DRI BL
0.56209	426.241	60.0015	2.14807	1.08915
0.49428	213.025	33.3336	2.14711	1.21015
0.33170	37.9710	7.72942	1.53086	1.12244
0.64855	1073.73	131.281	5.41116	2.38302
0.53804	332.841	48.6551	3.35475	1.76638
0.25181	14.1282	3.34406	0.56960	0.48561

DRI Plot for Combo: DON-BL (DON+BL [3.6019:1])

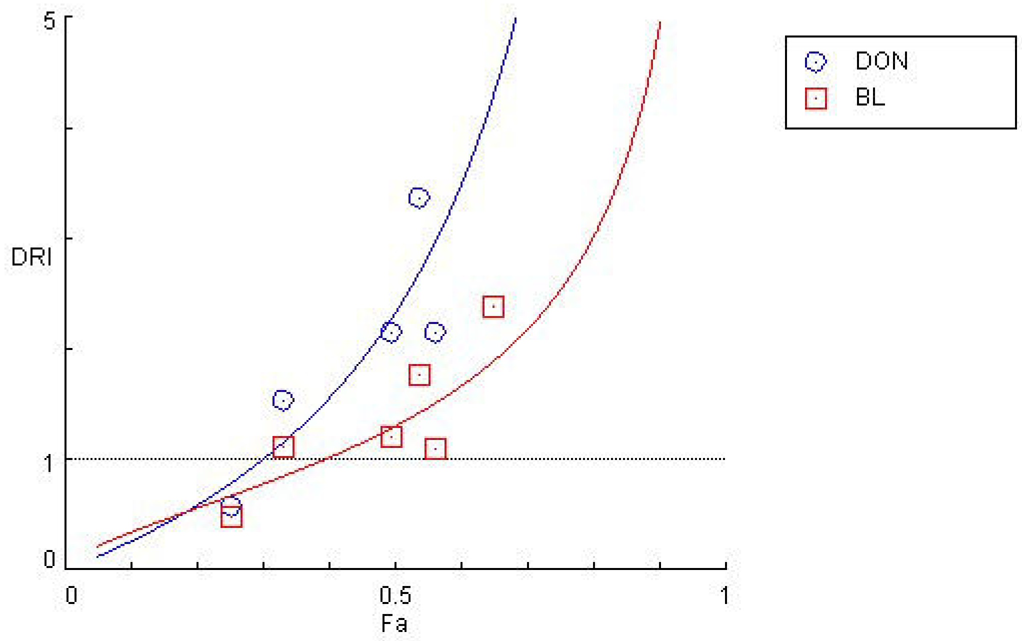

Log(DRI) Plot for Combo: DON-BL (DON+BL [3.6019:1])

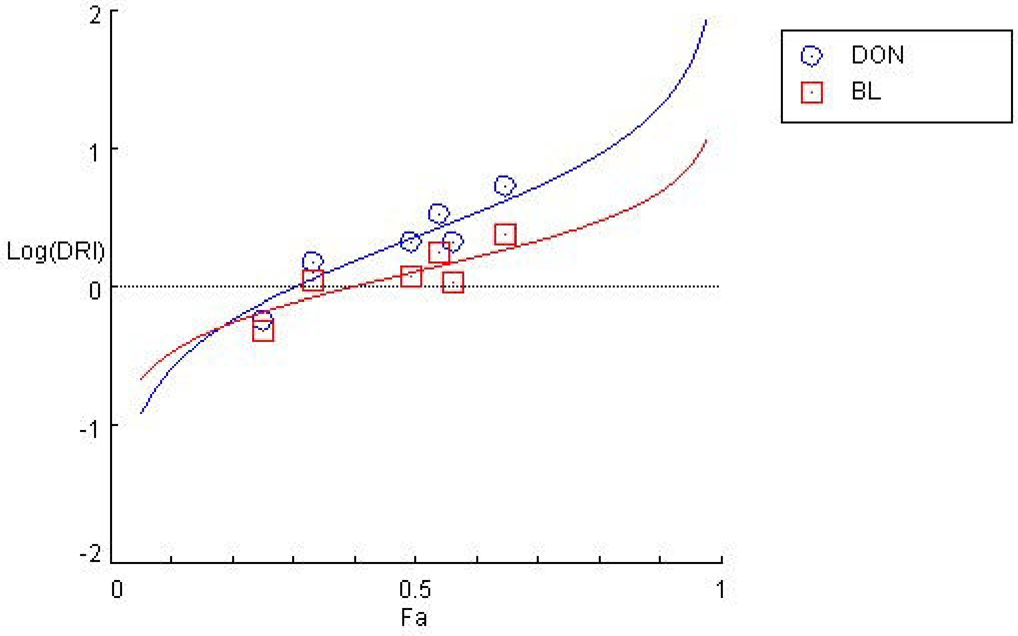

Isobologram for Combo: DON-BL (DON+BL [3.6019:1])

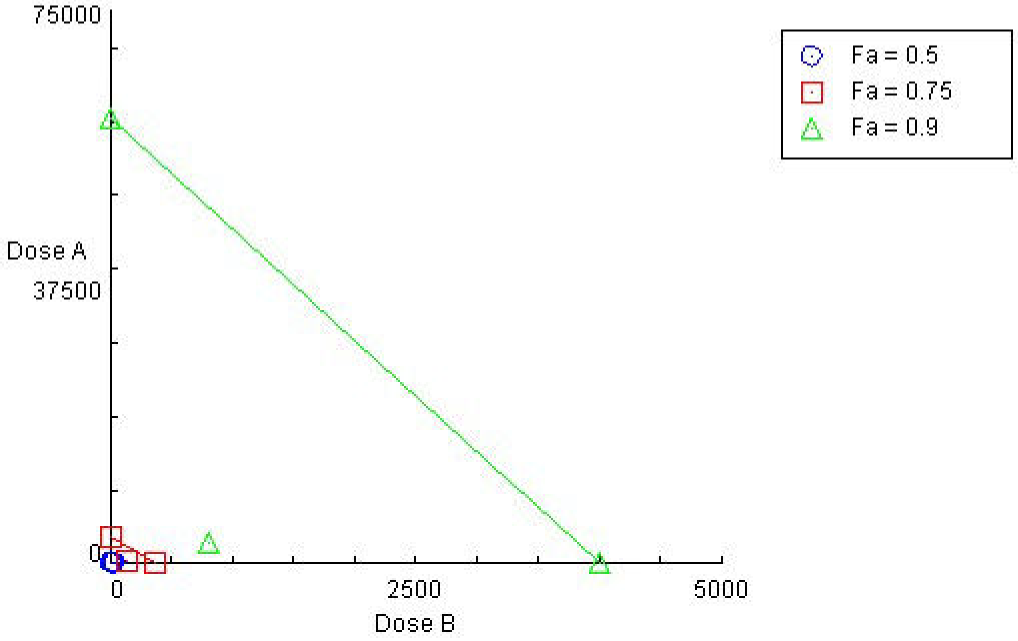

Polygonogram at Fa = 0.9

GBM27 DON-Bleomycin

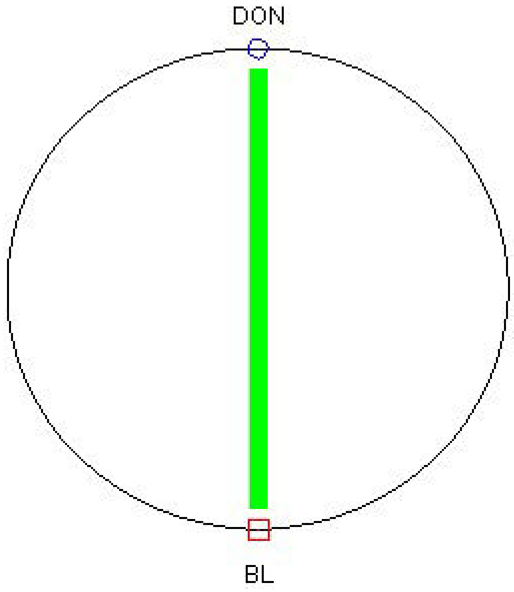

…Data for Drug: DON [uM]

Dose	Effect
100.0	0.23204
100.0	0.54701
100.0	0.56789
200.0	0.18821
200.0	0.50348
200.0	0.49454
500.0	0.26568
500.0	0.47313
500.0	0.51114
1000.0	0.42401
1000.0	0.46522
1000.0	0.55585
286.9	0.47474

13 data points entered.

X-int: 3.97260
Y-int: -0.3243 +/- 0.49090
m: 0.08164 +/- 0.19454
Dm: 9388.60
r: 0.12553

Data for Drug: BL [uM]

Dose	Effect
50.0	0.68241
50.0	0.29706
100.0	0.85466
100.0	0.59909
200.0	0.94684
200.0	0.79812
11.8	0.52598

7 data points entered.

X-int: 1.37768
Y-int: -1.1232 +/- 0.79223
m: 0.81526 +/- 0.41498
Dm: 23.8606
r: 0.66002

Data for Drug Combo: DON-BL (DON+BL [24.3135:1])

Dose A	Effect
286.900+	0.43644
143.450+	0.40651
24.8000+	0.45655

3 data points entered.

X-int: -0.3459

Y-int: -0.0171 +/- 0.13392
m: -0.0494 +/- 0.06471
Dm: 0.45091
r: -0.6072

Dose-Effect Curve

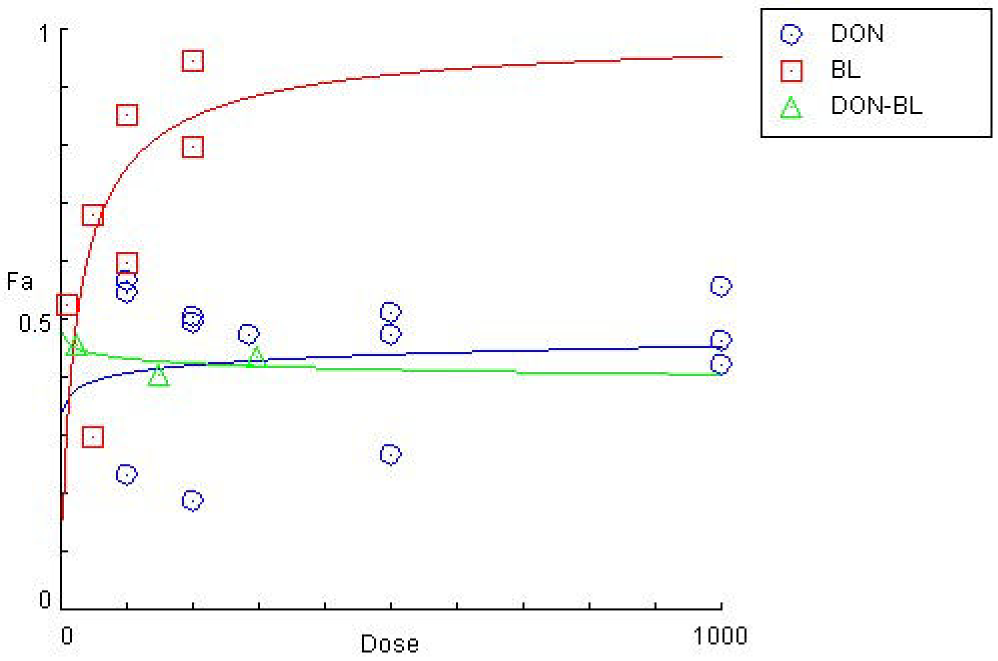

Median-Effect Plot

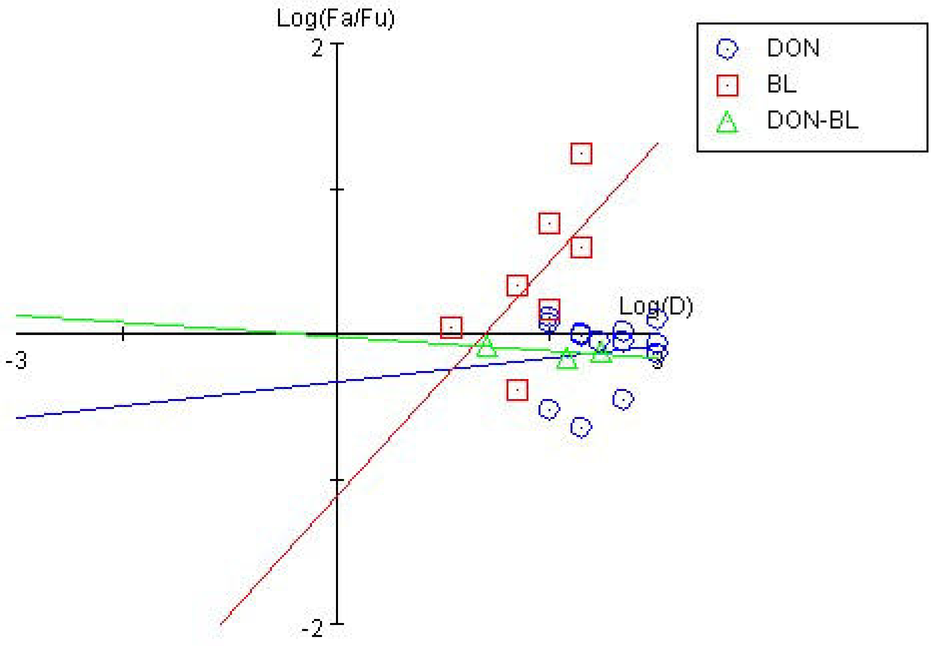

CI Data for Drug Combo: DON-BL (DON+BL [24.3135:1])

Fa	CI Value	S.D.A. Analysis
0.05	1.54E37	4.56E93 +/- 4.55E94
0.1	4.47E26	3.92E66 +/- 3.92E67
0.15	1.34E20	6.86E49 +/- 6.86E50

Fa	CI Value	S.D.A. Analysis
0.2	1.64E15	1.67E37 +/- 1.66E38
0.25	1.44E11	7.19E26 +/- 6.33E27
0.3	4.107E7	1.80E18 +/- 1.37E19
0.35	25207.1	1.03E11 +/- 9.99E11
0.4	28.5697	22169.6 +/- 220652.
0.45	0.08645	0.15026 +/- 0.48830
0.5	7.93E-4	0.08943 +/- 0.85358
0.55	1.02E-5	0.19966 +/- 1.96620
0.6	1.25E-7	0.48640 +/- 4.83248
0.65	1.28E-9	1.24479 +/- 12.4122
0.7	9.5E-12	3.40836 +/- 34.0352
0.75	4.4E-14	10.3429 +/- 103.340
0.8	9.1E-17	36.8717 +/- 368.471
0.85	5.2E-20	171.851 +/- 1717.47
0.9	2.5E-24	1327.50 +/- 13267.1
0.95	2.8E-31	36067.7 +/- 360463.
0.97	3.1E-36	378009. +/- 3777852

CI values for actual experimental points:

Total Dose	Fa	CI Value
298.7	0.43644	1.37645
149.35	0.40651	1.96734
25.82	0.45655	0.07526

Combination Index Plot

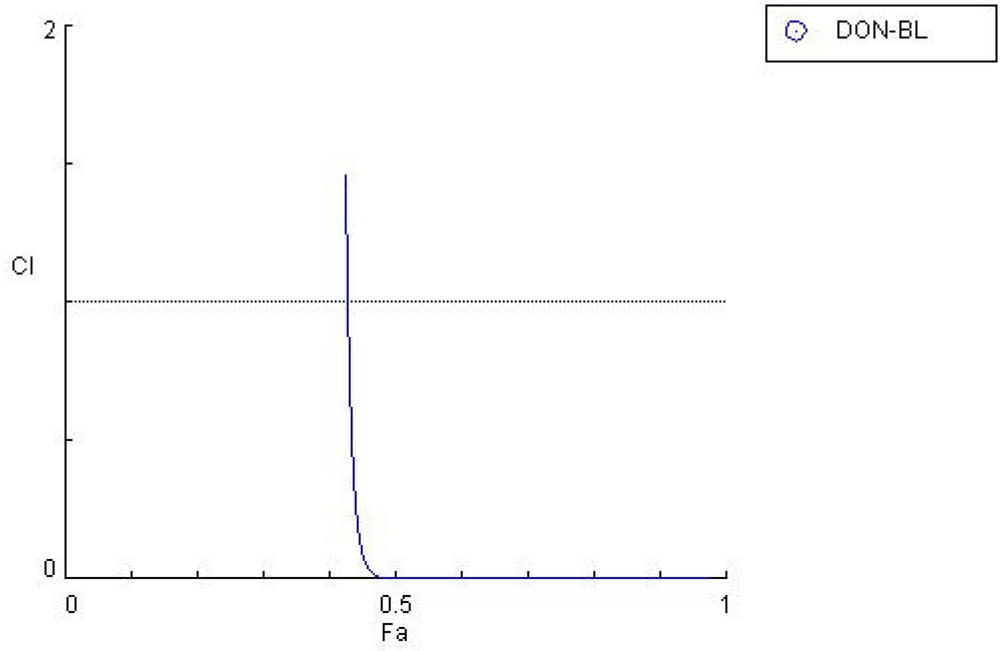

Logarithmic Combination Index Plot

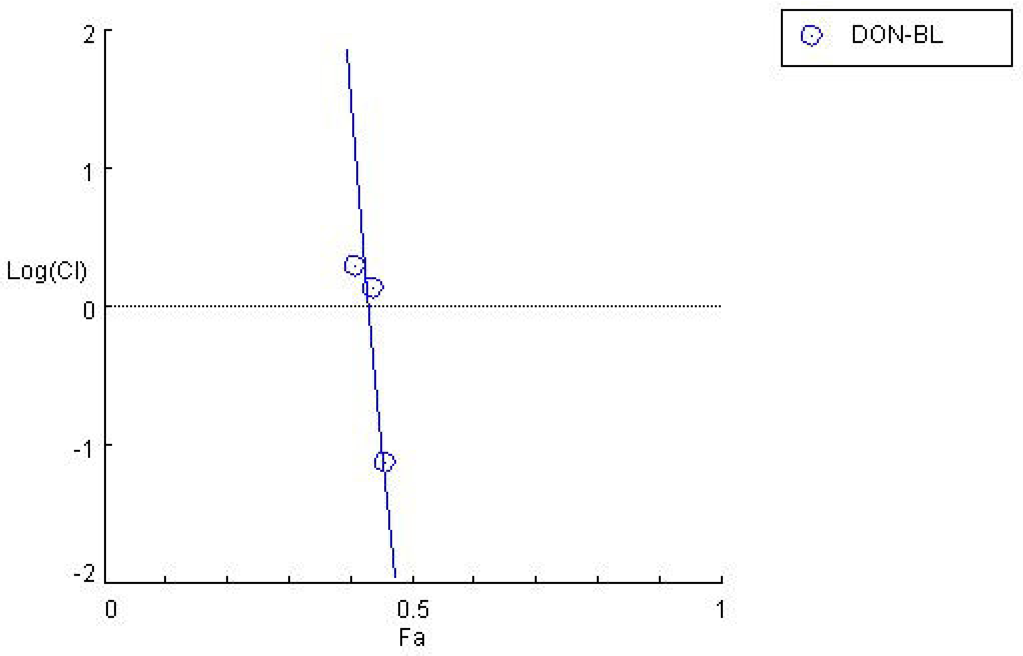

DRI Data for Drug Combo: DON-BL (DON+BL [24.3135:1])

Fa	Dose DON	Dose BL	DRI DON	DRI BL
0.05	2.0E-12	0.64440	6.5E-38	5.0E-25
0.1	1.93E-8	1.61139	2.2E-27	4.6E-18
0.15	5.56E-6	2.84214	7.5E-21	9.3E-14
0.2	3.97E-4	4.35703	6.1E-16	1.6E-10
0.25	0.01345	6.20071	7.0E-12	7.81E-8
0.3	0.29203	8.43954	2.44E-8	1.71E-5
0.35	4.78232	11.1664	4.04E-5	0.00229
0.4	65.4245	14.5106	0.04149	0.22375
0.45	803.783	18.6544	32.0681	18.0953
0.5	9388.60	23.8606	21677.9	1339.51
0.55	109664.	30.5197	1.465E7	99157.8
0.6	1347291	39.2352	1.13E10	8019048
0.65	1.843E7	50.9857	1.16E13	7.829E8
0.7	3.018E8	67.4596	1.93E16	1.05E11
0.75	6.556E9	91.8165	6.75E19	2.30E13
0.8	2.22E11	130.669	7.69E23	1.10E16
0.85	1.58E13	200.317	6.28E28	1.93E19
0.9	4.58E15	353.314	2.10E35	3.94E23
0.95	4.32E19	883.502	7.24E45	3.60E30
0.97	2.91E22	1696.01	2.28E53	3.23E35

DRI values calculated at experimental points

Fa	Dose DON	Dose BL	DRI DON	DRI BL
0.43644	409.988	17.4383	1.42903	1.47782
0.40651	91.1361	15.0004	0.63532	2.54243
0.45655	1110.94	19.2689	44.7960	18.8909

DRI Plot for Combo: DON-BL (DON+BL [24.3135:1])

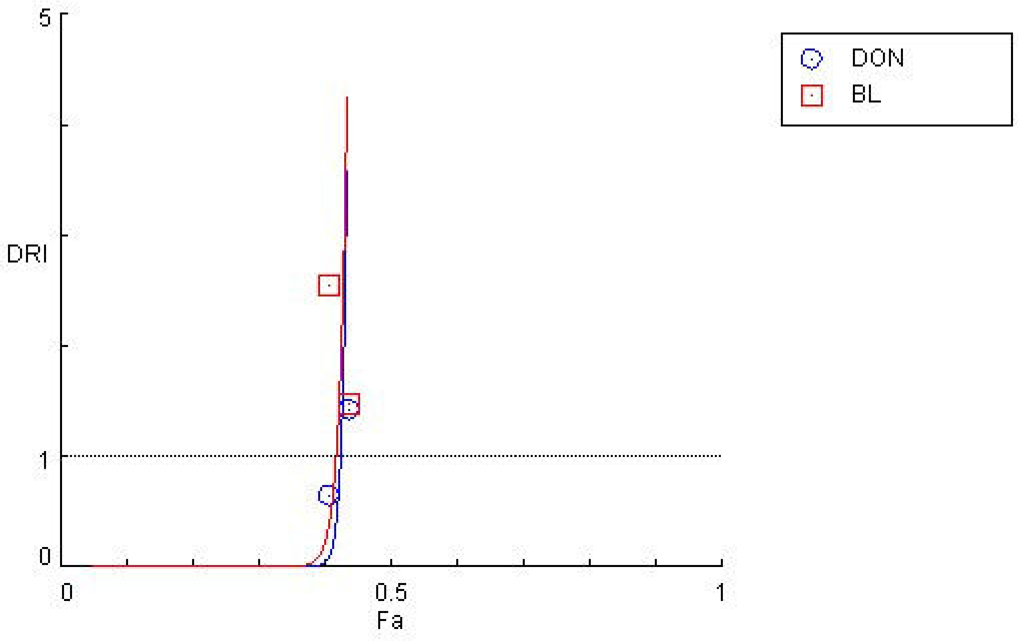

Log(DRI) Plot for Combo: DON-BL (DON+BL [24.3135:1])

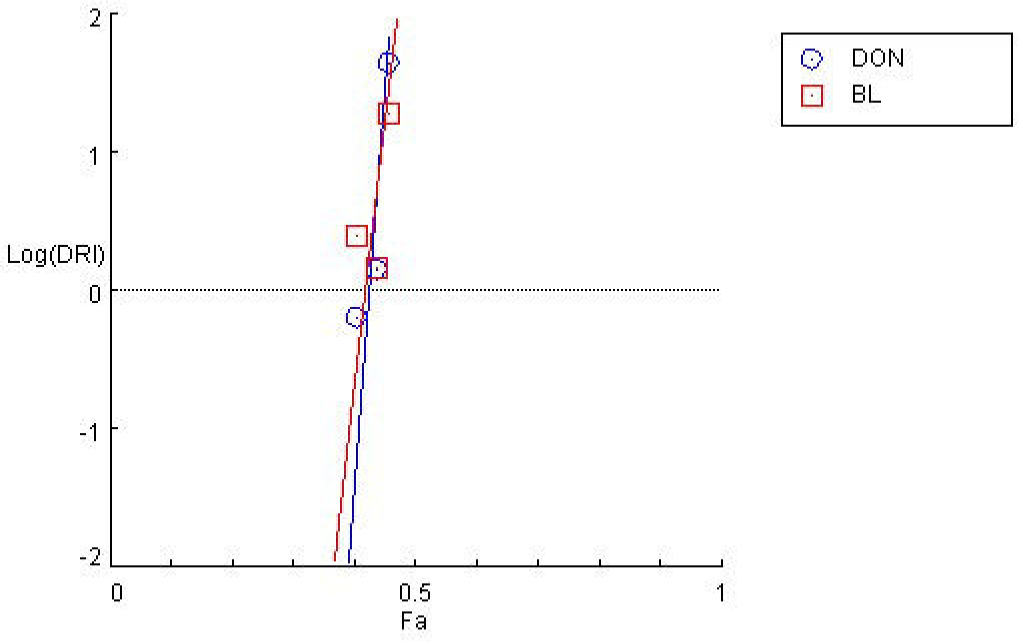

Isobologram for Combo: DON-BL (DON+BL [24.3135:1])

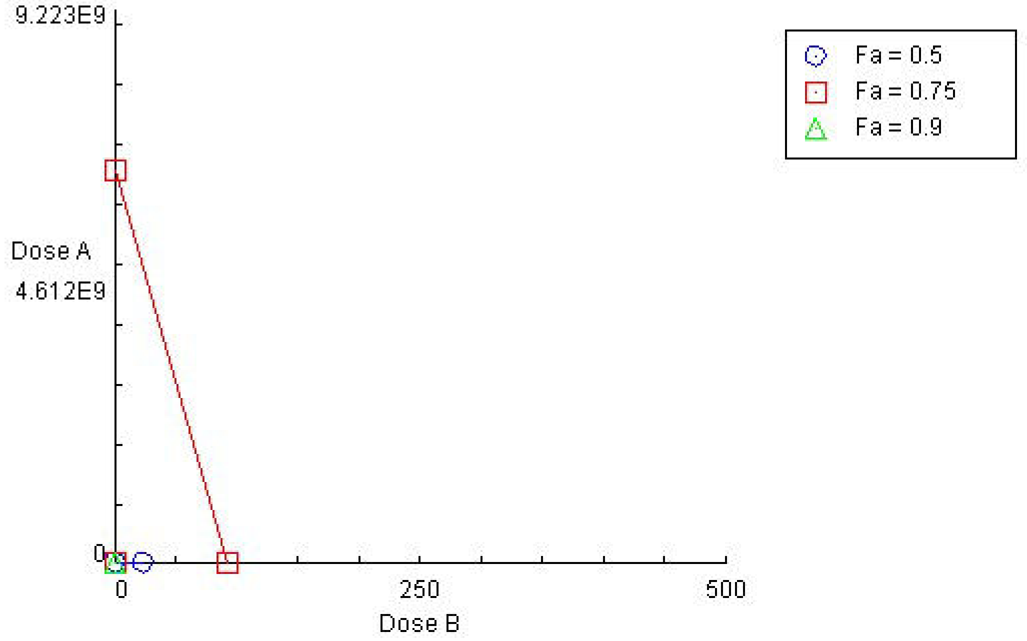

Polygonogram at Fa = 0.9

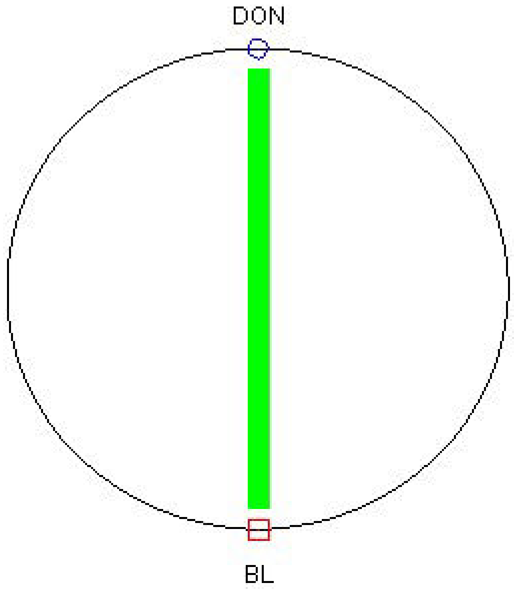

…Data for Drug: DON [uM]

Dose	Effect
100.0	0.59454
100.0	0.58923
100.0	0.63559
200.0	0.60940
200.0	0.61601
200.0	0.62264
500.0	0.60916
500.0	0.66858
500.0	0.64914
1000.0	0.62546
1000.0	0.68916
1000.0	0.67231
99.7	0.57481
99.7	0.60437

14 data points entered.

X-int: 0.51841
Y-int: -0.0609 +/- 0.07125
m: 0.11745 +/- 0.02896
Dm: 3.29925
r: 0.76034

Data for Drug: BL [uM]

Dose	Effect
10.0	0.63176
10.0	0.41297
20.0	0.81331
20.0	0.56917
50.0	0.86886
50.0	0.88044
100.0	0.91241
100.0	0.95258
10.24	0.68528
10.24	0.63144

10 data points entered.

X-int: 0.86442
Y-int: -0.8718 +/- 0.23487
m: 1.00857 +/- 0.16131
Dm: 7.31840
r: 0.91111

Data for Drug Combo: DON-BL (DON+BL [9.7363:1])

Dose A	Effect
99.7000+	0.83571

Dose A	Effect
49.8500+	0.7925
12.4625+	0.45699
99.7000+	0.83863
49.8500+	0.75690
12.4625+	0.50078

6 data points entered.

X-int: 1.16233

Y-int: -0.9834 +/- 0.10928

m: 0.84610 +/- 0.06496

Dm: 14.5320

r: 0.98841

Dose-Effect Curve

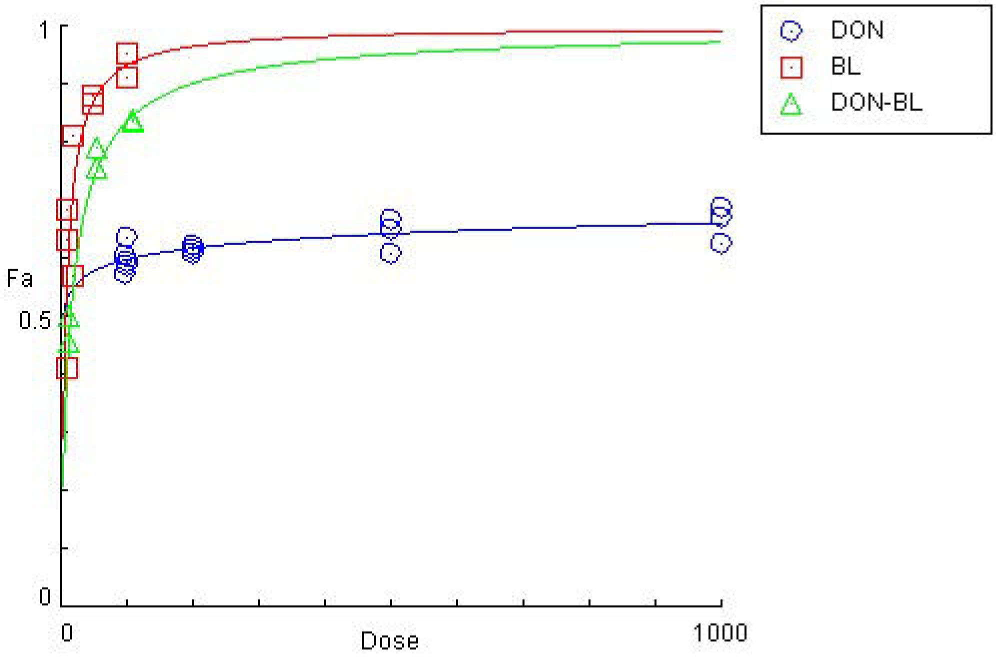

Median-Effect Plot

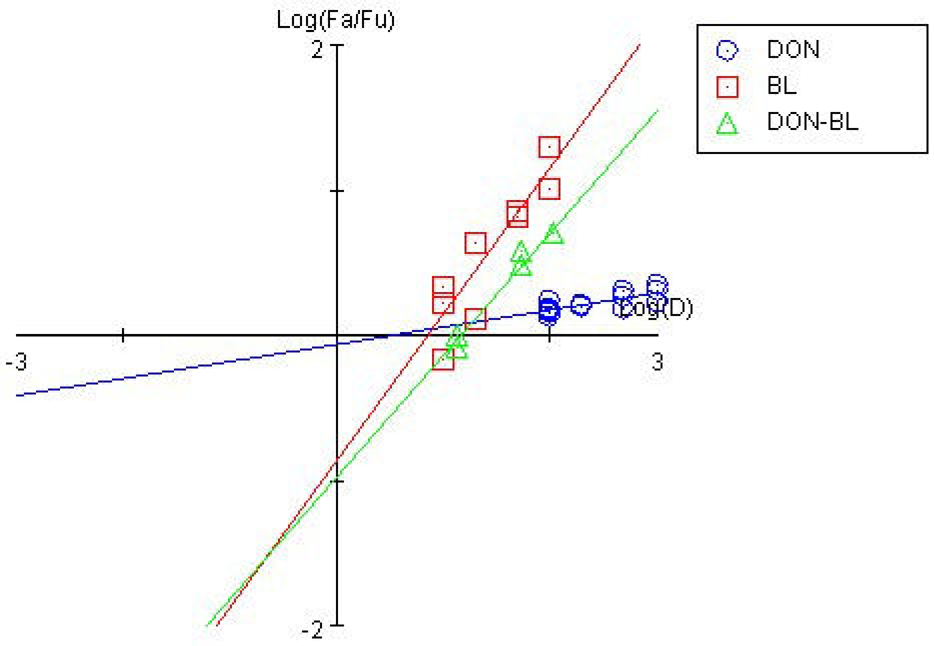

CI Data for Drug Combo: DON-BL (DON+BL [9.7363:1])

Fa	CI Value	S.D.A. Analysis
0.05	9.493E9	7.79E10 +/- 6.85E11
0.1	3.963E7	1.332E8 +/- 8.722E8
0.15	1333321	2912305 +/- 1.401E7
0.2	103711.	175919. +/- 621719.
0.25	12582.4	18123.3 +/- 47210.6
0.3	1993.12	2565.61 +/- 4931.21
0.35	373.978	444.183 +/- 627.182
0.4	78.2570	87.6137 +/- 89.5961
0.45	17.5739	18.8281 +/- 13.4676
0.5	4.17934	4.33352 +/- 1.98537
0.55	1.10934	1.12417 +/- 0.25730
0.6	0.40412	0.40488 +/- 0.03133
0.65	0.25077	0.25087 +/- 0.01965
0.7	0.22533	0.22548 +/- 0.01684
0.75	0.22925	0.22932 +/- 0.01539
0.8	0.24097	0.24097 +/- 0.01513
0.85	0.25733	0.25729 +/- 0.01702
0.9	0.28101	0.28098 +/- 0.02326
0.95	0.32397	0.32415 +/- 0.04014
0.97	0.35848	0.35900 +/- 0.05642

CI values for actual experimental points:

Total Dose	Fa	CI Value
109.94	0.83571	0.27892
54.97	0.7925	0.18544
13.7425	0.45699	16.6096
109.94	0.83863	0.27307

**Total Dose**	**Fa**	**CI Value**
54.97	0.75690	0.22784
13.7425	0.50078	3.85261

Combination Index Plot

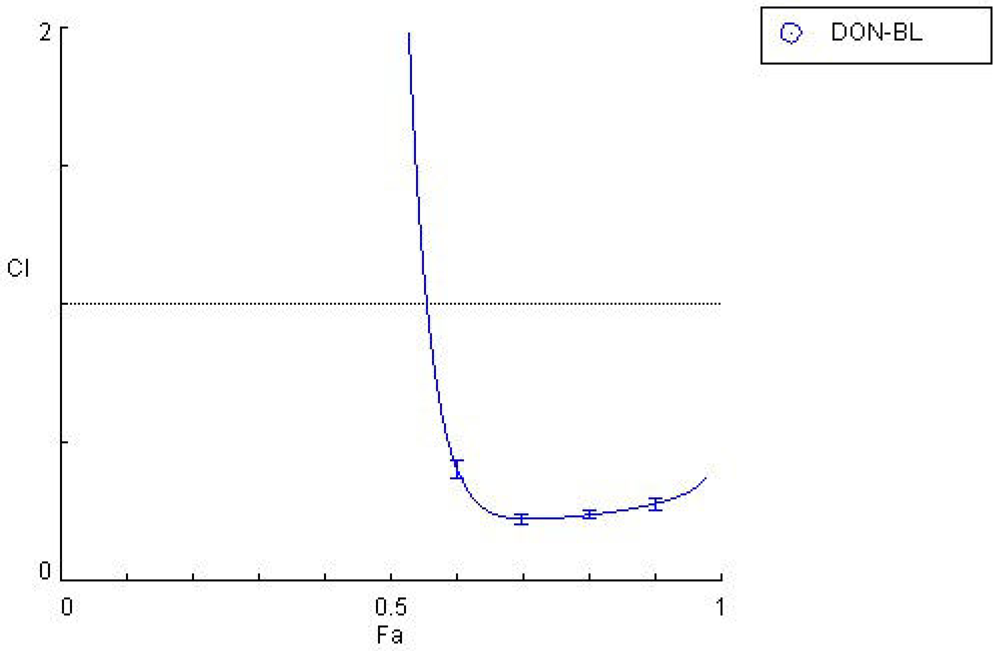

Logarithmic Combination Index Plot

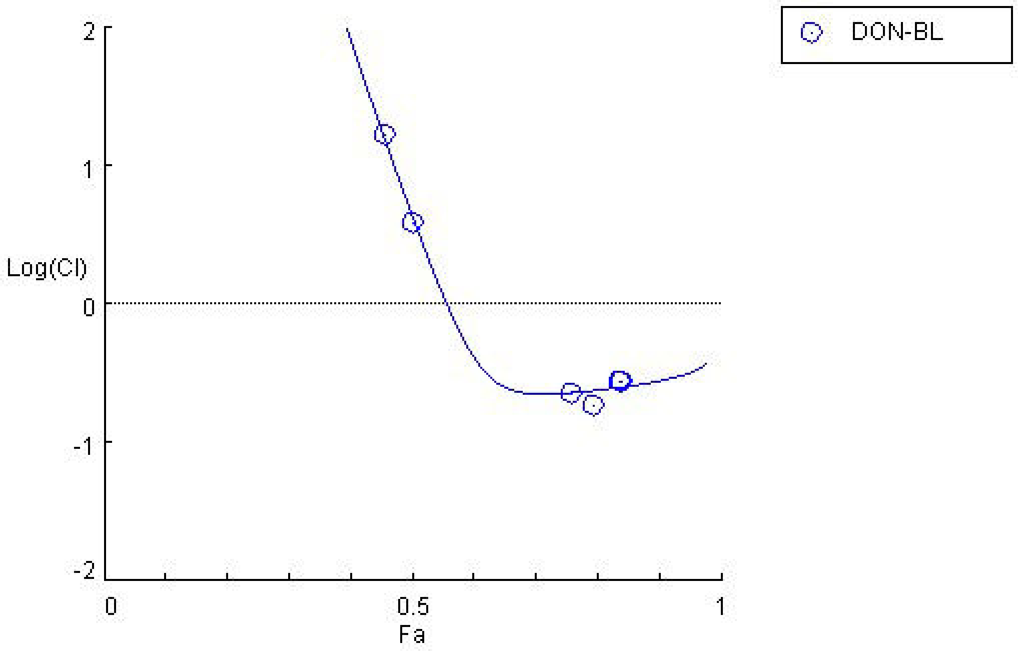

DRI Data for Drug Combo: DON-BL (DON+BL [9.7363:1])

**Fa**	**Dose DON**	**Dose BL**	**DRI DON**	**DRI BL**
0.05	4.3E-11	0.39493	1.1E-10	9.47106
0.1	2.48E-8	0.82847	2.52E-8	8.21517
0.15	1.27E-6	1.31065	7.50E-7	7.52256
0.2	2.47E-5	1.85127	9.64E-6	7.03991

Fa	Dose DON	Dose BL	DRI DON	DRI BL
0.25	2.86E-4	2.46234	7.95E-5	6.66470
0.3	0.00243	3.15911	5.02E-4	6.35333
0.35	0.01696	3.96145	0.00268	6.08315
0.4	0.10451	4.89576	0.01281	5.84076
0.45	0.59761	5.99799	0.05748	5.61741
0.5	3.29925	7.31840	0.25035	5.40685
0.55	18.2143	8.92947	1.09030	5.20418
0.6	104.149	10.9399	4.89407	5.00518
0.65	641.748	13.5200	23.4290	4.80574
0.7	4480.95	16.9538	124.910	4.60137
0.75	38073.8	21.7513	788.600	4.38640
0.8	440916.	28.9309	6500.12	4.15262
0.85	8555568	40.8643	83566.7	3.88618
0.9	4.394E8	64.6476	2484070	3.55854
0.95	2.55E11	135.615	5.950E8	3.08667
0.97	2.35E13	229.743	2.93E10	2.78953

DRI values calculated at experimental points

Fa	Dose DON	Dose BL	DRI DON	DRI BL
0.83571	3412598	36.7164	34228.7	3.58557
0.7925	297443.	27.6346	5966.77	5.39737
0.45699	0.75981	6.16809	0.06097	4.81881
0.83863	4094255	37.5033	41065.8	3.66243
0.75690	52230.7	22.5670	1047.76	4.40760
0.50078	3.38816	7.34110	0.27187	5.73522

DRI Plot for Combo: DON-BL (DON+BL [9.7363:1])

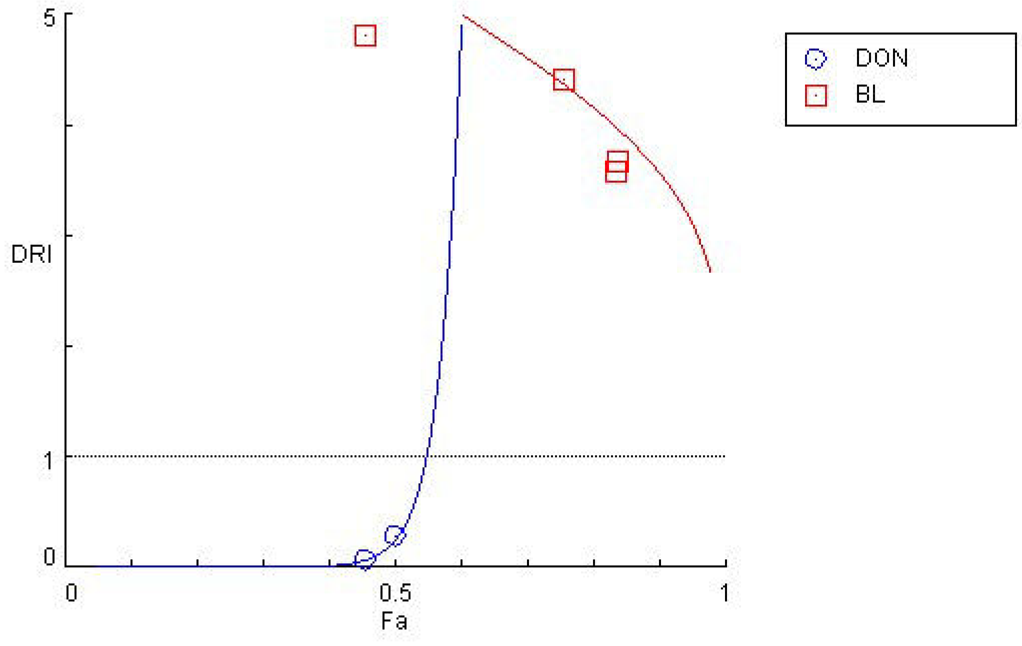

Log(DRI) Plot for Combo: DON-BL (DON+BL [9.7363:1])

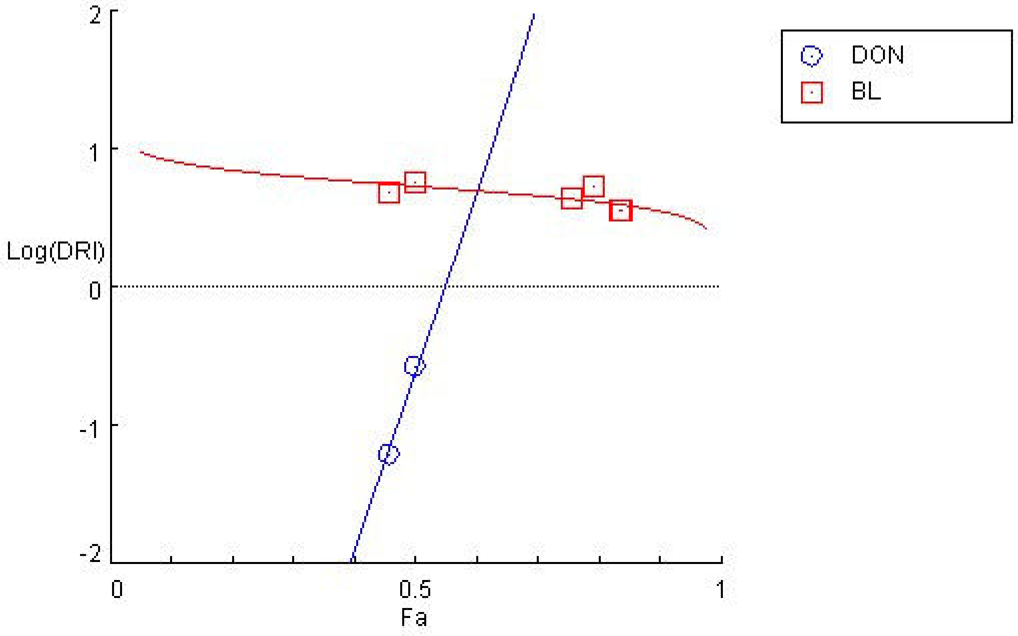

Isobologram for Combo: DON-BL (DON+BL [9.7363:1])

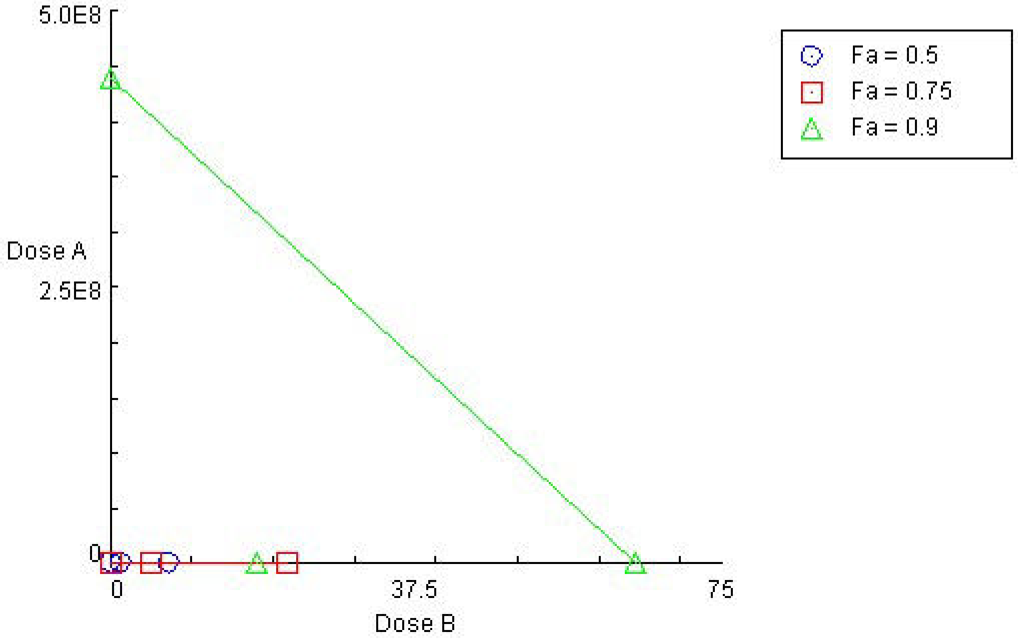

Polygonogram at Fa = 0.9

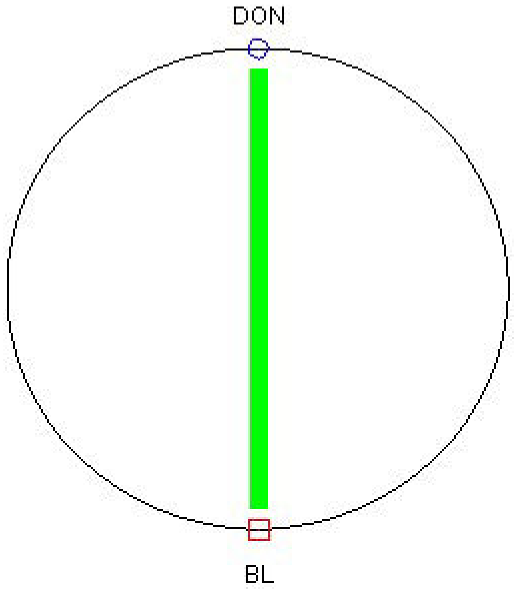
DON
BL
